# Topological coordination numbers and coordination reciprocity from electron-density distributions

**DOI:** 10.1107/S2053273325002347

**Published:** 2025-04-28

**Authors:** Frank R. Wagner, Riccardo Freccero, Yuri Grin

**Affiliations:** a Max-Planck-Institut für Chemische Physik fester Stoffe Dresden Germany; b Dipartimento di Chimica Industriale Universita degli Studi di Genova Genova Italy; Universidad de Oviedo, Spain

**Keywords:** coordination numbers, QTAIM, quantum theory of atoms in molecules, electron density, Voronoi–Dirichlet

## Abstract

Triangulated surface data sets of quantum theory of atoms in molecules (QTAIM) interatomic surfaces have been employed to calculate solid angles subtended at the nuclear positions by each diatomic contact surface. On this basis, topological effective coordination numbers were evaluated. This corresponds to a generalization of the established Voronoi–Dirichlet partitioning (VDP) based procedure. The topological coordination number (tCN) approach developed includes coordination reciprocity requirements necessary to extract coordination-consistent sub-coordination scenarios for identification of chemically meaningful coordination numbers. The ranking between different sub-coordination scenarios is accomplished by weighting functions derived from purely geometrical properties of square and semicircle areas. Exemplary cases analyzed using theoretical electron-density distributions span the range from the face centered cubic, body centered cubic, hexagonal close packed and diamond types of element structures, to rocksalt, CsCl and zincblende types of structures, to compounds of the TiNiSi structure type. An important difference compared with VDP-based coordination numbers arises from the natural inclusion of the effect of different atomic sizes in the tCN approach. Even in highly symmetrical element structures, differences between VDP and tCN results are obtained as an effect of atomic electron-density decay utilizing still available degrees of freedom in the crystal structure. Especially in the TiNiSi type of examples, the advantage of numerically ranking between different sub-coordination scenarios of similar importance emerges. Instead of being obliged to choose only one of them, a more precise characterization contains a listing of different scenarios with their relative weights and associated effective coordination numbers. This seems to be generally the more appropriate way to analyze atomic coordination, especially in more complex structures such as intermetallic phases, opening up its possible use as input for AI applications on structure–property relationships.

## Abstract 2

An approach is presented to calculate topological coordination numbers (tCNs) obeying the principle of coordination reciprocity from solid angles subtended by the interatomic surfaces of electron density (QTAIM) atomic domains. The tCN approach characterizes a compound’s coordination situation as a set of sub-coordination scenarios with associated weights, which is considered a suitable input for future AI applications on structure–property relationships.

## 1. Introduction

The term ‘coordination number’ is one of the key expressions for informational description of a (crystal) structure, *i.e.* an atomic arrangement in the position space. It represents a condensation of the complete geometrical information about the spatial arrangement of the coordinated atoms around a central atom into just one number specifying how many atoms are included in the coordination environment. In a hierarchical description of a (crystal) structure the coordination number of each species represents the primary information. In the handbooks, this term is usually presented as well established, natural and almost obvious, because it may be intuitively understood from the simple examples selected. Nevertheless, there is a need for a powerful mathematical definition not only for the traditional crystallographic and crystal chemical community, but for the whole field of materials science. While the spatial atomic arrangement including the metrics is already specified by the crystallographic parameters of the crystal structure, the ‘coordination number’ of each atom goes beyond it by identifying and counting links between specific atoms. Extraction of the additional information about interatomic links from crystallographic metric information is usually done by an external method not free from bias. The resulting arrangement of atoms and connections allows the creation of a structural graph which opens the door for a digitizable topological description of the structure, useful for advanced information processing technologies like graph neural network and artificial intelligence to predict unprecedented structure–property relations (Reiser *et al.*, 2022 ▸; Zimmermann & Jain, 2020 ▸).

While lattice vectors, symmetry group and list of atomic coordinates uniquely describe the metrics of an atomic arrangement, coordination number (CN) yields primary information about the atomic environment in a condensed form of the number of connected neighbors of a given atom. This was the basic idea behind the original definition of CN as the number of atoms located in the first sphere around an (central) atom (Werner, 1905 ▸). Because in molecular structures, in particular complexes, the atoms of the first coordination sphere are bonded to the central one, the CN from the very beginning was discussed together with the ability of the central atom to form bonds (characterized, *e.g.*, by valence numbers). In the crystal structures of solids, especially in those where the bonding mechanisms and their energetics are not clear or well established, *e.g.* intermetallic compounds, only the metric information mentioned above is experimentally available from standard crystal structure determination methods (*e.g.* structure refinement of X-ray diffraction data) for evaluation of CNs. Thus, the CN and bonding should be separated at the first step of analysis.

The traditional and seemingly obvious approach to evaluate atomic CNs is based on the analysis of the sequence of decreasing interatomic distances *d*(*A*–*B*) (distance string) of the atoms around the central atom *A*. The first coordination sphere can be defined either by the single cut-off value of *d*_min_√2 (*d*_min_ = minimal distance within the distance sequence of atom *A*) for the maximal distance within the coordination sphere [used previously for systematic analysis of intermetallic structures (Kripiakevich, 1977 ▸)] or by allowing a tolerance of *ca* 15% between the distances *d*_min_ and *d*_max_ within the coordination sphere (Zemann, 1966 ▸). Today the most commonly used methods for the purpose of coordination-gap evaluation are the Brunner–Schwarzenbach (BS) (Brunner & Schwarzenbach, 1971 ▸) and the related Brunner ones (Brunner, 1977 ▸), which are based on the appearance of the first large gap in the distance sequence. The BS method is used in the Pearson’s Crystal Data database (Villars & Cenzual, 2023 ▸). Despite the obvious advantage of direct comparability of the results for many compounds, the huge statistical basis also revealed limitations in application. One problem with gap definition concerns the endless sequence of increasing interatomic distances. More than one gap of similar size may appear (a classical example is the α-Fe structure with *CN* = 6 + 8) within this list. Therefore, a cut-off parameter is required to restrict the sequence of distances to be examined for gap formation. Another problematic issue, which is mainly neglected in the literature, is the fact that BS-type CNs are often not pairwise symmetric, *i.e.* for the same distance between atoms *A* and *B*, the species *A* may be accounted for in the coordination sphere of *B*, but not vice versa (coordination reciprocity). An illustrative example is given for CaB_6_ in the supporting information. This deficiency prevents consistent extension of such coordination descriptions by inclusion of bonding information (ultimately, bonding energies) (Menendez Crespo *et al.*, 2021 ▸; Blanco *et al.*, 2005 ▸), which is pairwise symmetric. Coordination reciprocity violating descriptions are therefore difficult to accept from a physical chemistry point of view (O’Keeffe & Hyde, 1982 ▸, 1984 ▸).

The first difficulty mentioned before can be automatically avoided applying the Voronoi–Dirichlet (VD) construction using the geometrical information on the crystal structure described above. VD partitioning (VDP) constructs the spatial domain around each atom at its position (*x*, *y*, *z*) by allocating that region of space closer to this position than to any other atom’s one. Initially introduced for the description of the atomic coordination in complex alloy structures, this technique considers atoms to belong to the coordination of a given species if their VD polyhedra (‘domains’) have a common face (Frank & Kasper, 1958 ▸). The number of faces of a VD polyhedron is uniquely defined, and it represents the maximally possible value of the so-evaluated CN. This number is usually larger than the chemically expected one, based on the number of conceptually explained bonds. The problem of identification of the exact border of the chemically expected coordination sphere (gap) can be considered as the key one. On the other hand, an experimentally determined CN can also challenge chemical understanding instead of always being based on it.

One approach to extract chemically significant CNs was the distinction between counted ‘direct’ and omitted ‘indirect’ neighbors of the central atom, where the ‘direct neighbors’ are characterized by the intersection point of the interatomic connection line located in the common face (Frank, 1967 ▸). Another approach was not to distinguish between ‘direct’ and ‘indirect’ neighbors but to give each neighbor a weight lower than one which sum up to an effective CN. For this purpose, the normalized size of each face of the VD domain was characterized by the solid angle subtended by this face at the central atom (O’Keeffe, 1979 ▸). Actual methods work, for example, with a combination of criteria based on different types of radial overlaps between the central atom and the atoms with common faces of the VD polyhedron (Peresypkina & Blatov, 2000 ▸; Blatov, 2004 ▸), or by combining weighting functions based on solid angles with parameterized correction factors (Pan *et al.*, 2021 ▸). An overview and evaluation of prevalent methods is given by Pan *et al.* (2021 ▸).

In the end, the use of purely metric information in the BS, VD and simpler approaches yields specific kinds of geometric CNs. To involve empirical chemical information in the quantitative characterization of crystal structures, further strategies were applied. The simplest one is the comparison of the experimental distances with the sum of expected radii for the species *A* and *B*, which answers the question about the membership in the coordination sphere for a specific kind of bonding and, at the same time, implicitly suggests this kind of bonding as possible for the *A*–*B* interaction. Such similar considerations are the basis of the very broad definition of CN adopted by the International Union of Crystallography, according to which the CN of an atom in a crystalline solid depends on the chemical bonding model used (Lima-de-Faria *et al.*, 1990 ▸). This has a more descriptive but not conceptual character, and reveals the need for a definition of CN as an observable property.

The various CN values obtained by involving information about chemical bonding are ‘chemical’ CNs in contrast to the ‘geometric’ ones. In the optimal case, they should pave the way for the further analysis of crystal structures with respect to the reasons for the observed metric, its stability and predictability, understanding of the interplay between the crystal structure and physical and chemical properties of the material. According to the Hohenberg–Kohn theorems, the ground-state properties of a system (*e.g.* crystal or molecular structure) are completely defined by its electron-density distribution (Hohenberg & Kohn, 1964 ▸). Together with the variational principle established for trial electron densities, these cornerstones of density functional theory form the basis for quantum chemical calculations of properties for molecular and crystalline solids. Therefore, the development of the basic crystal structure descriptors based on the electron density (ED) would open the way for the understanding of crystal structure metrics, stability and properties on a unified basis of analysis of ED, which is also experimentally accessible (Coppens, 1997 ▸; Gatti & Macchi, 2012 ▸). As a first step, the conceptual replacement of the atomic VD polyhedra by the QTAIM (quantum theory of atoms in molecules) atomic regions (Bader, 1990 ▸) was suggested by Zou & Bader (1994 ▸), further envisaged by Blatov & Serezhkin (2000 ▸), but never quantitatively realized to the best of our knowledge. Such a replacement includes information about the chemical bonding in the system, which can be further visualized and characterized by use of electron pair density derived properties and distributions, like in the electron localizability approach (Wagner & Grin, 2023 ▸). QTAIM atomic regions were used for crystal chemical investigations of atomic volumes, contact radii and effective charges, revealing their chemical relevance (Baranov *et al.*, 2008 ▸; Fedorchuk & Grin, 2018 ▸; Agnarelli *et al.*, 2023 ▸). Since the definition of the CN in the present study is based on the topological analysis of ED and the resulting topological definition of atoms, an approach coined ‘quantum chemical topology’ (Popelier, 2016 ▸), we prefer to call the resulting CNs ‘topological coordination numbers’ (tCNs). The results of the study on the definition and application of tCNs build the content of the present work.

## 2. Computational section

The electronic structures of all investigated compounds have been calculated at the DFT/PBE (Perdew *et al.*, 1996 ▸) level using *FHIaims* (Blum *et al.*, 2009 ▸). All structures have been optimized, besides those of TiNiSi and Co_2_Si adopted from Landrum *et al.* (1998 ▸) and PbCl_2_ adopted from Sass *et al.* (1963 ▸). Crystallographic information on the structure data used is given in the supporting information (Tables S1 and S2).

The ED has been calculated on a 0.05 bohr (0.026 Å) 3D mesh, and the QTAIM basins were determined within this mesh using *DGrid-5.1* (Kohout, 2020 ▸). The critical points (*cp*s) were determined by *DGrid-5.1* starting from mesh points, but refining the positions using wavefunction information, such that the final positions are independent from the mesh. For each compound investigated, except for hexagonal close-packed (*hcp*) Ti, the Poincaré–Hopf relationship (Zou & Bader, 1994 ▸) was fulfilled. This means that the alternating sum of the number of attractors (*att*, (3, −3)), line critical points (*lcp*, (3, −1)), ring critical points (*rcp*, (3, +1)) and cage critical points (*ccp*, (3, +3)) per unit cell is equal to zero:
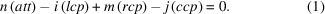
Sometimes situations arise where certain ED curvatures at *lcp*s and *rcp*s are so close to zero that topologically unstable situations show up, *e.g.* very close locations (much smaller than the mesh size employed) of *lcp*s and *rcp*s may not get resolved within the standard *cp* search (*cf.hcp*-Ti below).

The ED basins were visualized with the *AVIZO* software (Avizo, 2018 ▸) with its internal triangulation routines. The *AVIZO* basin surface triangulation was exported to an ASCII surface file. In the *AVIZO* surface file, each basin surface was encoded as a number (5000 to 30000) of triangle (Δ_*i*_) positions. The triangulated basin surfaces were evaluated with the program *QTgeom* (Wagner, 2021 ▸) developed to calculate solid angles of the contact surfaces between QTAIM basins subtended at the central atoms. The solid angle ω_*i*_^Δ^(*A*; *B*) subtended by each QTAIM surface triangle Δ_*i*_ (with vertices located at positions **R**_1_, **R**_2_, **R**_3_ relative to the nuclear position of *A*) at the QTAIM atomic center *A* was calculated according to (Oosterom & Stracke, 1983 ▸)
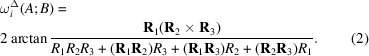


The solid angle ω(*A*; *B*) subtended by the whole interatomic (IA) contact surface at atom *A* was calculated as the sum of the modulus of all such triangle contributions. The sequence of the triangle vertices for each triangle given in the *AVIZO* surface file was not ordered with respect to clockwise or counterclockwise rotation, such that positive and negative values of ω_*i*_^Δ^(*A*; *B*) can result; therefore, the absolute values were added,
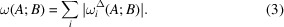


For each atom *A* the sum of ω(*A*; *B*) over all IA surfaces *A*–*B* must be equal to 4π. This sum rule was numerically fulfilled for all compounds and atoms reported with an error of less than 1%.

## 3. Topological coordination numbers

### 3.1. Definition

The QTAIM interatomic contact surface (IA surface) between two connected atoms *A* and *B* may display different values for the solid angles ω(*A*; *B*) (subtended at atom *A*) and ω(*B*; *A*) (subtended at atom *B*). Coordination is symmetrical in the atomic indices (O’Keeffe & Hyde, 1982 ▸, 1984 ▸; Parthé, 1996 ▸), *i.e. A* is coordinated to *B* in the same way, *e.g.* with the same amount of energy, as *B* is coordinated to *A*. In the following, this is denoted as coordination reciprocity (CR). As a consequence, the sum of solid angles subtended at both central atoms *A* and *B* in contact was used (alternatively, the algebraic average could have been used giving the same results) to obtain a solid angle measure ω(*A*, *B*) related to *A*–*B* coordination:
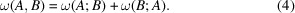
This can be considered as a generalization of the VDP procedure, where ω(*A*; *B*) = ω(*B*; *A*) is always valid (Serezhkin *et al.*, 1997 ▸).

For the topologically defined coordination environment of each species *A*, an ordered string **S**^ω^(*A*) (coordination sequence) of decreasing solid angle values *S_j_*^ω^(*A*) = ω(*A, B_j_*) = ω*_j_*(*A*) is created containing all neighbors with a common QTAIM surface to *A*. The string is always terminated by an additional solid angle value of 0, the value for all neighbors without a contact surface with *A* (in the notation used in the following, it is the string member *k* + 1). The contribution of each *j*th contact (*j* ≤ *k*) in the string to the so-called topological coordination number *tCN*_tot_(*A*) of species *A* is 1, such that the sum of all *k* elements of the coordination environment with *ω_j_*(*A*) > 0 yields
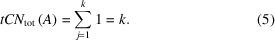
Normalization of the individual values ω*_j_*(*A*) with respect to the largest solid angle ω_1_(*A*) of the string yields the effective coordination contribution *tCN*_*j,j*_^eff,loc^(*A*) of the *j*th ligand [equation (6*a*[Disp-formula fd6a])]. This was initially proposed by O’Keeffe (1979 ▸) in the framework of VDP,
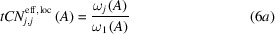

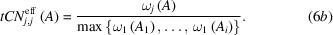


However, taking CR into account, 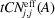 values must be globally normalized within the structure analyzed in order to ensure the same contribution value is added for both sides *A*, *B* of the contact *A*–*B* [equation (6*b*[Disp-formula fd6b])] even in the case of VDP. To the best of our knowledge, this has never been explicitly mentioned in the literature so far. Note, equations (6*a*) and (6*b*) are the same for structures with only one species (O’Keeffe, 1979 ▸). In the present study, equation (6*b*) is used throughout.

The effective contributions of all contacts sum up to the effective topological total CN 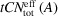 or in short *tCN*^eff^(*A*) [equation (7)]:
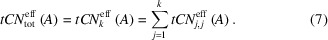
Omitting index *k* from the symbol, specifying *tCN*^eff^(*A*) implies complete summation over all contact surfaces has been performed. Partial sums are generally denoted by two indices 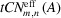 (with {*m, n*} ≥ 1; *n* ≥ *m*; *n* ≤ *k*) and are obtained according to
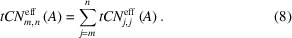
Specification of only one subscript index *n* means complete summation up to *n* starting at *m* = 1.

From a technical point of view, contributions were counted to *tCN*^eff^(*A*) and *tCN*_tot_(*A*) if their individual 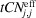 (*A*) values were larger than or equal to 0.5% of the largest value of 1, *i.e.*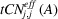 ≥ 0.005.

### 3.2. Coordination reciprocity and coordination scenarios

The fundamental principle of CR plays an important role for setting up coordination scenarios of each specific crystal structure. CR ensures that a contact between species *A*–*B* is counted for the coordination situation of species *A* in the same way, *i.e.* with the same contribution to *tCN*^eff^, as it is counted for the coordination situation of species *B*. In the present context, a coordination scenario is defined to represent a mutually consistent (with respect to CR) combination of individual coordination situations of each atomic species. Disregard of CR would yield an unbalanced overall coordination description (O’Keeffe & Hyde, 1982 ▸, 1984 ▸). For the present study, the following protocol has been set up to account for this basic principle:

(i) Set up the ordered coordination string **S**^ω^(*A*) of decreasing ω*_j_*(*A, B*) values [abbreviated as ω*_j_*(*A*) in the following] to create a coordination sequence for each species in the structure. The last member of the string with nonzero solid angle is denoted the *k*th member in the following. As the final member of each string a coordination-sequence termination member with value ω*_k_*_+1_(*A*) = 0 is added.

(ii) For calculation of 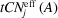 values [equations (7), (8)], the overall largest value ω_1_(*A_i_*) within all species *A_i_* is used as the unique common reference 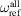 for all solid angles ω*_j_*(*A_i_*, *B*) in the crystal structure analyzed [equation (6*b*[Disp-formula fd6b])]. This leads to an ordered coordination sequence of decreasing values 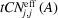 of *globally* normalized solid angles.

(iii) For calculation of coordination probability and likelihood values, the coordination string **S**^ω^(*A*) of each species is *locally* normalized by division by the largest value *S*_1_^ω^(*A*) in the string. This yields the internally (*locally*) normalized string **S**^ω^°(*A*) [equation (9)]:
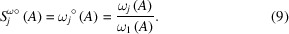
For those species where *S*_1_^ω^(*A*) = 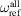, the locally normalized solid angles are equal to the globally normalized ones, *i.e.*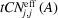 = ω*_j_*°(*A*). This is always valid for at least one species.

(iv) In order to evaluate the coordination gaps between subsequent neighbors of the coordination string, an algorithm based on coordination probability (Zimmermann & Jain, 2020 ▸) has been applied. In this scheme, each neighbor *j* in **S**^ω^°(*A*) with *locally* normalized solid angle weight ω*_j_*°(*A*) obtains a characteristic probability value *p_j_*(ω*_j_*°(*A*)) dependent on its ω*_j_*°(*A*) value and the weighting function *f*(ω°):
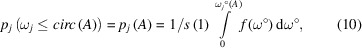
where *s*(1) is the area-normalization value (Appendix *A*[App appa]),
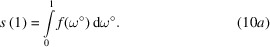


The definite integral over the weighting function [equation (10)] describes an area, and in the semicircle weighting scheme used in the following the areas of semicircle segments are computed (Appendix *A*[App appa]). There are two variants to perform semicircle weighting, which yield different results. Only one variant ‘sc1’ [equation (11*a*)] has been employed up to now (Zimmermann, 2024 ▸; Zimmermann & Jain, 2020 ▸; Pan *et al.*, 2021 ▸); the other one [‘sc2’, equation (11*b*[Disp-formula fd11b])] employed in the present study features a favorable systematic downsizing of coordination gaps with respect to linear weighting [‘lin’, equation (11*c*[Disp-formula fd11c])] between higher neighbors (ω*_j_*°(*A*) < 0.5) within the string (Appendix *A*[App appa]),
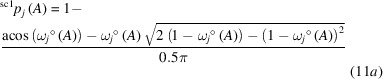

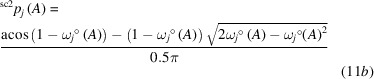

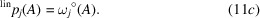


The value *p_j_*(*A*) has been interpreted as the probability of the coordination number *tCN*(*A*) = *j* given by the number of neighbors included in the interval [ω_1_°, *ω_j_*°] of the coordination string **S**^ω^°(*A*) (Zimmermann & Jain, 2020 ▸) for the hypothetical case that *j* + 1 = *k* + 1, *i.e.* that no further members with nonzero solid angles follow.

(v) Evaluation of the likelihood of a certain *CNj* corresponding to calculation of the probability difference between *j* and *j* + 1 coordination. It represents the decisive quantity for characterization of separability of sub-coordinations for each species separately. From the difference between the values ^*scheme*^*p_j_* and ^*scheme*^*p_j_*_+1_ (*scheme* = ‘sc1’, ‘sc2’ or ‘lin’) of subsequent coordination-string members, *i.e.* the *j*-coordination likelihood ^*scheme*^*w_j_* of *tCN*_*j*_(*A*) is obtained. The *j*-coordination likelihood ^*scheme*^*w_j_*(*A*) defined for all *j* ≤ *k* corresponds to the difference between the normalized areas (see Appendix *A*[App appa]) associated with ω*_j_*°(*A*) and ω*_j_*_+1_°(*A*):
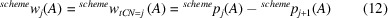
with
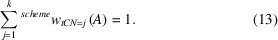


In the present study, all three types of weights, the linear and the two semicircle weights, have been computed in order to assess the variations between the different types of weighting schemes. For each type of weighting scheme, the result is a probabilistic assessment of all different coordination situations of each species *A* separately, from which the one with the highest likelihood and significant difference to the next one defines a well defined coordination situation of species *A*, though independent of the other species. Up to now all species have been considered separately without consideration of CR.

(vi) Impose the consistency of coordination environments of all species with respect to CR. This way, the overall coordination picture can be different from just the sum of the separate species’ coordination preferences. While CR is always fulfilled for the ‘canonical’ scenario with the complete coordination environment **S**^ω^°(*A*) of all species (denoted as ‘tot’ in the following), it is not necessarily fulfilled for any sub-coordination data sets obtained by separate analysis of each species. In the tCN framework, the process to establish consistency of coordination environment is characterized as a search of mutually consistent (with respect to CR) sub-coordination strings 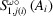 for all species. These sets of mutually consistent sub-coordination strings of all species are called (sub-)coordination scenarios. The sub-coordination string notation 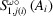 employed indicates that for each species *i* = *A_i_* this sub-coordination string starts at member 1 and ends at member *j*(*i*), where *j*(*i*) is a value specific for each species *A_i_*.

(vii) For setting up a ranking of sub-coordination scenarios, the quantification of the coordination likelihoods ^*scheme*^*w*_*tCN*=*j*__(*i*)_(*A_i_*) [in short ^*scheme*^*w_j_*_(*i*)_(*A_i_*)] for each species *A_i_* is an essential feature to assess the relative importance of each (sub-)coordination scenario as a whole. The total weight *W* of each sub-coordination scenario is computed as a geometric average of the product of the sub-coordination likelihoods of each species according to
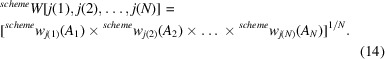


This way, the most likely sub-coordination coincidences typically occur for (consistent) combinations of pronounced (large individual weights ^*scheme*^*w*_*tCN*=*j*__(*i*)_(*A_i_*)) sub-coordination shells of each species *A_i_* = *A*_1_ … *A_N_*. As a result, each sub-coordination scenario is numerically characterized by one value per weighting scheme employed, *i.e.* by three values of the form {^sc2^*W*[*j*(1),…, *j*(*N*)], ^lin^*W*[*j*(1),…, *j*(*N*)], ^sc1^*W*[*j*(1),…, *j*(*N*)]}.

For easier comparison of different scenarios, they were additionally normalized with respect to the most dominant scenario for each weighting scheme. These values are indicated by a superscript according to {…}° (see also Appendix *B*[App appb]).

(viii) Each sub-coordination scenario with weight ^*scheme*^*W*[*j*(1), *j*(2), …, *j*(*N*)] is related to an associated effective *tCN*^eff^ value for each species 1, 2, …, *N*, *i.e.*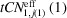, 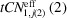, …, which can be calculated according to equation (8). The values obtained for the dominant sub-coordination scenario are denoted 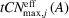, which is different from the sum of neighbors included 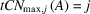, *i.e.*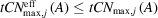. With *tCN*_max_(*A*) and 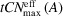 defined as the respective CNs of species *A* in the scenario with the highest topological weight, *tCN*_max–1_(*A*) and 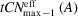 denote these values for the scenario of second highest weight.

Some explanatory notes on the quantities defined in steps (i) to (viii) can be found in Appendix *B*[App appb]. An important aspect of the tCN approach concerns the relation of (sub-)coordination scenario weights and their associated effective CNs for each species. The topological scenario weights give an indication of how well the corresponding coordination scenario is separated from the next one with higher coordination of at least one species. This way the value indicates how well the sub-coordination scenario is defined as a separate partial structure. Another kind (complementary) of information is obtained from the species’ effective CNs, the 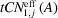 values for each coordination scenario. When compared with their total counterparts 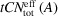 they species-wise quantify the amount of total coordination that is contained in the respective sub-coordination scenario. Considering both types of values together can give rise to counteracting situations in the sense that, *e.g.*, a well defined partial structure (high scenario weight) contains only a small part of the total coordination of the species (and vice versa as well), such that another scenario with a lower topological weight but higher effective tCNs is also significant for the characterization of the coordination situation in the compound’s structure.

## 4. Results

Numerical data for all structures presented are collected in Tables 1 ▸ ▸–3 ▸. More detailed versions of these tables are given in the supporting information (Tables S6–S8).

### 4.1. Elemental 1-species structures

All element structures analyzed in the following display only one type of atomic species. The high symmetry and dense atomic packing in some of these structures lead to highly symmetric VD and QTAIM atomic domains. Nevertheless, they do not display trivial topological duality relations to the associated coordination polyhedra in general (supporting information, Section S3).

The presence of only one atomic species leads to a simplification of the process of calculating sub-coordination scenario weights, because the species’ sub-coordination likelihood ^*scheme*^*w_j_*(*A*) equals the sub-coordination scenario weight ^*scheme*^*W*[*j*(1)] (Table 1 ▸).

**Figure 1:**
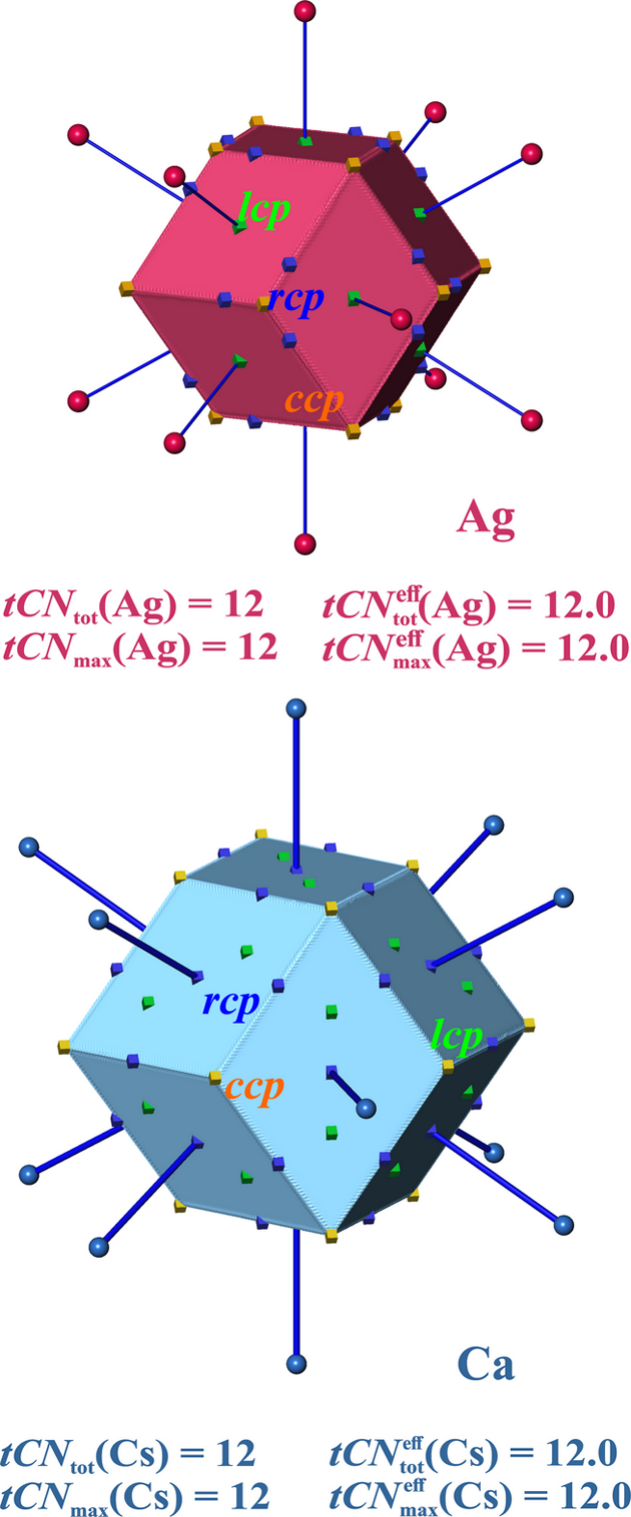
QTAIM domains in the *fcc* structures of Ag and Ca. tCN values displayed correspond to the ‘sc2’ type of weighting scheme (Table 1 ▸). In Ag *lcp*s are found at the centers of the rhombic faces; in Ca *rcp*s are located at this position being connected to two *lcp*s in the two triangular sub-regions of the rhombic faces. Critical points *lcp*s, *rcp*s and *ccp*s are indicated by green, blue and orange cubes, respectively.

*Face centered cubic (*fcc*) structures*. For these highly symmetric element structures with a cuboctahedral coordination polyhedron (Table S4), all vertices are equivalent in accordance with the vertex-transitivity of Archimedean solids. Consequently, the VDP atomic domains with the shape of the rhombic dodecahedron (Catalan solid) display only one type of face (Table S4). For all *fcc* metals investigated (*M* = Al, Ca, Rh, Pd, Ag) the QTAIM atomic domains represent a rhombic dodecahedron with 12 planar faces, which means not only *tCN*_tot_(*M*) = ^VPD^*CN*_tot_(*M*), but also *tCN*_tot_(*M*) = 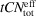 (*M*) = 12 (Fig. 1 ▸). For this situation the canonical coordination scenario is the only one with non-vanishing coordination likelihood and, thus, *W*_*tCN*=12_ = {1, 1, 1}° (Table 1 ▸).

While for most of the examples investigated, each rhombic IA surface was marked by a *lcp* at the intersection point with the IA line, an unexpected *cp* arrangement is found for *fcc*-Ca. The internuclear lines cut the rhombic IA surfaces at a *rcp* instead (where in the other cases a *lcp* was located), but within each triangular sub-area of the rhombic face a *lcp* is additionally found. This is a clear example of the complex and non-unique relation between the location of a specific *cp* (*e.g.lcp*) and the coordination characteristics of the ligand.

*Body centered cubic (*bcc*) structures*. For *bcc* structures two coordination scenarios can be conceived: the *CN* = 8 one from the shorter 1nn (1st nearest-neighbor) contacts and the combined one obtained from *CN* = 8 + 6 by including the next longer 2nd nearest-neighbor (2nn) contacts as well. This way, the candidate coordination polyhedron is either a cube [like in the simple cubic (*sc*) lattice] or a rhombic dodecahedron. The associated dual polyhedra are the octahedron and the cuboctahedron, respectively (Table S4). Note that the 2nn contacts are only 15.5% longer than the 1nn ones, which forms the basis of a long-standing discussion of either inclusion or omission of them in a dualistic argumentative framework.

VDP yields atomic domains with the shape of a truncated octahedron (Table S4) obtained by additional truncation of the *CN* = 8 octahedral VDP domains by the six intersecting planes at the midpoints of the longer contacts. The dual polyhedron of the truncated octahedron is the tetra(kis)hexahedron (Table S4). It is related to the cuboctahedron by being a more general form (with one free parameter, in contrast to the cuboctahedron which has none) obtained during the process of truncating an octahedron.

As exemplary cases for analysis of topological space partitioning by QTAIM atomic domains the *bcc* elemental structures of K and Mo have been chosen. They represent two extremes of very low (K) and high (Mo) average valence EDs.

**Figure 2:**
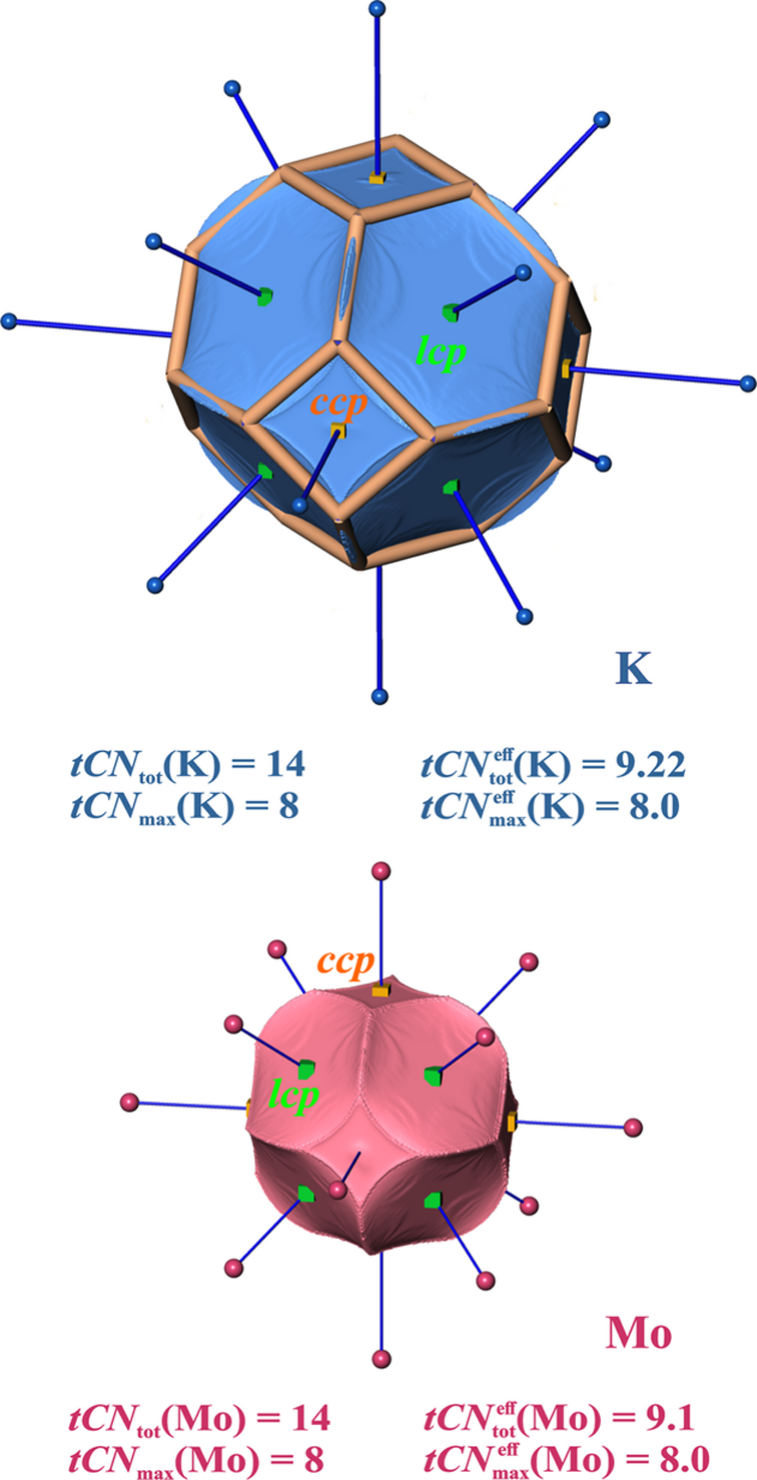
QTAIM domains in the *bcc* structures of K and Mo. tCN values displayed correspond to the ‘sc2’ type of weighting scheme (Table 1 ▸). *Lcp*s are located on the 1nn 6-gonal and *ccp*s on the 2nn 4-gonal faces of the QTAIM domains roughly resembling convex truncated octahedra of the VDP domains (drawn for K).

The QTAIM atomic domains (Fig. 2 ▸) are found to resemble the shape of a truncated octahedron like the VDP ones. Thus, the same overall *CN* of ^VDP^*CN*_tot_ = *tCN*_tot_ = 8 + 6 = 14 is obtained. The *CN* = 14 situation characterizes the size of the atomic coordination sphere (largest gap) in the Brunner, BS and Villars–Daams (Daams & Villars, 1997 ▸) approaches in this structure type. The contribution of the atoms at larger distances to an effective *CN* are specifically down-weighted in these approaches, such that ^BS^*CN*^eff^ = 12.5 (Brunner & Schwarzenbach, 1971 ▸) and ^B^*CN*^eff^ = 11.9 (Bhandary & Girgis, 1977 ▸) were obtained. VDP yields an effective *CN* for *bcc* structures of 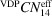 = 8 + 2.16 = 10.16 (O’Keeffe, 1979 ▸; Serezhkin *et al.*, 1997 ▸).

Although the average valence EDs in *bcc*-type K and Mo are very different, in the tCN framework with *tCN*_tot_ = 14 for both compounds virtually the same value of 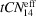 = 8 + 1.2 = 9.2 is obtained. The 2nn contribution obtained is only 56% of the geometrical 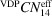 one. One geometrical difference between the planar VDP contact surfaces and the tCN ones is the nonplanarity of the 1nn faces with lips cutting parts of the VDP 2nn surfaces (Fig. 2 ▸). This way, the polygon edges of the 2nn faces are no longer linear as in the VDP case, but curved inwards such that a certain portion of the 2nn faces’ solid angle is lost against the 1nn faces’ ones. Probably more important is the much smaller size of the 4-gonal connecting faces related to the 2nn coordination. This may be related to a stronger than linear decay of the ED in this direction, which is not unexpected given the characteristic exponential decay of the atomic ED in general. In summary, the principal result reported by O’Keeffe (1979 ▸) for the effective *CN* being less than 12 is corroborated with an even larger deviation from 12.

With two different types of faces and solid angles, there are two coordination scenarios, which are directly related to coordination weights because of the presence of only one species. Using linear and semicircle weighting [equations (11*a*), (11*b*), (11*c*)] in the framework of VDP with input values of 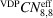 = 1 and 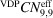 = 0.36 (obtained from O’Keeffe 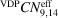 = 2.16), the ^VDP^*CN* = 8 scenario gives a dominant coordination likelihood ^VDP^*W*{*M*^[8]^} = {0.76, 0.64, 0.55} (*i.e.*^VDP^*W*{*M*^[8]^}° = {1, 1, 1}°) and ^VDP^*CN* = 14 a likelihood of ^VDP^*W*{*M*^[14]^} = {0.24, 0.36, 0.45} ({0.32, 0.56, 0.81}°). Although the scenario weights indicate a dominance of the ^VDP^*CN* = 8 scenario for all weighting schemes, the numerical variation between the schemes is rather large.

This dominance of the ^VDP^*CN* = 8 scenario is more pronounced in the tCN framework, which was just shown before to yield smaller effective *CN*s. In the framework of coordination scenarios using three weighting schemes [equations (11*a*), (11*b*), (11*c*)] the following values are obtained: ^*tCN*^*W*{*M*^[8]^} = {0.89, 0.79, 0.74} ({1, 1, 1}°) and ^*tCN*,*scheme*^*W*{*M*^[14]^} = {0.11, 0.21, 0.26} ({0.12, 0.26, 0.35}°) for virtually both, *bcc*-K and *bcc*-Mo (Table 1 ▸).

It is noteworthy that the IA surfaces of the eight nearest neighbors are found to be each marked by a *lcp* with rather different ED values of 0.0025 a.u. (atomic unit) (*bcc*-K) and 0.054 a.u. (*bcc*-Mo), the surfaces for the six 2nn neighbors by *ccp*s of the computed ED with values of 0.0022 a.u. (*bcc*-K) and 0.036 a.u. (*bcc*-Mo) (Fig. 2 ▸).

*Diamond structures*. The VDP domain of diamond C has 4 + 12 = 16 faces (Laves, 1967 ▸; Frank, 1967 ▸) and has the shape of a triakis truncated tetrahedron (Table S4) with 16 faces. Since the corresponding coordination polyhedron is non-convex, a description in terms of convex polyhedra yields a double-shell coordination description employing a smaller tetrahedron inside a larger cuboctahedron (Table S4). This already suggests that the Voronoi polyhedron is mainly related to the dual of the inner polyhedron, *i.e.* another tetrahedron.

The value of ^VDP^*CN*_tot_ = 16 was a severe problem since the *CN* of 12 in the closest packing of equally sized spheres is considered a maximum. Refinement of the VDP-based CN concept considers only ‘direct’ neighbors contribute to the *CN*, omitting the 12 ‘indirect’ ones where the IA lines do not cut the corresponding IA surfaces (Frank, 1967 ▸). Later on, with the concept of relative weight of the surfaces, this problem was resolved in a different way. Using normalized solid angle weights, effective values of 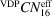 = 4 + 0.54 = 4.54 (O’Keeffe, 1979 ▸) are obtained for all diamond-type structures, and consequently for the zincblende ones as well (Serezhkin *et al.*, 1997 ▸).

**Figure 3:**
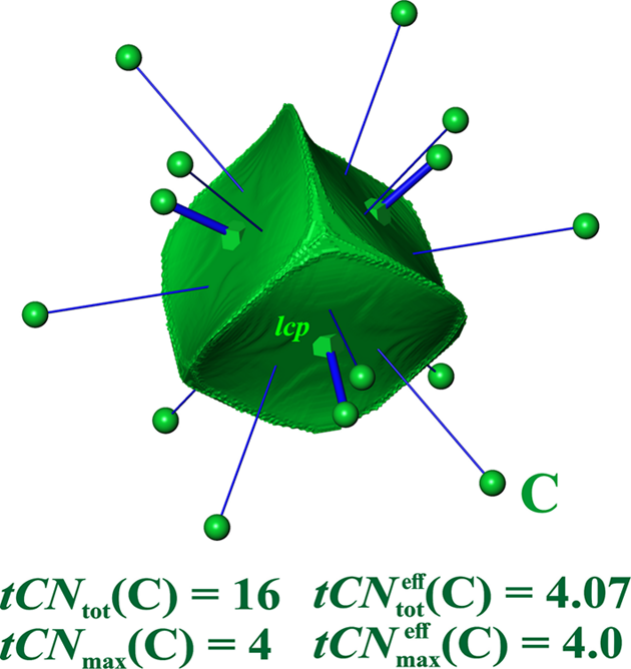
QTAIM domain of carbon in the diamond structure. *tCN* values displayed correspond to the ‘sc2’ type of weighting scheme (Table 1 ▸). The small triakis-truncation faces are recognizable around the threefold axis. The four 1nn atoms yield *lcp*s (green cubes) at the intersection of the internuclear lines (thick blue lines) with associated IA faces of the central atomic domain; the presence of 12 2nn atoms creates the triakis-truncation faces not cut by the associated internuclear lines (thin blue lines).

In the ED-based space partitioning, *tCN*_tot_ = 16 is obtained for all tetrels investigated (Fig. 3 ▸). However, the 2nn contributions are close to vanishingly small (smaller than the VDP ones), and they are not marked by a *cp* (only the four 1nn contacts each display a *lcp*). The 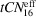 (*E*) values (*E* = C, Si, Ge) are about 4 + 0.1 (Table 1 ▸), *i.e.* the 2nn contribution 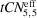 is only 19% of the corresponding 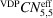 one. The 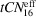 values of different elemental compounds *E* = C, Si, Ge are virtually the same. In the tCN framework, the coordination scenario is dominated by likelihood ^*tCN*^*W*{*E*^[4]^} = {0.999, 0.993, 0.991} (corresponding to normalized {1, 1, 1}°) with ^*tCN*^*W*{*E*^[16]^} = {0.001, 0.007, 0.009} (normalized, {0.00, 0.01, 0.01}°) (Table 1 ▸). In the VDP framework, the coordination likelihood values are only slightly larger than the tCN ones, namely ^VDP^*W*{*E*^[4]^} = {0.99, 0.96, 0.94} (normalized, {1, 1, 1}°) and ^VDP^*W*{*E*^[16]^} = {0.01, 0.04, 0.06} (normalized, {0.01, 0.05, 0.06}°).

*Hexagonal close packing (*hcp*) structures*. The *hcp* structures with 12 nearest neighbors at the same distance display a ratio of hexagonal lattice parameters *c*/*a* = 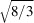 = 1.633 categorized as the ‘ideal’ ratio for closest sphere packing. The coordination polyhedron is an anticuboctahedron. The dual polyhedron of the ideal anticuboctahedron is the trapezo-rhombic dodecahedron containing 6 rhombic and 6 trapezoidal faces (Table S4).

The VD polyhedron of the anticuboctahedral coordination polyhedron also has this shape, where the 6 basal and the 6 apical distances of the central atom are cut by the trapezoidal and rhombic faces, respectively. For ideal *hcp* with *c*/*a* = 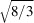 both types of faces display the same area and yield the same solid angle of 4π/12 subtended at the central atom.

**Figure 4:**
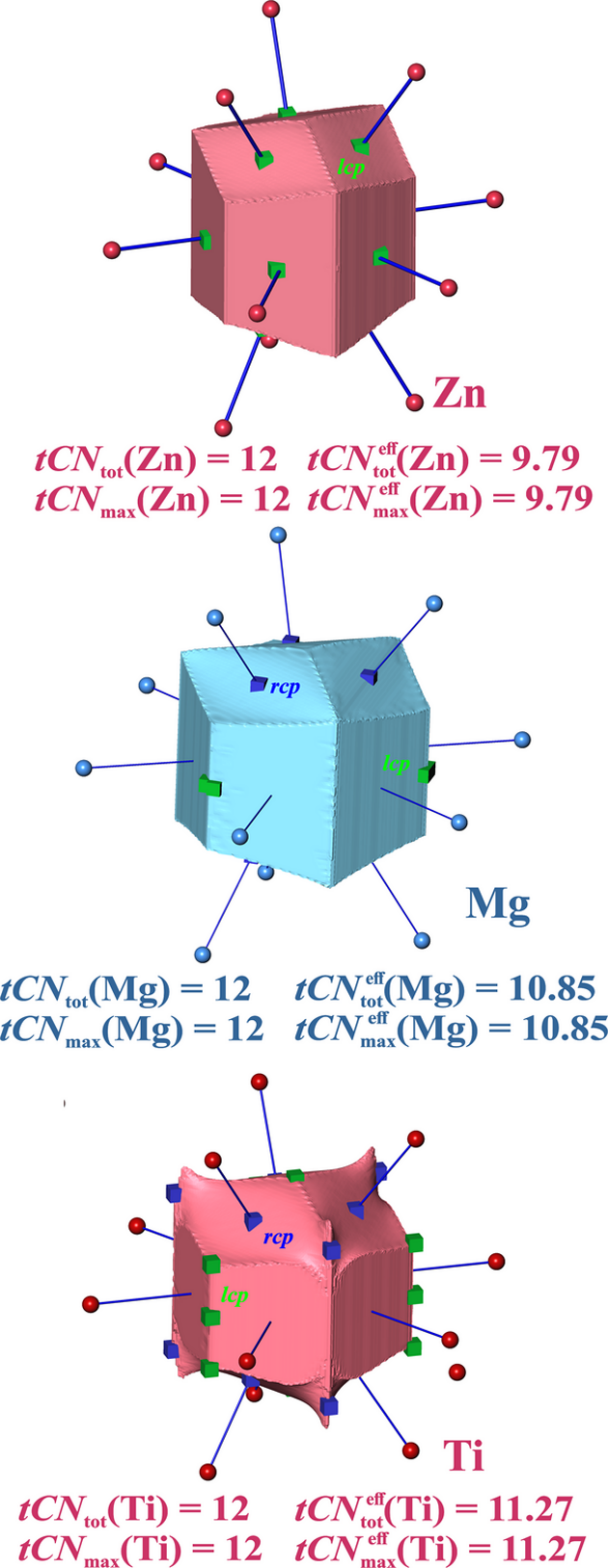
QTAIM domains in the *hcp* structures of Zn, Mg and Ti. *tCN* values displayed correspond to the ‘sc2’ type of weighting scheme (Table 1 ▸). The positions of ED *cp*s are different in all three structures; there is a fit to expectations only for Zn, where *lcp*s on both face types are consistent with *tCN*_max_ = 12.

The varying *c*/*a* ratio of the lattice parameters for different compounds of this structure type makes it less unique compared with the *bcc*, *fcc* and diamond types. For *c*/*a* ratios lower than the ideal one, the basal distances are shorter than the apical ones, leading to larger solid angles of the basal contacts in the VDP framework, and vice versa for larger *c*/*a* ratios. Three element structures have been investigated (Fig. 4 ▸), Mg (*c*/*a* = 1.623, closest to ideal *hcp*), Ti (*c*/*a* = 1.588) and Zn (*c*/*a* = 1.861), which span the range from slightly compressed (Ti) to significantly elongated (Zn) along [001].

With ^VDP^*CN*_tot_ = 12 for all compounds there is always a 6(basal) + 6(apical) coordination found. For Mg (*c*/*a* = 1.623 < 1.633), with only a slight difference between apical (318.3 pm) and basal (319.5 pm) distances, only a small difference between the larger apical and the smaller basal solid angles has been reported, leading to 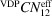 (Mg) = 6(apical) + 5.91(basal) (Serezhkin *et al.*, 1997 ▸).

ED atomic domains for Mg (*c*/*a* = 1.623 < 1.633) display a behavior of solid angle sizes with respect to the IA distances opposite to the expected one found for VDP domains. The solid angles for the shorter contacts should be larger than for the longer contacts. Actually, just the opposite is the case (Fig. 4 ▸), namely for longer basal contacts (319.5 pm) the larger effective coordination increment of 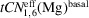 = 6 × 1 = 6 is obtained, and for the shorter apical contacts (318.3 pm) the smaller contribution 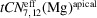 = 6 × 0.81 = 4.9 (Table 1 ▸). The topological coordination difference is surprisingly large. Note that the basal IA surfaces with the larger solid angles do not display a *lcp* close to the intersection of the internuclear line and the IA surface (herein classified as not being marked by a *cp*); the smaller apical ones display a *rcp* (Fig. 4 ▸).

For *hcp*-Ti (*c*/*a* = 1.588) with shorter apical distances, the situation switches to the normal behavior with values for apical (288 pm, *rcp*) 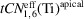 = 6 and basal (293 pm, no *cp* found) 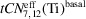 = 5.3 (Fig. 4 ▸, Table 1 ▸) coordination.

For *hcp*-Zn (*c*/*a* = 1.861) with an elongated *c* axis, the basal distances are now clearly shorter, and the basal and apical coordination values are 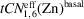 = 6 (266 pm, *lcp*) and 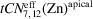 = 3.8 (292 pm, *lcp*).

Summarizing, in all three elemental metals a dominance of the 12-coordination representing 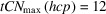 is found. The largest value of 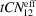 = 11.3 is found in Ti with the most compressed *c*/*a* ratio. It is slightly smaller in ‘closest to ideal’ *hcp*-Mg [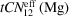 = 10.9] and smallest in the elongated Zn structure [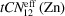 = 9.8]. In all these cases, the values of 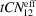 are smaller than in the *fcc* metals [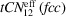 = 12]. Comparison with *bcc* metals with a total of 14-coordination, but with a dominating 8-coordination, is interesting. The 2nn distance in *bcc* structures is 15.5% longer than the 1nn distance, while in the *hcp*-Zn case there is only a 9.5% elongation of the 2nn distance. The latter displays a dominating 12-coordination with a larger effective total CN than the *bcc* metals’ one [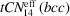 ≃ 9.2], despite its lower total coordination number of 12.

Coordination likelihoods obtained from all three weighting schemes favor the *tCN* = 12 scenario in the *hcp* structures investigated, but the preference decreases along the sequence Ti, Mg and Zn (Table 1 ▸). For *hcp*-Zn the 12-coordination preference becomes rather small (55% versus 45%) with the ‘sc2’ type of weighting [equation (11*b*)].

Thus, there is a systematic dependence of the scenario weights on the *c*/*a* ratio. *Hcp*-Mg, being close to the ideal *hcpc*/*a* ratio, not only strongly deviates from an equal weighting of apical and basal faces, but additionally the basal neighbors with the larger distance display the larger solid angle weights and thus larger effective CN increments 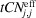.

A remarkable observation concerns the marking of the IA surfaces by *cp*s (Fig. 4 ▸). For the Zn case only, the 6 + 6 surfaces are each marked with a *lcp* located close to the point where the IA line intersects the IA face. In the Mg and Ti case, the basal planes do not display a *cp* on the surface clearly related to the associated neighbor atom, *i.e.* the IA line intersects the IA face far away from any *cp*. These examples clearly demonstrate that the marking of IA surfaces with a *cp* (of any kind) should not be made a primary condition for a topological coordination definition. The situation found for *hcp*-type Mg and Ti is probably connected with polycentric density overlaps in these regions caused by the *AB* type of layer stacking. In contrast, the situation for *fcc* metals displaying the *ABC* type of stacking is found to be more regular (except *fcc*-Ca).

### 4.2. Binary 2-species structures

The binary compounds investigated belong to the rocksalt, CsCl and zincblende structure types. With the exception of the rocksalt type of compounds, they are substitution variants of unary compounds analyzed above. All of these highly symmetric structures display commutative partial structures such that the VDP scheme does not detect any difference between the two species, and the ^VDP^*CN*s are exactly equal to the ones of their unary counterparts (Serezhkin *et al.*, 1997 ▸). Scenario weights {^sc2^*W*, ^lin^*W*, ^sc1^*W*}° normalized with respect to the dominant scenario of each weighting method are employed for each compound (Table 2 ▸). This allows easier comparison of different sub-coordination scenarios and between different compounds. CR is automatically fulfilled in these cases because there is only one type of cation–anion contact (high symmetry) dominating both species’ coordination strings, *i.e.* the solid angle value ω(cation, anion) is always larger than the (one or two types of) homoatomic one.

*Rocksalt type of structures*. The unary variant of the rocksalt type of structure is the simple cubic lattice. The coordination polyhedron encompassing the first 6 neighbors has the shape of an octahedron. The VDP domain associated with this lattice has the shape of a cube, which is the dual polyhedron of the octahedron (Table S4). Thus, for rocksalt structures VDP generally yields only 6 heteroatomic contacts and no homo­atomic ones, such that ^VDP^*CN* = 6 = ^VDP^*CN*^eff^ (Serezhkin *et al.*, 1997 ▸).

In the QTAIM framework the compounds NaCl (*B*_1_ type), RbF (*R*_1_ type), LiI (*B*_2_ type), KI (*B*_1_ type) and RbI (*B*_1_ type) have been analyzed in the present study. They have been assigned to special classes *B*_1_, *B*_2_ and *R*_1_ (indicated in brackets) depending on the locations of the ED *cp*s by Pendás *et al.* (1998 ▸). These *cp* locations are confirmed by the present study. The case of LiI has already been analyzed in detail by Martin Pendás *et al.* (1997 ▸), where the *lcp*s at the 2nn I–I contact surfaces were interpreted as indicating 18-coordinated I atoms, and the absence of Li–Li contact surfaces indicating 6-coordinated Li atoms.

**Figure 5:**
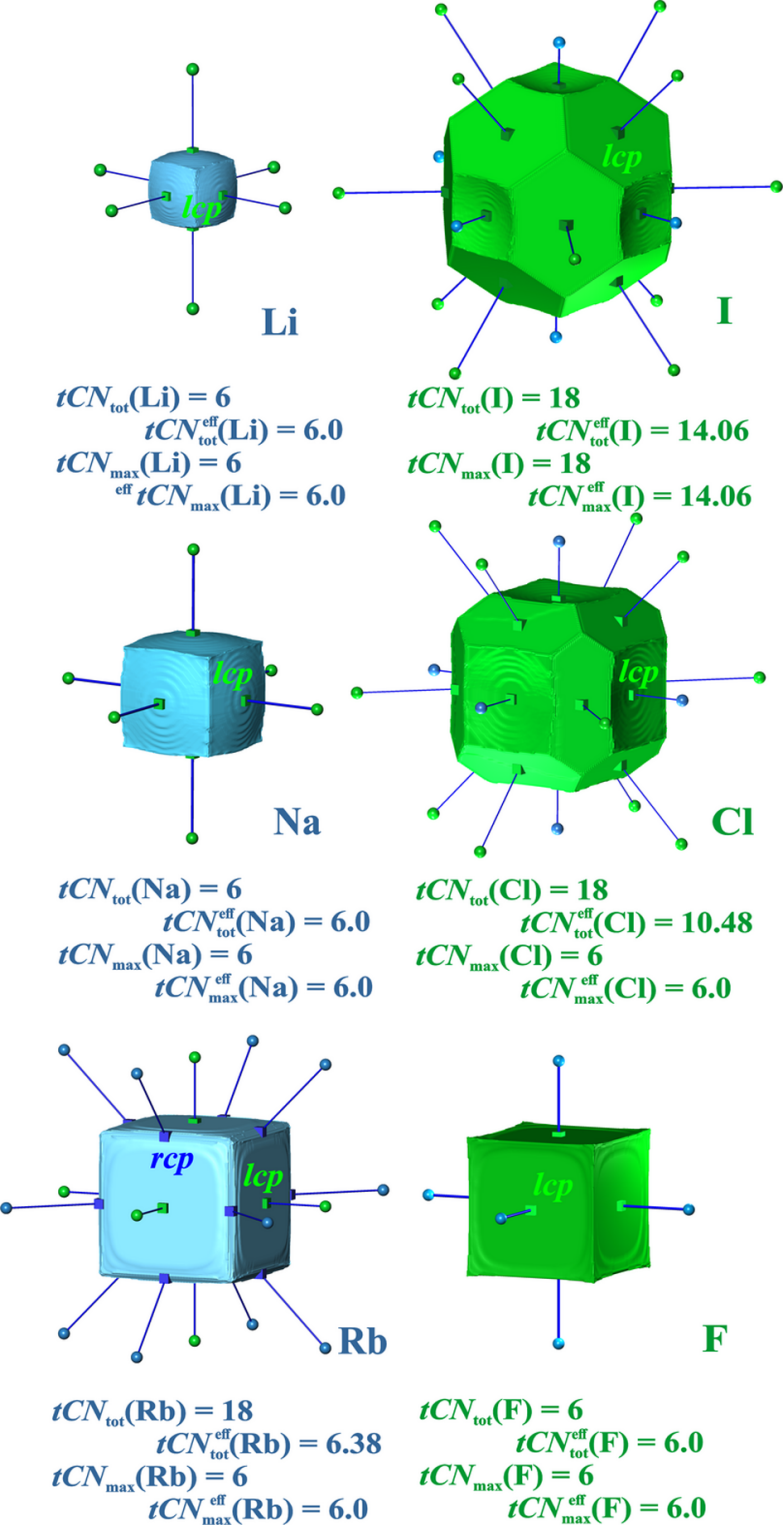
*tCN*s in rocksalt-type structures: (top) LiI; (middle) NaCl; (bottom) RbF. *tCN* values displayed correspond to the ‘sc2’ type of weighting scheme (Table 2 ▸). All 1nn faces are marked by *lcp*s; the 2nn *an*–*an* faces are marked by *lcp*s in LiI and NaCl, the 2nn *ca*–*ca* faces by *rcp*s in RbF.

In all cases besides RbF, the QTAIM atomic volumes of the anions (*an*) are larger than those of the cations (*ca*) (Fig. 5 ▸, Table 2 ▸). The smaller species (typically the cations) display only 6 heteroatomic *ca*–*an* contacts [*tCN*_tot_(*A*_small_) = *tCN*_max_(*A*_small_) = 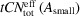 = 6], the larger ones (typically the anions *an*) 6 heteroatomic and 12 homoatomic ones [*tCN*_max_(*A*_large_) = 18] (Table 2 ▸). Consequently, the 12 *an*–*an* contacts lead to additional 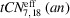 contributions. The largest contributions are found for LiI, where the sum 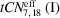 = 12 × 0.67 = 8.1 is even larger than the primary 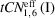 = 6 × 1 one, even though the increment itself is smaller. The smallest *an*–*an* contributions are found for RbI with 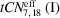 = 12 × 0.2 = 2.28. For RbF, with Rb species being even slightly larger than F, the most similar cation and anion size is found. Here, the homoatomic *tCN*^eff^ contributions are found to be of *ca*–*ca* type. Its value 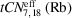 = 12 × 0.03 = 0.4 is the smallest homoatomic contribution among the isostructural compounds investigated, which is consistent with the smallest atomic volume difference (Table 2 ▸).

Analysis of coordination scenario weights using 3 different weighting schemes [equations (11*a*)–(11*c*)] yields only for LiI a dominating (6 + 12) coordination scenario {Li^[6;0]^ I^[6;12]^} (with all 3 weighting schemes) (Table 2 ▸); the remaining ones display a dominant {*spec1*^[6*spec2*; 0*spec1*]^*spec1*^[6*spec2*; 0*spec1*]^} coordination. This is caused by the size difference between the cationic and anionic species, which is extraordinarily large for LiI. Within the four compounds investigated, a trend exists according to which a larger weight ^*scheme*^*W*{*spec1*^[6;0]^*spec2*^[6;12]^} (Table S7) of the 6–18 coordination scenario is correlated with a larger ratio of the sizes (atomic volumes) *V*(*spec1*) / *V*(*spec2*) of the smaller and larger species *spec1* and *spec2*, respectively. Only for LiI is this weight ^*scheme*^*W*{*spec1*^[6;0]^*spec2*^[6;12]^} large enough to become the dominant one.

**Figure 6:**
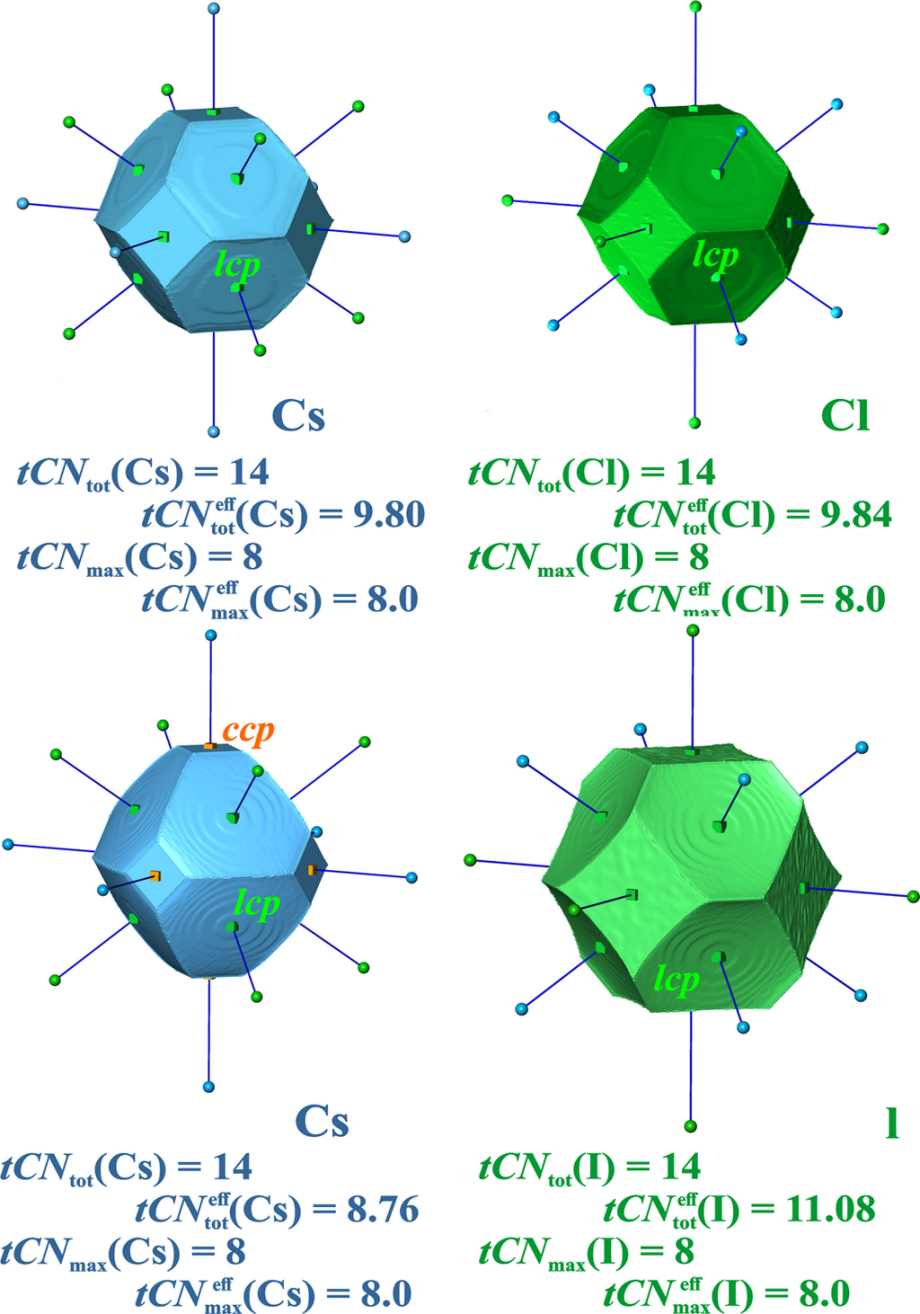
*tCN*s in CsCl-type structures: (top) CsCl, (bottom) CsI; *tCN* values displayed correspond to the ‘sc2’ type of weighting scheme (Table 2 ▸). With the exception of Cs in CsI, where the 2nn faces are marked with *ccp*s, all IA surfaces of the cationic and anionic species are marked by *lcp*s.

*CsCl type of structures*. The binary substitution variant of the *bcc*-type structures is the CsCl type of structure. The unique value 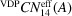 = 8 + 2.16 for this structure type is the same as for *bcc* cases. CsCl and CsI have been investigated using QTAIM atomic domains (Table 2 ▸). The QTAIM atomic domains in both compounds display 8 heteroatomic contacts *ca*–*an*, and 6 homoatomic contacts of types *ca*–*ca* and *an*–*an* (Fig. 6 ▸). The 8 closest *ca*–*an* contacts are the most important ones for both compounds, *i.e.*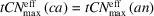 = 8. For CsCl, both atom types display rather similar QTAIM volumes (Table 2 ▸, Fig. 6 ▸) and similar homo-ionic contributions to the respective 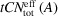, *i.e.*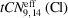 = 6 × 0.31 = 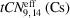. For CsI the larger size of the iodide anion leads to an *an*–*an* contribution of 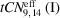 = 6 × 0.51 in the summation for 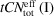, while the *ca*–*ca* contribution of 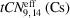 = 6 × 0.13 to 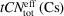 is smaller.

For each of the compounds four (sub-)coordination scenarios are obtained and analyzed: {*ca*^[8*an*; 0*ca*]^*an*^[8*ca*; 0*an*]^}, {*ca*^[8*an*; 0*ca*]^*an*^[8*ca*; 6*an*]^}, {*ca*^[8*an*; 6*ca*]^*an*^[8*ca*; 0*an*]^} and {*ca*^[8*an*; 6*ca*]^*an*^[8*ca*; 6*an*]^}. This is just twice the number of possibilities of the *bcc* cases, {*M*^[8]^} and {*M*^[14]^}, because of the presence of two different species. The difference between CsCl and CsI observed for the effective CNs is also seen in the weights of the coordination scenarios (Table 2 ▸). The {*ca*^[8*an*; 0*ca*]^*an*^[8*ca*; 0*an*]^} scenario is less dominant for CsI and competes with the {*ca*^[8*an*; 0*ca*]^*an*^[8*ca*; 6*an*]^} one, which even dominates with ‘lin’ and ‘sc1’ types of weighting [equations (11*a*), (11*c*)]. The observation that CsCl features cations and anions of rather similar size facilitates direct comparison with the results for the *bcc* elemental structures above without taking size effects into account. It is noteworthy that the {Cs^[8Cl; 6Cs]^ Cl^[8Cs; 6Cl]^} coordination scenario of the ionic species displays a higher relative weight with respect to the dominating 8-coordination {*ca*^[8*an*; 0*ca*]^*an*^[8; 0]^}, than {*M*^[14]^} in the *bcc* metals K and Mo. This is also seen from the average values *tCN*_14_(avg.) = [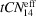
(Cs) + 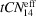
(*an*)]/2 ≃ 10 for both CsCl and CsI, while it is 9.2 for *bcc*-K and Mo. In CsI, the effective cationic coordination 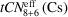 = 8.8 is slightly smaller than 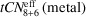 = 9.2 in *bcc*-K, Mo, which is overcompensated by the large value 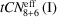 = 11.1 of the anion.

It is instructive to remember that the 2nn distance Cs–Cs in CsCl (420 pm) is much smaller than the 1nn distance in metallic *bcc*-Cs [520 pm (Barrett, 1956 ▸)]. On the other hand, it may also suggest that additional factors should be included like the average ED on the contact surface, which is not considered here, as exemplified by the ED at the *cp*s on the surface. It is much higher in *bcc*-Mo than in the other cases.

On the basis of *lcp*s detected on all 14 QTAIM IA surfaces, CsCl and CsI were previously reported to belong to the so-called ‘B-type family’ with cationic and anionic 14-coordination of B2-type crystal structures (Pendás *et al.*, 1998 ▸). While for CsCl 14 *lcp*s are also found on the IA surfaces of both species in the present calculations, for CsI this is only the case for the iodine domains, and *ccp*s are found at the 6 Ca–Ca contacts (Fig. 6 ▸). This discrepancy indicates a certain case-dependent sensitivity of this topological feature used to distinguish between 8 and 14 coordination of Cs in CsI.

**Figure 7:**
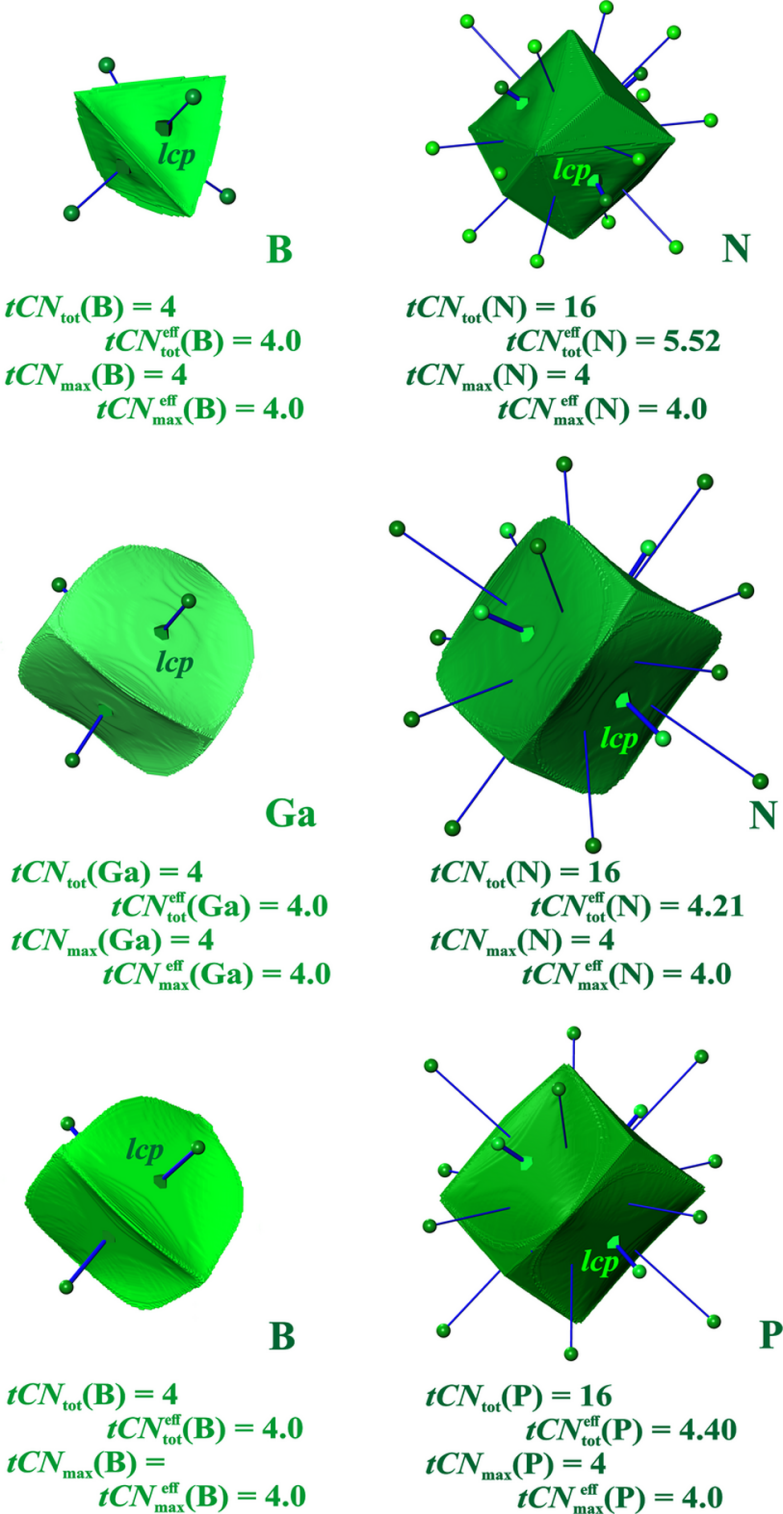
QTAIM atomic domains and *tCN*s in ZnS-type structures. *CN* values displayed correspond to the ‘sc2’ type of weighting scheme (Table 2 ▸). (Top) BN; (middle) GaN; (bottom) BP. The four 1nn faces display *lcp*s at the intersection of the internuclear (thick blue) lines with the IA surfaces, the IA surfaces with the 12 2nn ligands do not display *lcp*s associated with the internuclear (thin blue) lines.

*Zincblende type of structures*. The ^VDP^*CN* values for all compounds of this type are exactly the same as for diamond, *i.e.*^VDP^*CN*(cation) = ^VDP^*CN*(anion) = 4 + 12, and ^VDP^*CN*^eff^(*ca*) = 4 + 0.54 = ^VDP^*CN*^eff^(*an*). In the ED-based space partitioning of BN, BP and Ga*E* (*E* = N, P, As, Sb) the anion is always found to be larger than the cations (Fig. 7 ▸, Table 2 ▸). The cations’ atomic domains display only 4 *ca*–*an* IA surfaces *tCN*_tot_(*ca*) = 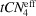 (*ca*) = 4 (for GaN a vanishingly small *ca*–*ca* contact is found). The anions’ atomic domains all display 4 + 12 contact surfaces [*tCN*_tot_(*an*) = 16], with the 4 *ca*–*an* contacts being the most important ones [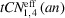 = 4]. The size of the 12 *an*–*an* contributions to 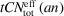 weakly depends on the size difference (ratio of QTAIM atomic volumes) between *ca* and *an* species. The largest volume difference is found for BN [*V*(*ca*)/*V*(*an*) = 0.16], where the effective coordination contributions of the 12 *an*–*an* contacts sum up to 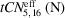 = 1.5. The smallest difference is found for GaN [*V*(*ca*)/*V*(*an*) = 0.75], 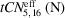 = 0.2. In all binary cases investigated, the summed homoatomic 2nn effective coordination contributions 0.2 ≤ 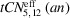 ≤ 1.5 are larger than in the unary diamond types with *tCN*^eff^(*E*) ≤ 0.1 (Table 2 ▸). In terms of coordination scenarios, these findings lead to a clear dominance of the {cation^[4;0]^ anion^[4;0]^} scenario in all cases analyzed, where the largest normalized weight of 24% for the alternative scenario {cation^[4;0]^ anion^[4;12]^} is found for BN (Table 2 ▸). Note that in all the unary and binary cases of the diamond type of structure analyzed, no *cp*s mark the 2nn IA surfaces.

### 4.3. 3-Species compounds with the TiNiSi type of structure

As an application challenge, exemplary representatives of the very common ternary structure type TiNiSi [of which there are several hundreds of representatives (Dshemuchadse & Steurer, 2015*a* ▸,*b* ▸] [*oP*12-(4*c*)^3^ Pearson symbol extended by Wyckoff sequence (Parthé, 1996 ▸)] with comparably low symmetry (site symmetry *m*) have been investigated. The selection of a ternary structure type was made to demonstrate the decisive influence of explicitly taking CR into account. Some binary compounds like PbCl_2_ and Co_2_Si belonging to this atomic arrangement in a wider sense (branches) were also included, where the two species with the same chemical symbol are located on different sites of the TiNiSi type of structure.

In the previous examples the sub-coordination scenarios were not affected by the CR condition. For the 1-species elemental structures analyzed CR is trivially fulfilled, and for the 2-species structures analyzed CR was caused by the high symmetry and the dominance of the species1–species2 contact compared with homoatomic contacts. The equiatomic stoichiometry of three different species in the TiNiSi type of structure makes it easy to check mutual coordination conditions, while they display a non-trivial coordination entanglement originating from CR due to lower symmetry and competing hetero-species’ contacts with similar 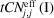 values, as shown in the following.

**Figure 8:**
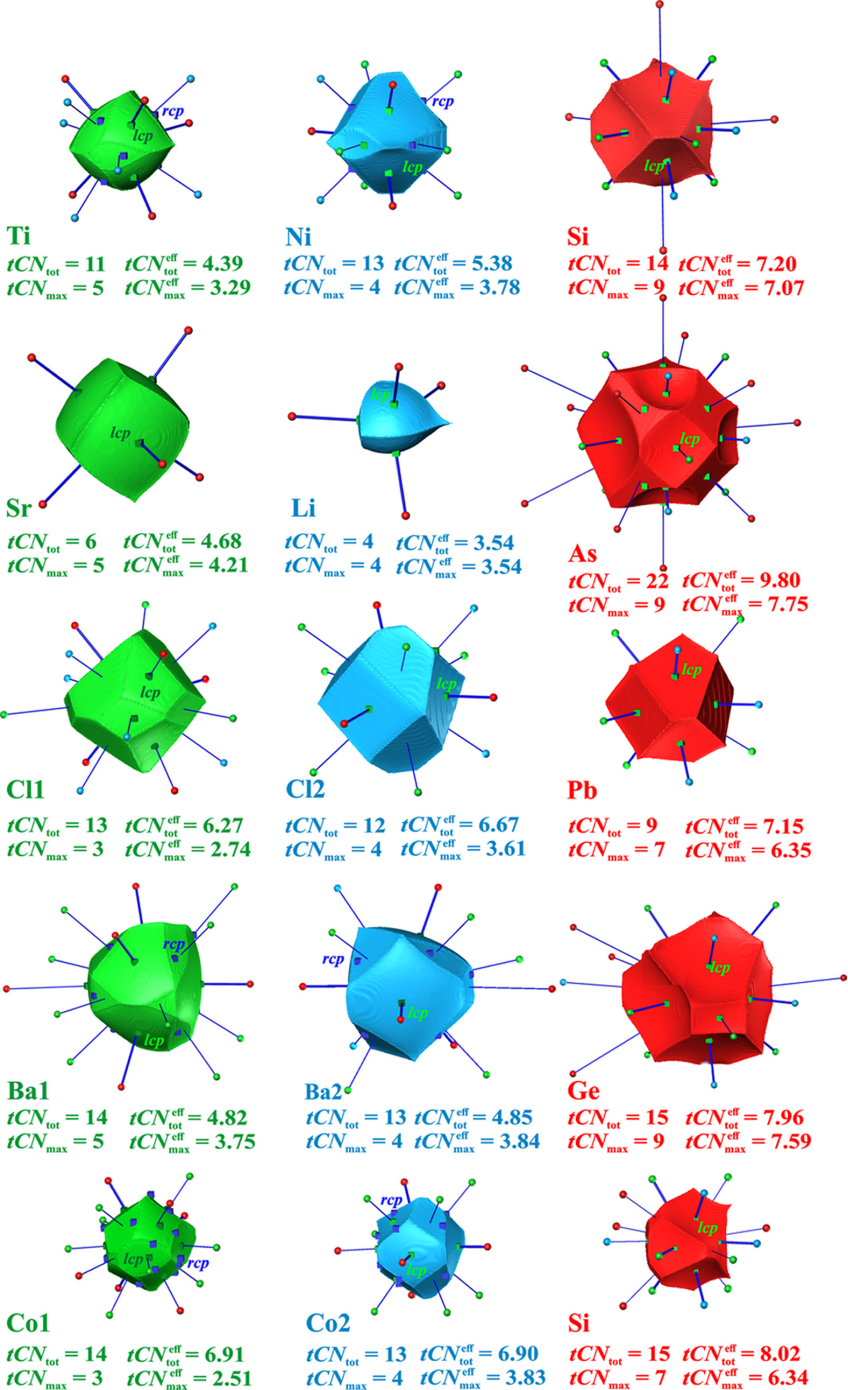
QTAIM basin shapes of TiNiSi-type compounds TiNiSi, SrLiAs, PbCl_2_, Ba_2_Ge and Co_2_Si (from top to bottom). Crystallographically similar species are placed in the same column; spatial orientation is the same for each species type. *Cp*s are shown only if a connection to coordination can be geometrically assumed from the proximity of the intersection point of the IA line with the IA surface. *tCN* values displayed correspond to the ‘sc2’ type of weighting scheme (Table 3 ▸).

The compounds selected with the TiNiSi type of structure can be additionally considered as real challenges for the CN and structure type concept (Jeitschko & Altmeyer, 1990 ▸; Freccero *et al.*, 2023 ▸; Höhn *et al.*, 2025 ▸). The shapes of the QTAIM atomic domains and a depiction of the coordination environments obtained from common IA surfaces with the central atom are given in Fig. 8 ▸. A certain similarity of the basin shapes between the ‘*Si*’ type of species can be seen, although significant differences are present as well. The simplest shape has the Pb basin in PbCl_2_, which looks rather similar to the Si one in TiNiSi. For the other compounds, this basin type is seen to have additional faces, *e.g.* to ‘*Ti*’-type neighbors. The two rather similar side faces in PbCl_2_ and TiNiSi (Fig. 8 ▸) are most different in SrLiAs, where the larger (left) one is connected with Sr, and the smaller (right) one to Li. The dominant tetrahedral coordination at the ‘*Ni*’ site is best seen with Li in SrLiAs. Interestingly, this ‘NiSi’_4_ (‘*Ni*’ = Li, ‘*Si*’ = As) tetrahedron is oriented in the opposite direction in PbCl_2_ (site Cl2, ‘*Ni*’ = Cl2, ‘*Si*’ = Pb).

These remarks on the rather different basin shapes of crystallographically similar types of species may be sufficient to indicate the challenge this structure type poses for any type of systematical coordination analysis. A quantification of the basic coordination features is essential to uncover the similarities and differences in these compounds. Exemplary numerical results for the Si coordination string in TiNiSi are given in the supporting information (Section S4).

**Figure 9:**
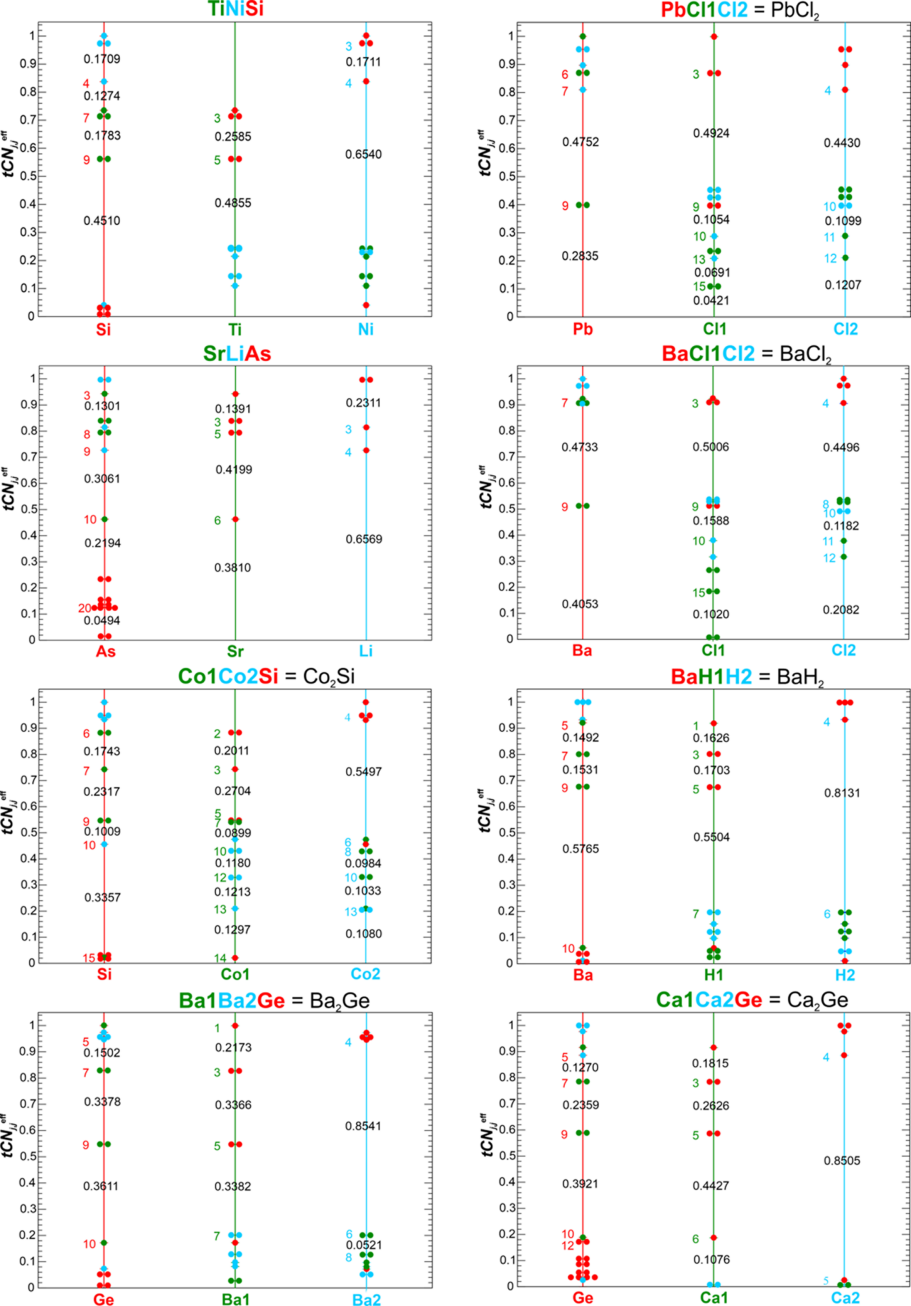
Sub-coordination scenario diagrams for TiNiSi-type compounds. The dots on each species’ coordination string (colored vertical line; red: ‘*Si*’ species, green: ‘*Ti*’ species, cyan: ‘*Ni*’ species) indicate 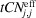 values for (one or two) neighbors of a certain species type specified by the same color code. Coordination likelihood values ^sc2^*w_j_*(*A*) characterizing the local coordination gap are displayed on the vertical bar for each species. Integral *tCN*_1,*j*_ values are given along the coordination string for each species.

CR is decisive in a number of cases. For graphical representation of the coordination situation sub-coordination scenario diagrams (Fig. 9 ▸) are shown for a number of representative cases. For each species *A_i_* (in the present case *i* = 1, 2, 3) the decreasing coordination string elements *S_j_*^ω^(*A_i_*) have been converted into a string of decreasing 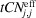 (*A_i_*) values by global normalization [equation (6*b*)[Disp-formula fd6b]]. In the sub-coordination scenario diagrams this 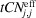 (*A_i_*) string is displayed by dots for each species on a vertical line. The color of the vertical lines is characteristic for the species, the colors of the dots are characteristic for the respective neighbors. The numbers written on the vertical lines are the coordination likelihood values ^sc2^*w_j_*(*A_i_*) [equation (12)] for weighting scheme ‘sc2’. They quantify the coordination gaps. In these diagrams, valid sub-coordination scenarios, *i.e.* those obeying CR, are obtained from horizontal lines touching at least one 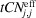 (*A_i_*) value of any species *A_i_*. This 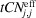 (*A_i_*) value represents the characteristic sub-coordination scenario value 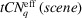, where *q* enumerates all possible sub-coordination scenarios. For each species *A_i_*, all neighbors on and above this line, *i.e.* with values 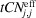 (*A_i_*) ≥ 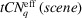, belong to this sub-coordination scenario *q*. CR ensures that contacts between two different species always occur on both species’ 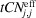 string and with the same value such that they are both either included or excluded in the respective sub-coordination scenario. The coordination gaps ^*scheme*^*w_n_*(*A_i_*) [*n*-coordination likelihood of species *A_i_*, equation (12)] are calculated for each species separately. They depend on the locally normalized values [equation (9)] 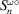 (*A_i_*) and 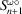 (*A_i_*) for the last neighbor number (*n*) included and the next neighbor (*n* + 1) skipped in a functional form specified by the weighting ‘*scheme*’. As an example, for PbCl_2_ a scenario occurs at scenario value 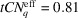, which leads to *tCN*(Pb) = 7 (from 3 Cl1 and 4 Cl2 contacts), *tCN*(Cl1) = 3 (Pb contacts) and *tCN*(Cl2) = 4 (Pb contacts). The coordination likelihood values are 0.4752, 0.4924 and 0.4439 for Pb, Cl1 and Cl2, respectively, which yields after geometrical averaging [equation (14)] a scenario value of ^sc2^*W*{Pb^[3Cl1, 4Cl2; 0Pb]^Cl1^[3Pb, 0Cl1; 0Cl1]^ Cl2^[4Pb, 0Cl1; 0Cl2]^} = 0.47 that is normalized to ^sc2^*W*{Pb^[3Cl1, 4Cl2; 0Pb]^ Cl1^[3Pb, 0Cl1; 0Cl1]^ Cl2^[4Pb, 0Cl1; 0Cl2]^}° = 1 because it corresponds to the scenario (denoted ‘max’) with the highest value using this weighting scheme (Table 3 ▸).

Inspection of all coordination scenario diagrams (Fig. 9 ▸) reveals that species ‘*Si*’ plays a central role in this structure type, especially if it displays the largest QTAIM volume among the three species, which is the case for TiNiSi, SrLiAs, Ba_2_Ge, Ca_2_Ge and BaH_2_. In these cases, for conceptually important scenarios with high coordination-gap weights, the ‘*Si*’ effective *CN* is mainly given by the sum of associated effective *CN*s of its ‘*Ti*’ and ‘*Ni*’ ligands, and in the most relevant sub-coordination scenarios the ‘*Si*’ species represents effectively the highest coordinated species in this structure type (Table 3 ▸). In those cases where ‘*Si*’ displays the smallest species, Co_2_Si, PbCl_2_, BaCl_2_, its coordination dominance is still obvious in scenarios with low *CN*s and the ‘max’ scenario. In the coordination scenarios with high *CN*s the effective coordinations of all three species become more equal due to contacts among ‘*Ti*’ and ‘*Ni*’ species.

**Figure 10:**
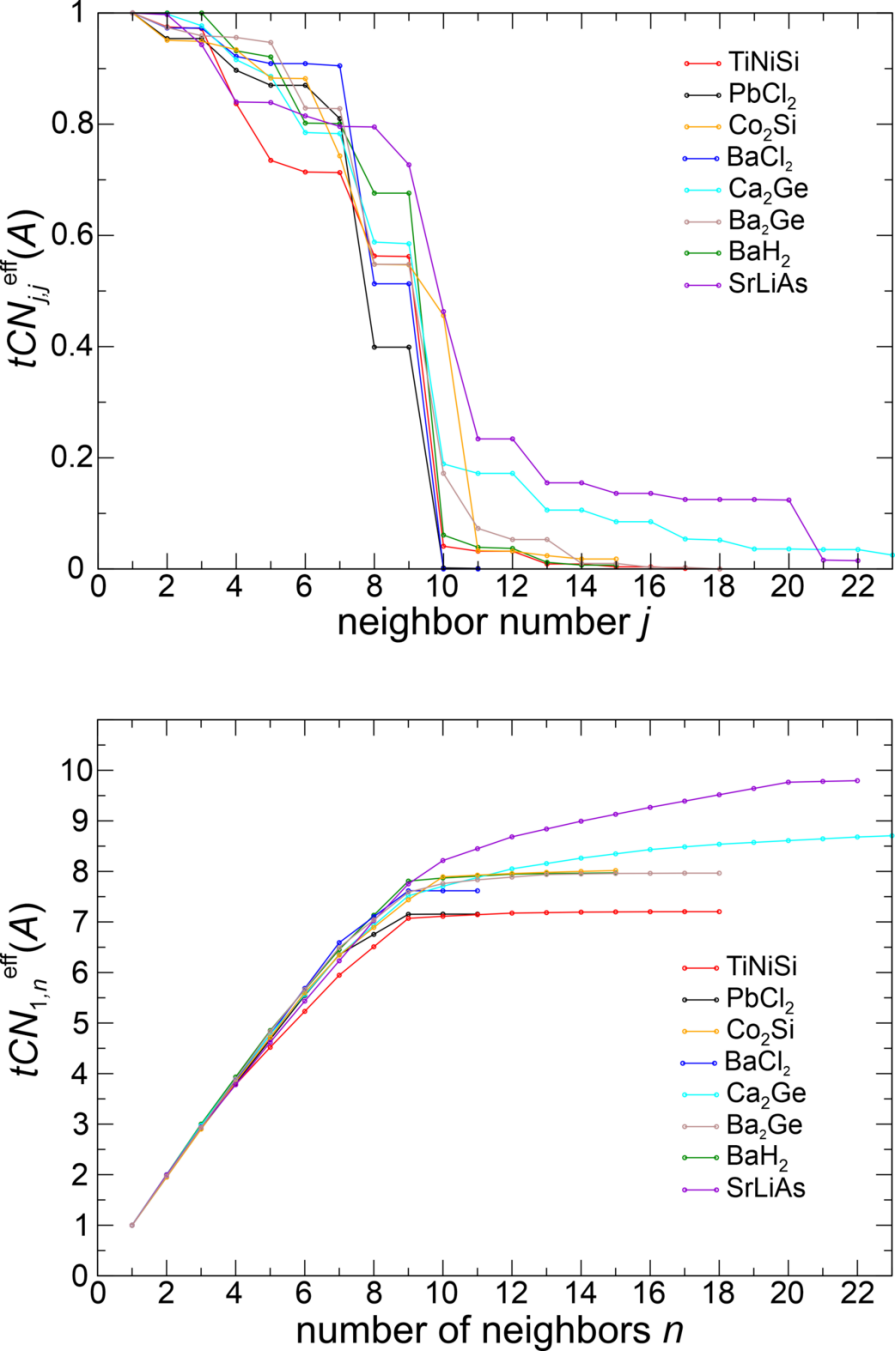
TiNiSi-type compounds. (Top) *j*-Dependent decay of 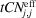
(‘*Si*’), (bottom) *n*-dependent rise of 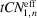
(‘*Si*’) for selected compounds.

Given the central role of the ‘*Si*’ species in this structure type, the trend of decreasing effective coordination increments (Fig. 10 ▸, top) leading to saturation of increasing effective coordination sum of the ‘*Si*’ type species was analyzed (Fig. 10 ▸, bottom). The low symmetry of the atomic sites (one mirror symmetry element) results in maximally two identical coordination increments. The jumps in effective coordination increments shown correspond to the linear gap values calculated with equation (11*c*[Disp-formula fd11c]) (scheme ‘lin’). It can be seen that the jump at *tCN*_*j*_(‘*Si*’) = 9 (*j* = 9) is strongest for TiNiSi, BaCl_2_ (Ba = ‘*Si*’), BaH_2_ (Ba = ‘*Si*’) and PbCl_2_ (Pb = ‘*Si*’) (see below). Other compounds still show a relevant increase even beyond the value at the dominant scenario ‘max’, which is mainly (except the Co_2_Si case) the result of ‘*Si*’–‘*Si*’ coordination.

*TiNiSi case*. There is always the canonical coordination scenario, which contains all significant faces of the three species, but displays, in all TiNiSi-type cases investigated, a weight clearly smaller than the dominant one. This scenario displays for Si *tCN*_tot_(Si) = 14 neighbors (with setting 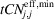 = 0.005, 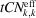 ≥ 0.005). The scenario 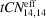
(Si) value defines a Si^[5Ti, 5Ni; 4Si]^ coordination environment, which directly yields the coordination scenario {Ti^[6Ni, 5Si; 0Ti]^Ni^[6Ti, 5Si; 2Ni]^ Si^[5Ti, 5Ni; 4Si]^} (Fig. 9 ▸). The corresponding normalized weights {^sc2^*W*, ^lin^*W*, ^sc1^*W*}° = {0.02, 0.06, 0.07}° are very small (Table 3 ▸, case *c*).

In Fig. 9 ▸ the coordination string reveals that the leading gap weight scenario occurs at *tCN*_max_(Si) = 9 and displays a purely heteroatomic Si^[5Ti, 4Ni; 0Si]^ environment. It is consistent with the largest gap in the Ti coordination sequence found at *tCN*_max_(Ti) = 5 with a Ti^[0Ni, 5Si; 0Ti]^ coordination and with the gapped Ni^[0Ti, 4Si; 0Ni]^ coordination *tCN*_max_(Ni) = 4. This constitutes the valid coordination scenario {Ti^[0Ni, 5Si; 0Ti]^Ni^[0Ti, 4Si; 0Ni]^ Si^[5Ti, 4Ni; 0Si]^} (Table 3 ▸, case *b*) with normalized scenario weights {^sc2^*W*, ^lin^*W*, ^sc1^*W*}° = {1, 1, 1}°. The normalized scenario weights ^*scheme*^*W*° = {0.59, 0.51, 0.40}° associated with *tCN*_7_(Si) coordination scenario (*a*) with a Si^[3Ti, 4Ni; 0Si]^ coordination environment are about half the size. In summary, with the canonical scenario (*c*) with very low weight, the leading sub-coordination scenario (*b*) was identified, which contains only heteroatomic *T*–Si (*T* = transition metals Ti, Ni) coordination.

*Co_2_Si case*. For Co_2_Si the following significant (sub-)coordination scenarios can be found:

Case (*b*) (Table 3 ▸) with *tCN*(Si) = 7 with environments {Co1^[0, 3; 0]^ Co2^[0, 4; 0]^ Si^[3, 4;0]^} is the most probable one for weighting schemes ‘sc2’ and ‘lin’. With weighting scheme ‘sc2’ the next most important one is located at even lower *tCN*(Si) = 6 environments {Co1^[0, 2; 0]^ Co2^[0, 4; 0]^ Si^[2, 4;0]^} (case *a*). For weighting scheme ‘sc1’ the *tCN*(Si) = 10 scenario {Co1^[6, 5; 2]^ Co2^[6, 5, 2]^ Si^[5, 5;0]^} (case *e*) at the high-coordination end is the most important one, and for scheme ‘lin’ it is almost equally important as case (*b*). Scenario cases (*d*) and (*e*) display quite similar weights and are located in-between the (*b*) and (*e*) cases.

It is remarkable that the *tCN*(Si) = 6 (2‘*Ti*’ + 4‘*Ni*’) scenario has no exact counterpart in the other compounds investigated (PbCl_2_ has 3‘*Ti*’ + 3‘*Ni*’; BaH_2_ has 5 = 1‘*Ti*’ + 4‘*Ni*’, see below). In comparison with the TiNiSi case, the normalized scenario weights are clearly smaller, and the scenarios are less clearly distinguished by large weight differences. In other words, the different scenarios are more smeared out. In contrast to TiNiSi, besides *T*–Si (*T* = transition metal) *T*–*T* coordination is always additionally present in all sub-coordination scenarios with Si coordination equal to or larger than 9. The dominating (schemes ‘sc2’ and ’lin’) *tCN*(Si) = 7 scenario with only heteroatomic contacts *T*–Si is similar to the PbCl_2_ case (Pb = ‘*Si*’). Moreover, in Co_2_Si and PbCl_2_ the *tCN*(‘*Si*’) = 9 scenario unavoidably features homoatomic coordination (Fig. 9 ▸). The *tCN*(‘*Si*’) = 10 scenario of Co_2_Si is not possible for PbCl_2_ (Fig. 9 ▸).

*SrLiAs case*. It is interesting to compare the case of SrLiAs with Co_2_Si, because both Co_2_Si and SrLiAs have been found to display ^BS^*CN*(‘*Si*’) = 10 in a BS type of coordination analysis (Freccero *et al.*, 2023 ▸).

In SrLiAs a special situation is found, which makes it slightly different from the other TiNiSi-type cases analyzed. Two dominant sub-coordination scenarios with ‘*Si*’ = As 9-coordination (Table 3 ▸, case *a*), like Si in TiNiSi, and with As 10-coordination (case *b*), with only slightly different normalized weights are found. Both of them exclusively display heteroatomic *M*–*E* coordination. Their relative importance is determined by the weighting scheme type. In all the other TiNiSi-type examples with dominating *tCN*(‘*Si*’) = 9 scenario, the preference is much more pronounced. The SrLiAs case is also clearly different from the Co_2_Si case. In the ‘*Si*’ 10-coordination scenario SrLiAs displays only heteroatomic coordination {Sr^[0, 6; 0]^ Li^[0, 4; 0]^ As^[6,4; 0]^} unlike the Co_2_Si case with environments {Co1^[3, 5; 2]^ Co2^[3, 5; 0]^ Si^[5, 5;0]^} and higher. Another difference with respect to Co_2_Si concerns the ligand composition of the 10-coordinated ‘*Si*’ species. For Co_2_Si it is a 5‘*Ti*’ + 5‘*Ni*’ coordination, for SrLiAs it is a 6‘*Ti*’ + 4‘*Ni*’ one. For SrLiAs as the example with the largest size difference of the TiNiSi-type compounds investigated, the effective coordination increments 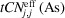 (Fig. 10 ▸) after the dominating gap are still significant and much larger than in TiNiSi or BaH_2_. As can be readily seen in Fig. 9 ▸, these contributions exclusively originate from homoatomic As neighbors.

Another systematic behavior is found for certain compounds considered binary variants of the TiNiSi type of structure: dominating scenarios gradually change from PbCl_2_ [*tCN*_max_(‘*Si*’) = 7], BaCl_2_ [*tCN*_max_(‘*Si*’) = 7], Ba_2_Ge [*tCN*_max_(‘*Si*’) = 7, 9], Ca_2_Ge[*tCN*_max_(‘*Si*’) = 7, 9] and finally to BaH_2_ [*tCN*_max_(‘*Si*’) = 9] corresponding to the situation in TiNiSi.

*PbCl_2_ case*. In the case of PbCl_2_ (Pb = ‘*Si*’) the gapped coordination assignment *tCN*_max_(Pb) = 7 is consistent with gapped *tCN*_max_(Cl1) = 3 and *tCN*_max_(Cl2) = 4 environments of the coordination scenario {Pb^[3, 4; 0]^ Cl1^[3, 0; 0]^} Cl2^[4, 0; 0]^} (Table 3 ▸, case *b*). Thus, this scenario forms an example of a dominating lower tCN at the ‘*Si*’ site of the TiNiSi type of structure. Investigation of the *tCN*(Pb) = 9 scenarios (cases *c*–*f*) yields much lower weights and a different coordination character, because it inevitably includes not only heteroatomic Pb–Cl coordination but also homoatomic Cl–Cl coordination, *e.g.* {Pb^[5, 4; 0]^ Cl1^[5, 4; 0]^} Cl2^[4, 4; 0]^}, and higher. In other words, for this compound a *tCN*(Pb) = 9 scenario with exclusively heteroatomic coordination does not exist (like in Co_2_Si, Fig. 9 ▸).

*BaCl_2_ case*. The next case to analyze is BaCl_2_ (Ba = ‘*Si*’). The possibility of finding a dominant (schemes ‘sc2’, ’lin’) sub-coordination scenario at *tCN*_max_(Ba) = 7 with only Ba–Cl contacts (Table 3 ▸, case *a*) relates it to PbCl_2_, but without having associated a lone pair of electrons at the cation. Like in PbCl_2_, a scenario with *tCN*(Ba) = 9 and only heteroatomic coordination of all species does not exist (cases *b*–*d*). These *tCN*(Ba) = 9 scenarios always contain Cl–Cl coordination, which is numerically (not necessarily similar stereochemical configuration) similar to Co–Co coordination in Co_2_Si. They display clearly lower scenario weights with schemes ‘sc2’ and ‘lin’, but case (*c*) represents the *tCN*_max_(Ba) = 9 scenario for the ‘sc1’ type of weighting.

*Cases Ba_2_Ge, Ca_2_Ge and BaH_2_*. The Ba_2_Ge case (Ge = ‘*Si*’) marks a transition from dominating *tCN*_max_(‘*Si*’) = 9 coordination scenario {Ba1^[0, 5; 0]^ Ba2^[0, 4; 0]^ Ge^[5, 4; 0]^} to the next most important *tCN*_max–1_(‘*Si*’) = 7 one {Ba1^[0, 3; 0]^ Ba2^[0, 4; 0]^Ge^[3, 4; 0]^}, where it is located roughly in the middle between the two, the exact extent being determined by the weighting scheme (Table 3 ▸, cases *a*, *b*). The compound Ca_2_Ge still displays a relevant *tCN*(‘*Si*’) = 7 coordination scenario but is clearly dominated by the *tCN*_max_(‘*Si*’) = 9 scenario {Ca1^[0, 5; 0]^ Ca2^[0, 4; 0]^ Ge^[5, 4; 0]^}.

The case of BaH_2_ (Ba = ‘*Si*’) is even more similar to the TiNiSi one, because it displays a similarly dominant sub-coordination scenario at *tCN*_max_(‘*Si*’) = 9 (case *c*) which clearly dominates over the *tCN*(‘*Si*’) = 7 (case *a*) one. For all sub-coordination scenarios up to *tCN*_max_(‘*Si*’) = 9 only heteroatomic coordination is found. It is interesting that for this compound an unusual *tCN*(‘*Si*’) = 5 sub-coordination scenario is found with a topological weight quite similar to the more usual *tCN*(‘*Si*’) = 7 one.

## 5. Related literature

The following references are cited in the supporting information: Chen *et al.* (2004 ▸), Wagner & Grin (2024 ▸).

## 6. Summary and conclusions

A generalization of the VDP method in terms of ED (QTAIM) atomic domains has been achieved. Solid angles subtended at the nuclear positions by the faces of ED (QTAIM) atomic domains have been computed for the first time and employed for definition of topological coordination numbers tCNs.

Coordination reciprocity (CR) has been implemented at two stages of the process. In the first instance, the combined solid angle of each IA contact has been used, which is not necessary in ^VDP^*CN* applications. In the second instance, sub-coordination scenarios consistent with CR have been defined and their relative importance has been weighted. This step had to be done in ^VDP^*CN* applications based on local coordination gaps as well, if CR-consistent sub-coordination scenarios were of interest. Sub-coordination scenario diagrams have been introduced to visualize these relations in general. CR plays an important role in the TiNiSi type of structures, where heteroatomic and homoatomic coordination compete (similar solid angles). For some compounds this happens only in less dominant sub-coordination scenarios, but for Co_2_Si this competition is clearly visible, resulting in a number of significant scenarios with comparable weights.

Three different weighting schemes have been tested, two semicircle-based ones (‘sc1’, ‘sc2’) and a linear one (‘lin’). They represent the prioritized weighting schemes for future studies. This is the first reported use of the ‘sc2’ weighting scheme. It leads to a favorable downsizing of scenario weight contributions of neighbors connected by comparably small solid angles.

The number of faces that a QTAIM atomic domain of type *A* shares with its neighbor atoms yields the total topological coordination number *tCN*_tot_(*A*) (an integer value); summation of the neighbors’ contributions normalized with respect to the largest solid angle of all contacts yields the effective coordination number 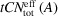, where 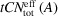 ≤ 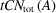 is valid.

The topological weight of a sub-coordination scenario represents a measure of the average coordination gap with respect to the subsequent sub-coordination scenario. As such, it quantifies how well this scenario is separated from the following one with more contacts included, which characterizes its importance as a well definable partial structure. The sub-coordination scenario with the highest topological weight ^scheme^*W*° is characterized by a set of species’ coordinations obeying CR; the number of contacts for each species in this scenario is given by 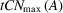, and the associated effective coordination number is 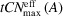 with 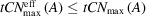 in complete analogy to the total CNs.

In general, in the tCN approach coordination may be considered to have two aspects: (i) the separability of well defined sub-coordination scenarios, and (ii) the inclusion of significant neighbors. While the first issue is monitored by the scenario weights, the second issue is monitored by the species’ *tCN*^eff^ values. The simultaneous fulfillment of both is not necessarily found for one partial structure. Already from this observation, listing of more than one (sub-)coordination scenario is typically necessary to characterize the topological coordination situation.

The tCN methodology is conceptually located along a holistic description of ED domain topological interconnections by employment of a set of relevant sub-coordination scenarios with high weights and their associated *tCN*_tot_(*A*), 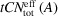 and *tCN*_max_(*A*), 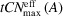, *tCN*_max–1_(*A*), 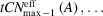 values. This kind of approach is considered to deliver more appropriate information for complex structural characterization than one that is based on just one integral coordination number per species.

Systematic series of 1-species (elemental) structures, 2-species structures and the TiNiSi-type of 3-species structure have been analyzed in the tCN framework and compared with VDP results from the literature (1- and 2-species structures only). In the cases compared, the tCN method yields smaller 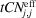 contributions of the higher (2nn) neighbors than the VDP method, which is explained by the ED decay naturally included in the tCN method.

The numerical results can be summarized as follows:

(*a*) For the elemental structures, a dominant 12-coordination for the *fcc* and *hcp* structures is found. For *bcc*, the 8-coordination scenario is found to clearly dominate over the 14-coordination one. The diamond structure strongly favors 4-coordination, and the 2nn neighbors display much smaller contributions.

(*b*) For 2-species structures, the size difference between the species becomes important, which is an effect completely absent in VDP-based methods. In the rocksalt-type ionic compounds investigated, the anions display the larger domain volumes in all cases except in RbF. The smaller species display only heteroatomic 6-coordination, the larger species a 6 + 12 coordination caused by additional homoatomic contact surfaces. LiI, with the largest size difference, represents the only case where even dominant 12-coordination of the large iodine species is obtained. In all other cases, the heteroatomic 6-coordination dominates for both species. In CsCl-type structures, mainly heteroatomic 8-coordination dominates, but a tendency to 14-coordination for the larger species (anions in the examples) is indicated. In zincblende-type structures, the contribution of the 12 2nn neighbors is larger than in the elemental structures, but still rather small, such that the four heteroatomic contacts represent the dominant scenario.

(*c*) For the TiNiSi type of 3-species structures analyzed, the analysis indicates a wider range of coordination scenarios, even when focusing only on the ‘*Si*’-type species displaying the highest effective CN in all structures and most dominant (sub)-coordination scenarios analyzed. It displays a dominant 7-coordination (PbCl_2_), 9-coordination (TiNiSi) and 10-coordination (SrLiAs, depending on weighting scheme).

Experiences gained in the tCN framework may be useful for further refinement of VDP technology, which is still employed in data mining applications related to machine learning and artificial intelligence because of its numerical robustness and speed.

Usage of promolecular EDs could be a way to obtain faster approximate results closer to the current tCN results than the VDP method. Of course, the price to pay for an approximate method is loss of accuracy compared with usage of ‘relaxed EDs’, such that one should consider whether the expected increase of precision compared with the VDP method will compensate for this disadvantage. In the case of accuracy problems of this approach, usage of improved non-spherical atomic densities in the framework of the Transferable Aspherical Atomic Model (TAAM) would be possible. This way, systematic investigation of huge data sets in data banks could be envisaged.

Usage of location of *cp*s as a secondary entity for further characterization of the contacts is considered important for future development of the ED-based tCN framework.

Further geometrical characteristics of ED-based atomic domains may be obtained from inclusion of (i) a distance measure of the atomic nuclei to the IA surfaces, (ii) contact surface area, and (iii) contact surface aperture area, which are related to the average curvature of the IA surface.

Advancing beyond the geometrical approach, the atomic effective charges, obtained by integration of the ED inside the atomic domains, can be used. This will not only yield a quantitative definition of cationic and anionic species in the compound, but also the electrostatic energy between atomic domains which is an important term in the exact IQA (interacting quantum atoms) type of decomposition of the total energy of the system into mono- and diatomic contributions. Experimental reconstruction of the pair density based interaction energies (related to covalent bonding) is ultimately challenging; approximations on the basis of ‘wavefunctions consistent with experimental X-ray scattering data’ and 1-matrix reconstruction techniques (Genoni, 2024 ▸) represent topics and directions of current development in the field of quantum crystallography (Macchi, 2022 ▸; Matta *et al.*, 2023 ▸). In general, these diatomic IQA energy contributions could then be considered as the energetic counterparts of the solid-angle values ω(*A*, *B*) forming the basis to set up coordination energy gap weights and effective coordination strengths.

## Supplementary Material

Supporting information. DOI: 10.1107/S2053273325002347/pen5011sup1.pdf

## Figures and Tables

**Figure 11:**
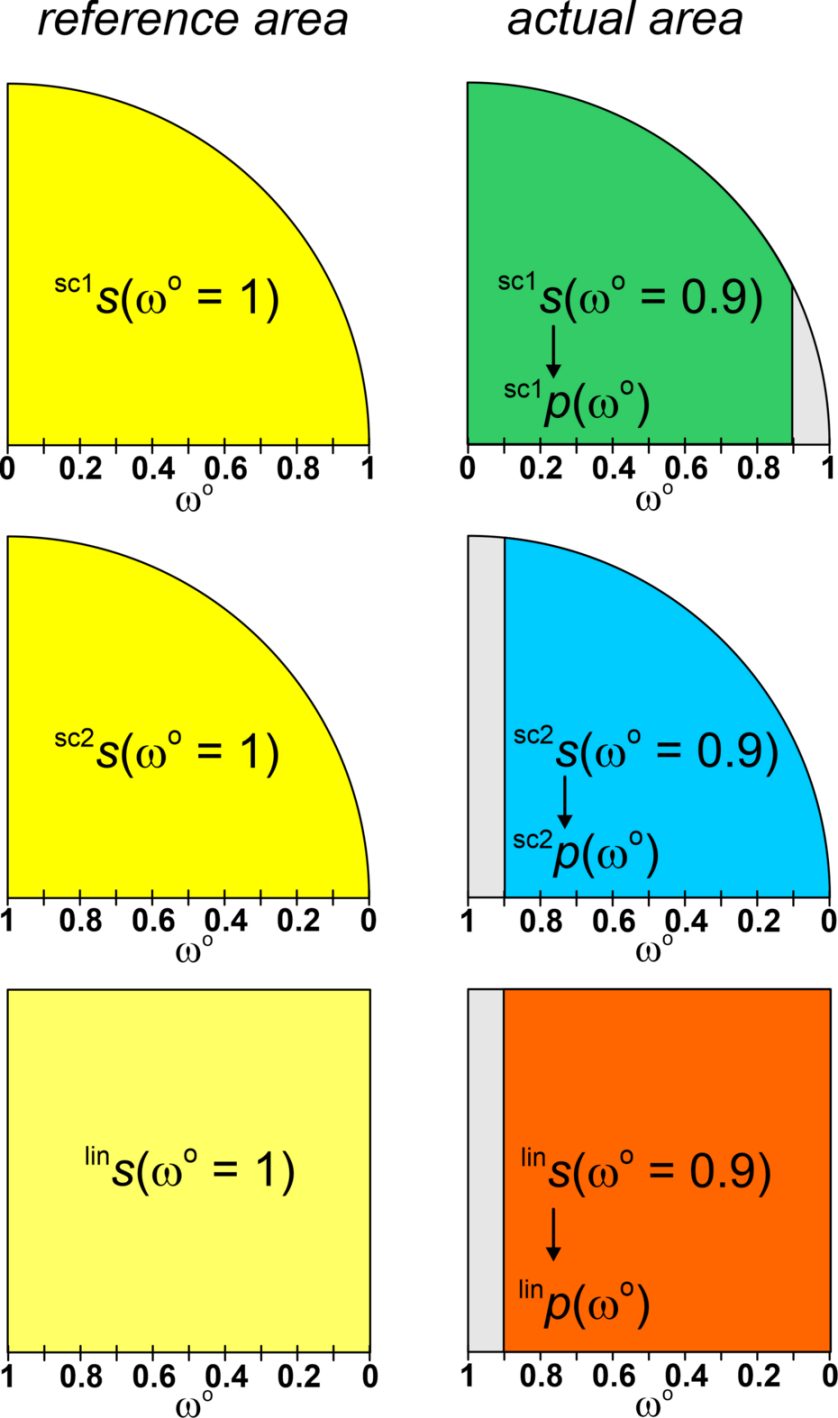
Weighting schemes for quantification of coordination gaps: semicircle-weighting schemes ‘sc1’ [top; equations (15), (16), (18)], ‘sc2’ [middle; equations (15), (17), (18)] and linear weighting scheme ‘lin’ [bottom; equations (19)–(21)] obtained from area calculation. Only half of the semicircles is shown. The principal difference between semicircle and linear weighting is the additional dependence of the coordination gap weights ^*scheme*^*p*(ω°) – ^*scheme*^*p*(ω°+Δω°) for a given Δω° on the position ω° in the ‘sc1’ and ‘sc2’ cases.

**Figure 12:**
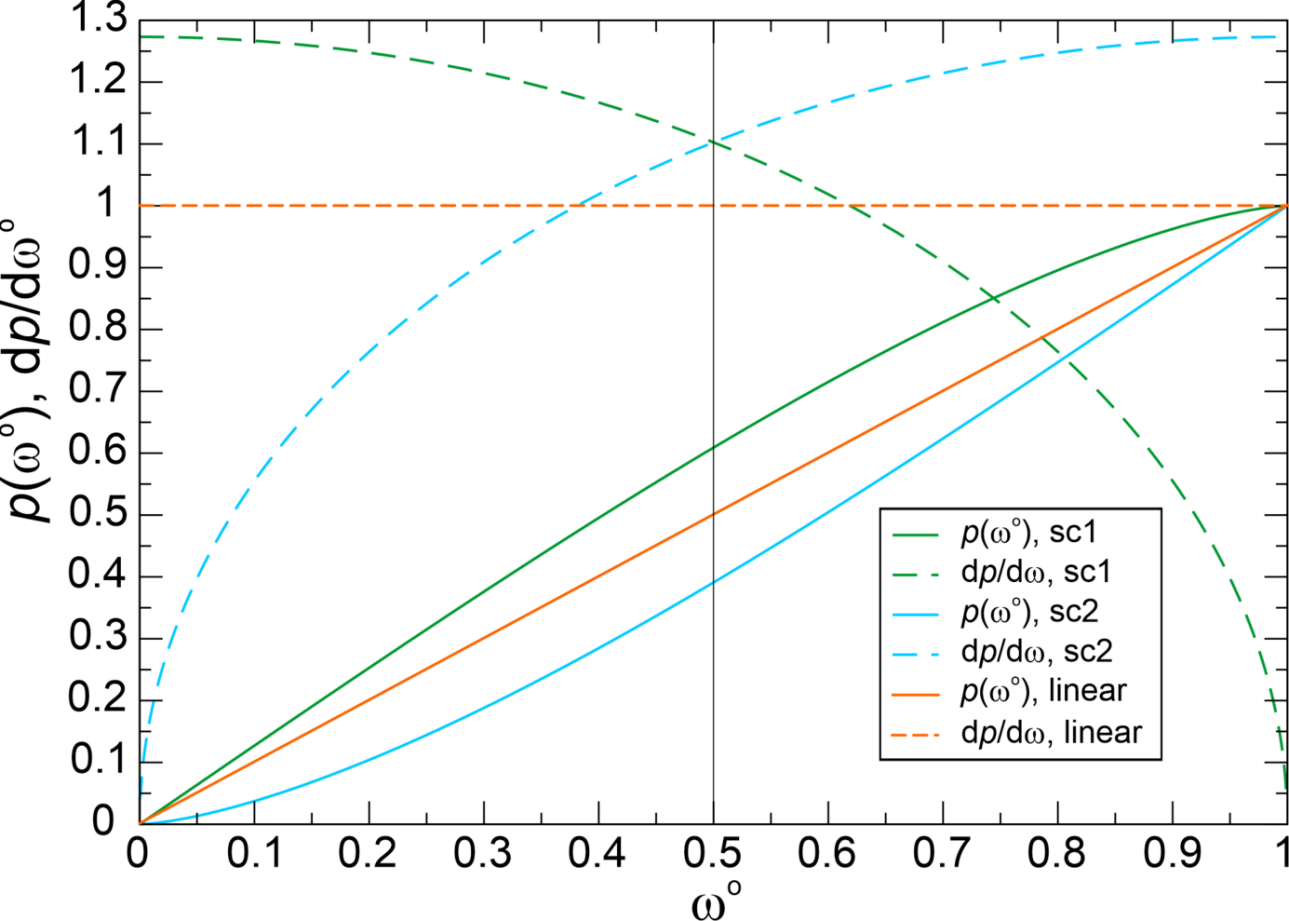
Functional behavior of semicircle ‘sc1’ (green) and ‘sc2’ (blue), and linear (’lin’) weighting schemes (orange).

**Table 1:** 1-Species element structures For each compound/structure different (sub-)coordination scenarios ‘*scene*’ (*a*), (*b*), … are listed in sequence of increasing 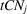
(*species1*); normalized scenario weights {^sc2^*W*, ^lin^*W*, ^sc1^*W*}° indicate the dominant scenario (value **1** for the respective weighting scheme, denoted ‘max’ in the text). The last scenario given always corresponds to inclusion of all domain surfaces (denoted ‘tot’ in the text).

Compound	Species1	Scenario
*scene*		weights {^*scheme*^*W*}°
*Fcc*-*M* = Ca, Rh, Pd	*tCN*_12_(*M*) 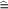 [12*M*]	{*M*^[12]^}
	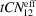 = 12	{**1, 1, 1**}°
		
*Bcc*-K	K	
(*a*)	*tCN*_8_(K) 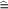 [8K]	{K^[8]^}
	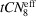 = 8	{**1, 1, 1**}°
(*b*)	*tCN*_14_(K) 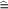 [14K]	{K^[14]^}
	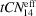 = 9.22	{0.12, 0.26, 0.35}°
		
*Bcc*-Mo	Mo	
(*a*)	*tCN*_8_(Mo) 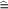 [8Mo]	{Mo^[8]^}
	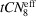 = 8	{**1, 1, 1**}°
(*b*)	*tCN*_14_(Mo) 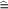 [14Mo]	{Mo^[14]^}
	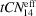 = 9.18	{0.12, 0.25, 0.34}°
		
*Hcp*-Ti	Ti (*c*/*a* = 1.588)	
(*a*)	*tCN*_6_(Ti) 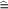 [6Ti]	{Ti^[6]^}
	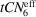 = 6	{0.16, 0.12, 0.05}°
(*b*)	*tCN*_12_(Ti) 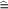 [12Ti]	{Ti^[12]^}
	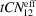 = 11.27	{**1, 1, 1**}°
		
*Hcp*-Mg	Mg (*c*/*a* = 1.623)	
(*a*)	*tCN*_6_(Mg) 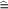 [6Mg]	{tCN_6_(Mg)}, {Mg^[6]^}
	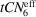 = 6	{0.30, 0.22, 0.10}°
(*b*)	*tCN*_12_(Mg) 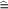 [12Mg]	{Mg^[12]^}
	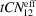 = 10.85	{**1, 1, 1**}°
		
*Hcp*-Zn	Zn (*c*/*a* = 1.861)	
(*a*)	*tCN*_6_(Zn) 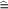 [6Zn]	{Zn^[6]^}
	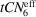 = 6	{0.82, 0.56, 0.32}°
(*b*)	*tCN*_12_(Zn) 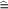 [12Zn]	{Zn^[12]^}
	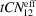 = 9.79	{**1, 1, 1**}°
		
Diamond		
C	C	
(*a*)	*tCN*_4_(C) 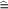 [4C]	{C^[4]^}
	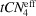 = 4	{**1, 1, 1**}°
(*b*)	*tCN*_16_(C) 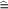 [16C]	{C^[16]^}
	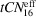 = 4.07	{0.00, 0.01, 0,01}°
		
Si	Si	
(*a*)	*tCN*_4_(Si) 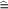 [4Si]	{Si^[4]^}
	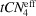 = 4	{**1, 1, 1**}°
(*b*)	*tCN*_16_(Si) 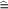 [16Si]	{Si^[16]^}
	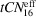 = 4.05	{0.00, 0.00 0.01}°
		
Ge	Ge	
(*a*)	*tCN*_4_(Ge) 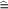 [4Ge]	{Ge^[4]^}
	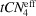 = 4	{**1, 1, 1**}°
(*b*)	*tCN*_16_(Ge) 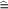 [16Ge]	{Ge^[16]^}
	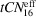 = 4.07	{0.00, 0.01 0.01}°

**Table 2:** Binary 2-species structures Atomic volumes *V* are given for each species. Different (sub-)coordination scenarios ‘*scene*’ (*a*), (*b*), … are listed with increasing 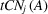 of at least one species *A*; normalized scenario weights {^sc2^*W*, ^lin^*W*, ^sc1^*W*}° indicate the dominant scenario (value **1** for the respective weighting scheme, denoted ‘max’ in the text). The last scenario given always corresponds to inclusion of all domain surfaces (denoted ‘tot’ in the text).

Compound	Species1	Species2	Scenario
*scene*			weights {^*scheme*^*W*}°
Zincblende type			
BN	B (*V* = 1.66 Å^3^)	N (*V* = 10.3 Å^3^)	
(*a*)	*tCN*_4_(B) 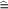 [4N; 0]	*tCN*_4_(N) 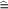 [4B; 0]	{B^[4; 0]^ N^[4; 0]^}
	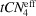 = 4	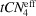 = 4	{**1, 1, 1**}°
(*b*)	*tCN*_4_(B) 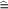 [4N; 0]	*tCN*_16_(N) 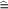 [4B; 12N]	{B^[4; 0]^ N^[4; 12]^}
	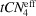 = 4	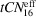 = 5.52	{0.24, 0.39, 0.45}°
			
BP	B (*V* = 6.97 Å^3^)	P (*V* = 16.6 Å^3^)	
(*a*)	*tCN*_4_(B) 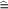 [4P; 0]	*tCN*_4_(P) 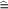 [4B; 0]	{B^[4; 0]^ P^[4; 0]^}
	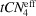 = 4	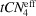 = 4	{**1, 1, 1**}°
(*b*)	*tCN*_4_(B) 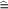 [4P; 0]	*tCN*_16_(P) 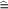 [4B; 12P]	{B^[4; 0]^ P^[4; 12]^}
	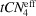 = 4	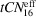 = 4.40	{0.09, 0.19, 0.22}°
			
GaN	Ga (*V* = 10.1 Å^3^)	N (*V* = 13.4 Å^3^)	
(*a*)	*tCN*_4_(Ga) 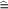 [4N; 0]	*tCN*_4_(N) 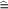 [4Ga; 0]	{Ga^[4; 0]^ N^[4; 0]^}
	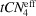 = 4	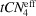 = 4	{**1, 1, 1**}°
(*b*)	*tCN*_4_(Ga) 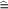 [4N; 0]	*tCN*_16_(N) 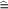 [4Ga; 12N]	{Ga^[4; 0]^ N^[4; 12]^}
	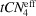 = 4	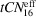 = 4.21	{0.06, 0.14, 0.16}°
			
GaP	Ga (*V* = 15.9 Å^3^)	P (*V* = 25.9 Å^3^)	
(*a*)	*tCN*_4_(Ga) 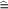 [4P; 0]	*tCN*_4_(P) 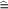 [4Ga; 0]	{Ga^[4; 0]^ P^[4; 0]^}
	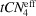 = 4	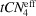 = 4	{**1, 1, 1**}°
(*b*)	*tCN*_4_(Ga) 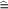 [4P; 0]	*tCN*_16_(P) 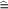 [4Ga; 12P]	{Ga^[4; 0]^ P^[4; 12]^}
	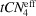 = 4	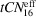 = 4.28	{0.07, 0.17, 0.19}°
			
GaAs	Ga (*V* = 18.2 Å^3^)	As (*V* = 29.3 Å^3^)	
(*a*)	*tCN*_4_(Ga) 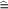 [4As; 0]	*tCN*_4_(As) 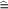 [4Ga; 0]	{Ga^[4; 0]^ As^[4; 0]^}
	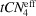 = 4	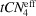 = 4	{**1, 1,1**}°
(*b*)	*tCN*_4_(Ga) 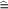 [4As; 0]	*tCN*_16_(As) 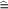 [4Ga; 12As]	{Ga^[4; 0]^ As^[4; 12]^}
	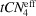 = 4	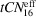 = 4.27	{0.07, 0.16, 0.18}°
			
GaSb	Ga (*V* = 22.5 Å^3^)	Sb (*V* = 37.5 Å^3^)	
(*a*)	*tCN*_4_(Ga) 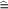 [4Sb; 0]	*tCN*_4_(Sb) 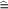 [4Ga; 0]	{Ga^[4; 0]^ Sb^[4; 0]^}
	*tCN*_4_^eff^ = 4	*tCN*_4_^eff^ = 4	{**1, 1, 1**}°
(*b*)	tCN_4_(Ga) 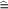 [4Sb; 0]	tCN_16_(Sb) 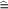 [4Ga; 12Sb]	{Ga^[4; 0]^ Sb^[4; 12]^}
	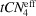 = 4	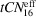 = 4.32	{0.079, 0.18, 0.20}°
			
Rocksalt type			
NaCl	Na (*V* = 9.72 Å^3^)	Cl (*V* = 36.5 Å^3^)	
(*a*)	*tCN*_6_(Na) 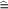 [6Cl; 0]	*tCN*_6_(Cl) 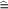 [6Na; 0]	{Na^[6; 0]^ Cl^[6; 0]^}
	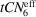 = 6	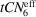 = 6	{**1, 1, 1**}°
(*b*)	*tCN*_6_(Na) 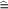 [6Cl; 0]	*tCN*_18_(Cl) 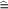 [6Na; 12Cl]	{Na^[6; 0]^ Cl^[6; 12]^}
	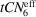 = 6	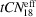 = 10.48	{0.59, 0.78, 0.95}°
			
LiI	Li (*V* = 4.50 Å^3^)	I (*V* = 49.7 Å^3^)	
(*a*)	*tCN*_6_(Li) 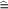 [6I; 0]	*tCN*_6_(I) 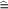 [6Li; 0]	{Li^[6; 0]^, I^[6; 0]^}
	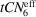 = 6	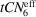 = 6	{0.82, 0.70, 0.52}°
(*b*)	*tCN*_6_(Li) 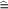 [6I; 0]	*tCN*_18_(I) 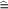 [6Li; 12I]	{Li^[6; 0]^, I^[6; 12]^}
	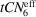 = 6	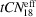 = 14.06	{**1, 1, 1**}°
			
KI	K (*V* = 24.4 Å^3^)	I (*V* = 67.4 Å^3^)	
(*a*)	*tCN*_6_(K) 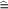 [6I; 0]	*tCN*_6_(I) 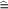 [6K; 0]	{K^[6; 0]^, I^[6; 0]^}
	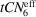 = 6	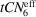 = 6	{**1, 1, 1**}°
(*b*)	*tCN*_6_(K) 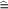 [6I; 0]	*tCN*_18_(I) 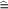 [6K; 12I]	{Li^[6; 0]^, I^[6; 12]^}
	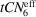 = 6	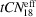 = 9.34	{0.45, 0.62, 0.73}°
			
RbI	Rb (*V* = 32.9 Å^3^)	I (*V* = 70.7 Å^3^)	
(*a*)	*tCN*_6_(Rb) 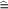 [6Cl; 0]	*tCN*_6_(I) 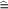 [6Rb; 0]	{Rb^[6; 0]^, I^[6; 0]^}
	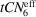 = 6	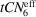 = 6	{**1, 1, 1**}°
(*b*)	*tCN*_6_(Rb) 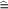 [6Cl; 0]	*tCN*_18_(I) 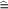 [6Rb; 12I]	{Li^[6; 0]^, I^[6; 12]^}
	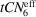 = 6	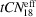 = 8.28	{0.34, 0.49, 0.56}°
			
RbF	Rb (*V* = 27.7 Å^3^)	F (*V* = 19.5 Å^3^)	
(*a*)	*tCN*_6_(Rb) 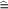 [6F; 0]	*tCN*_6_(F) 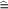 [6Rb; 0]	{Rb^[6; 0]^, F^[6; 0]^}
	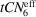 = 6	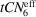 = 6	{**1, 1, 1**}°
(*b*)	*tCN*_18_(Rb) 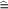 [6F; 12Rb]	*tCN*_6_(F) 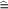 [6Rb; 0] tCN_6_^eff^	{Rb^[6; 12]^, F^[6; 0]^}
	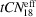 = 6.38	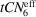 = 6	{0.10, 0.20, 0.23}°
			
CsCl type			
CsCl	Cs (*V* = 37.5 Å^3^)	Cl (*V* = 36.4 Å^3^)	
(*a*)	*tCN*_8_(Cs) 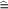 [8Cl; 0]	*tCN*_8_(Cl) 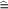 [8Cs; 0]	{Cs^[8; 0]^ Cl^[8; 0]^}
	*tCN*_8_^eff^ = 8	*tCN*_8_^eff^ = 8	{**1, 1, 1**}°
(*b*)	*tCN*_14_(Cs) 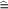 [8Cl; 6Cs]	*tCN*_8_(Cl) 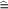 [8Cs; 0]	{Cs^[8; 6]^ Cl^[8; 0]^}
	*tCN*_14_^eff^ = 9.80	*tCN*_8_^eff^ = 8	{0.49, 0.67, 0.79}°
(*c*)	*tCN*_8_(Cs) 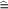 [8Cl; 0]	*tCN*_14_(Cl) 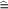 [8Cs; 6Cl]	{Cs^[8; 0]^ Cl^[8; 6]^}
	*tCN*_8_^eff^ = 8	*tCN*_14_^eff^ = 9.84	{0.50, 0.68, 0.80}°
(*d*)	*tCN*_14_(Cs) 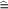 [8Cl; 6Cs]	*tCN*_14_(Cl) 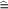 [8Cs; 6Cl]	{Cs^[8; 6]^ Cl^[8; 6^}
	*tCN*_14_^eff^ = 9.80	*tCN*_14_^eff^ = 9.84	{0.25, 0.45, 0.64}°
			
CsI	Cs (*V* = 42.1 Å^3^)	I (*V* = 58.1 Å^3^)	
(*a*)	*tCN*_8_(Cs) 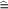 [8I; 0]	*tCN*_8_(I) 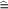 [8Cs; 0]	{Cs^[8; 0]^, I^[8; 0]^}
	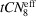 = 8	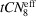 = 8	{**1**, 0.97, 0.78}°
(*b*)	*tCN*_14_(Cs) 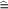 [8I; 6Cs]	*tCN*_8_(I) 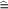 [8Cs; 0]	{Cs^[8; 6]^, I^[8; 0]^}
	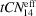 = 8.76	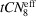 = 8	{0.23, 0.37, 0.35}°
(*c*)	*tCN*_8_(Cs) 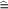 [8I; 0]	*tCN*_14_(I) 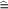 [8Cs; 6I]	{Cs^[8; 0]^, I^[8; 6]^}
	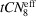 = 8	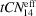 = 11.08	{0.83, **1**, **1**}°
(*d*)	*tCN*_14_(Cs) 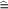 [8I; 6Cs]	*tCN*_14_(I) 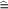 [8Cs; 6I]	{Cs^[8; 6]^, I^[8; 6]^}
	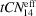 = 8.76	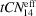 = 11.08	{0.19, 0.39, 0.43}°

**Table 3:** TiNiSi-type 3-species structures For each species *A* the QTAIM volume *V* is given. (Sub)-coordination scenarios are listed with increasing overall coordination. For each scenario ‘*scene*’ (*a*, *b*, …) and each species are listed: coordination notation, 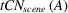 and 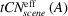; normalized scenario weights {^sc2^*W*, ^lin^*W*, ^sc1^*W* }° indicate the dominant scenario (value **1** for the respective weighting ‘*scheme*’, denoted ‘max’ in the text). The last scenario given always corresponds to inclusion of all domain surfaces (denoted ‘tot’ in the text).

Compound	Species ‘*Si*’	Species ‘*Ti*’	Species ‘*Ni*’	Scenario
*scene*				weights {^*scheme*^*W*}°
TiNiSi	Si (*V* = 15.6 Å^3^)	Ti (*V* = 10.4 Å^3^)	Ni (*V* = 13.4 Å^3^)	
(*a*)	*tCN*_7_(Si) 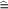 [3Ti, 4Ni; 0]	*tCN*_3_(Ti) 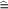 [0, 3Si; 0]	*tCN*_4_(Ni) 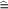 [0, 4Si; 0]	{Ti^[0, 3; 0]^ Ni^[0, 4; 0]^ Si^[3, 4; 0]^}
	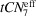 = 5.95	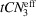 = 2.16	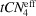 = 3.78	{0.59, 0.51, 0.40}°
(*b*)	*tCN*_9_(Si) 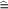 [5Ti, 4Ni; 0]	*tCN*_5_(Ti) 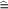 [0, 5Si; 0]	*tCN*_4_(Ni) 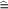 [0, 4Si; 0]	{Ti^[0, 5; 0]^ Ni^[0, 4; 0]^ Si^[5, 4; 0]^}
	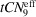 = 7.07	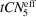 = 3.29	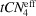 = 3.78	{**1, 1, 1**}°
(*c*)	*tCN*_14_(Si) 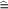 [5Ti, 5Ni; 4Si]	*tCN*_11_(Ti) 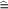 [6Ni, 5Si; 0]	*tCN*_13_(Ni) 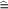 [6Ti, 5Si; 2Ni]	{Ti^[6, 5; 0]^ Ni^[6, 5; 2]^ Si^[5, 5; 4]^}
	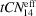 = 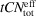 = 7.19	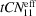 = 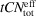 = 4.39	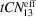 = 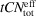 = 5.38	{0.02, 0.06, 0.07}°
				
Co_2_Si	Si (*V* = 10.6 Å^3^)	Co1 (*V* = 10.8 Å^3^)	Co2 (*V* = 11.1 Å^3^)	
(*a*)	*tCN*_6_(Si) 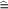 [2Co1, 4Co2; 0]	*tCN*_2_(Co1) 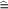 [0, 2Si; 0]	*tCN*_4_(Co2) 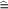 [0, 4Si; 0]	{Co1^[0, 2; 0]^ Co2^[0, 4; 0]^ Si^[2, 4;0]^}
	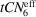 = 5.60	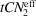 = 1.77	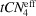 = 3.83	{0.82, 0.80, 0.44}°
(*b*)	*tCN*_7_(Si) 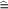 [3Co1, 4Co2; 0]	*tCN*_3_(Co1) 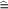 [0, 3Si; 0]	*tCN*_4_(Co2) 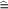 [0, 4Si; 0]	{Co1^[0, 3; 0]^ Co2^[0, 4; 0]^ Si^[3, 4;0]^}
	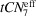 = 6.34	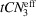 = 2.51	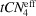 = 3.83	{**1, 1**, 0.73}°
(*c*)	*tCN*_9_(Si) 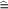 [5Co1, 4Co2; 0]	*tCN*_7_(Co1) 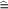 [0, 5Si; 2Co1]	*tCN*_4_(Co2) 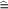 [0, 4Si; 0]	{Co1^[0, 5; 2]^ Co2^[0, 4’ 0]^ Si^[5, 4;0]^}
	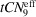 = 7.44	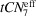 = 4.69	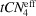 = 3.83	{0.52, 0.55, 0.45}°
(*d*)	*tCN*_10_(Si) 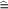 [5Co1, 5Co2; 0]	*tCN*_10_(Co1) 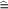 [3Co2, 5Si; 2Co1]	*tCN*_8_(Co2) 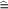 [3Co1, 5Si; 0]	{Co1^[3, 5; 2]^ Co2^[3, 5; 0]^ Si^[5, 5;0]^}
	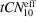 = 7.89	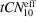 = 6.02	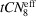 = 5.62	{0.48, 0.62, 0.60}°
(*e*)	*tCN*_10_(Si) 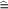 [5Co1, 5Co2; 0]	*tCN*_13_(Co1) 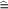 [6Co2, 5Si; 2Co1]	*tCN*_13_(Co2) 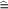 [6Co1, 5Si; 2Co2]	{Co1^[6, 5; 2]^ Co2^[6, 5, 2]^ Si^[5, 5;0]^}
	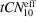 = 7.89	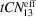 = 6.89	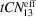 = 6.90	{0.51, 0.98, **1**}°
(*f*)	*tCN*_15_(Si) 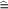 [6Co1, 5Co2; 4Si]	*tCN*_14_(Co1) 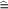 [6Co2, 6Si; 2Co1]	*tCN*_13_(Co2] 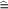 [6Co1, 5Si; 2Co2]	{Co1^[6, 6; 2]^ Co2^[6, 5; 2]^ Si^[6, 5;4]^}
	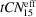 = 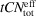 = 8.02	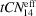 = 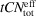 = 6.91	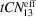 = 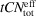 = 6.90	{0.03, 0.16, 0.17}°
				
SrLiAs	As (*V* = 45.0 Å^3^)	Sr (*V* = 21.2 Å^3^)	Li (*V* = 4.3 Å^3^)	
(*a*)	*tCN*_9_(As) 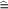 [5Sr, 4Li; 0]	*tCN*_5_(Sr) 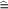 [0, 5As; 0]	*tCN*_4_(Li) 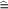 [0, 4As; 0]	{Sr^[0, 5; 0]^ Li^[0, 4; 0]^ As^[5,4; 0]^}
	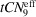 = 7.75	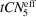 = 4.21	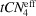 = 3.54	{**1**, 0.94, 0.82}°
(*b*)	*tCN*_10_(As) 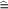 [6Sr, 4Li; 0]	*tCN*_6_(Sr) 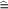 [0, 6As; 0]	*tCN*_4_(Li) 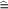 [0, 4As; 0]	{Sr^[0, 6; 0]^ Li^[0, 4; 0]^ As^[6,4; 0]^}
	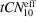 = 8.22	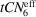 = 4.68	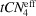 = 3.54	{0.87, **1, 1**}°
(*c*)	*tCN*_20_(As) 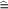 [6Sr, 4Li; 10As]	*tCN*_6_(Sr) 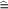 [0, 6As; 0]	*tCN*_4_(Li) 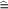 [0, 4As; 0]	{Sr^[0, 6; 0]^ Li^[0, 4; 0]^ As^[6,4; 10]^}
	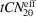 = 9.77	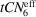 = 4.68	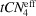 = 3.54	{0.53, 0.78, 0.80}°
(*d*)	*tCN*_22_(As) 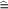 [6Sr, 4Li; 12As]	*tCN*_6_(Sr) 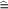 [0, 6As; 0]	*tCN*_4_(Li) 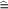 [0, 4As; 0]	{Sr^[0, 6; 0]^ Li^[0, 4; 0]^ As^[6,4; 12]^}
	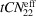 = 9.80	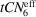 = 4.68	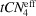 = 3.54	{0.19, 0.40, 0.41}°
				
PbCl_2_	Pb (*V* = 23.7 Å^3^)	Cl1 (*V* = 28.0 Å^3^)	Cl2 (*V* = 26.4 Å^3^)	
(*a*)	*tCN*_6_(Pb) 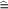 [3Cl1, 3Cl2; 0]	*tCN*_3_(Cl1) 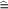 [3Pb, 0; 0]	*tCN*_3_(Cl2) 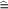 [3Pb, 0; 0]	{Pb^[3, 3; 0]^ Cl1^[3, 0; 0]^} Cl2^[3, 0; 0]^}
	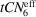 = 5.55	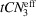 = 2.74	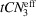 = 2.80	{0.35, 0.33, 0.25}°
(*b*)	*tCN*_7_(Pb) 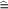 [3Cl1, 4Cl2; 0]	*tCN*_3_(Cl1) 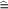 [3Pb, 0; 0]	*tCN*_4_(Cl2) 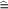 [4Pb, 0; 0]	{Pb^[3, 4; 0]^ Cl1^[3, 0; 0]^} Cl2^[4, 0; 0]^}
	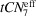 = 6.35	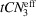 = 2.74	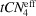 = 3.61	{**1, 1, 1**}°
(*c*)	*tCN*_9_(Pb) 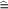 [5Cl1, 4Cl2; 0]	*tCN*_9_(Cl1) 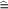 [5Pb, 4Cl2; 0]	*tCN*_8_(Cl2) 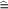 [4Pb, 4Cl1; 0]	{Pb^[5, 4; 0]^ Cl1^[5, 4; 0]^} Cl2^[4, 4; 0]^}
	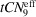 = 7.15	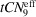 = 5.30	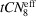 = 5.38	{0.21, 0.28, 0.35}°
(*d*)	*tCN*_9_(Pb) 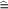 [5Cl1, 4Cl2; 0]	*tCN*_9_(Cl1) 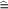 [5Pb, 4Cl2; 0]	*tCN*_10_(Cl2) 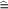 [4Pb, 4Cl1; 2Cl2]	{Pb^[5, 4; 0]^ Cl1^[5, 4; 0]^} Cl2^[4, 4; 2]^}
	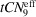 = 7.15	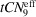 = 5.30	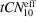 = 6.17	{0.32, 0.43, 0.54}°
(*e*)	*tCN*_9_(Pb) 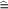 [5Cl1, 4Cl2; 0]	*tCN*_13_(Cl1) 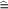 [5Pb, 6Cl2; 2Cl1]	*tCN*_12_(Cl2) 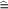 [4Pb, 6Cl1; 2Cl2]	{Pb^[5, 4; 0]^ Cl1^[5, 6; 2]^} Cl2^[4, 6; 2]^}
	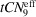 = 7.15	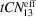 = 6.27	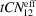 = 6.67	{0.28, 0.52, 0.68}°
(*f*)	*tCN*_9_(Pb) 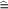 [5Cl1, 4Cl2; 0]	*tCN*_15_(Cl1) 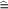 [5Pb, 6Cl2; 4Cl1]	*tCN*_12_(Cl2) 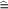 [4Pb, 6Cl1; 2Cl2]	{Pb^[5, 4; 0]^ Cl1^[5, 6; 4]^} Cl2^[4, 6; 2]^}
	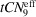 = 7.15	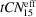 = 6.49	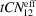 = 6.67	{0.28, 0.52, 0.68}°
				
BaCl_2_	Ba (*V* = 27.0 Å^3^)	Cl1 (*V* = 33.6 Å^3^)	Cl2 (*V* = 30.2 Å^3^)	
(*a*)	*tCN*_7_(Ba) 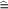 [3Cl1, 4Cl2; 0]	*tCN*_3_(Cl1) 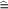 [3Ba, 0Cl2; 0]	*tCN*_4_(Cl2) 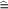 [4Ba, 0Cl1; 0]	{Ba^[3, 4; 0]^ Cl1^[3, 0; 0]^} Cl2^[4, 0; 0]^}
	[0.4733, 0.3916, 0.3418]	[0.5006, 0.4059, 0.3036]	[0.4496, 0.3704, 0.3186]	{0.47, 0.39, 0.32}
	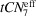 = 6.59	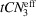 = 2.74	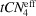 = 3.85	{**1, 1**, 0.83}°
(*b*)	*tCN*_9_(Ba) 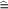 [5Cl1, 4Cl2; 0]	*tCN*_9_(Cl1) 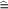 [5Ba, 4Cl2; 0]	*tCN*_10_(Cl2) 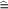 [4Ba, 4Cl1; 2Cl2]	{Ba^[5, 4; 0]^ Cl1^[5, 4; 0]^} Cl2^[4, 4; 2]^}
	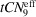 = 7.62	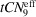 = 5.89	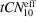 = 6.95	{0.41, 0.52, 0.61}°
(*c*)	*tCN*_9_(Ba) 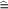 [5Cl1, 4Cl2; 0]	*tCN*_15_(Cl1) 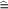 [5Ba, 6Cl2; 4Cl1]	*tCN*_12_(Cl2) 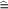 [4Ba, 6Cl1; 2Cl2]	{Ba^[5, 4; 0]^ Cl1^[5, 6; 4]^} Cl2^[4, 6; 2]^}
	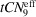 = 7.62	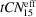 = 7.48	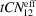 = 7.65	{0.43, 0.80, **1**}°
(*d*)	*tCN*_9_(Ba) 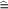 [5Cl1, 4Cl2; 0]	*tCN*_17_(Cl1) 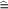 [5Ba, 6Cl2; 6Cl1]	*tCN*_12_(Cl2) 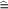 [4Ba, 6Cl1; 2Cl2]	{Ba^[5, 4; 0]^ Cl1^[5, 6; 6]^} Cl2^[4, 6; 2]^}
	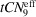 = 7.62	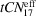 = 7.50	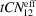 = 7.65	{0.09, 0.30, 0.37}°
				
Ba_2_Ge	Ge (*V* = 49.8 Å^3^)	Ba1 (*V* = 35.3 Å^3^)	Ba2 (*V* = 33.4 Å^3^)	
(*a*)	*tCN*_7_(Ge) 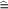 [3Ba1, 4Ba2; 0]	*tCN*_3_(Ba1) 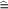 [0, 3Ge; 0]	*tCN*_4_(Ba2) 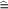 [0, 4Ge; 0]	{Ba1^[0, 3; 0]^ Ba2^[0, 4; 0]^ Ge^[3, 4; 0]^}
	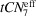 = 6.49	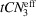 = 2.66	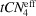 = 3.84	{0.98, 0.85, 0.71}°
(*b*)	*tCN*_9_(Ge) 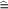 [5Ba1, 4Ba2; 0]	*tCN*_5_(Ba1) 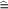 [0, 5Ge; 0]	*tCN*_4_(Ba2) 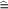 [0, 4Ge; 0]	{Ba1^[0, 5; 0]^ Ba2^[0, 4; 0]^ Ge^[5, 4; 0]^}
	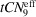 = 7.59	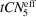 = 3.75	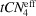 = 3.84	{**1, 1, 1**}°
(*c*)	*tCN*_15_(Ge) 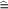 [6Ba1,5Ba2;4Ge]	*tCN*_14_(Ba1) 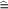 [6Ba2,6Ge;2Ba1]	*tCN*_13_(Ba2) 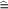 [6Ba1,5Ge;2Ba2]	{Ba1^[6, 6; 2]^ Ba2^[6, 5; 2]^ Ge^[6, 5; 4]^}
	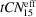 = 7.96	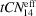 = 4.82	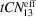 = 4.85	{0.004, 0.021, 0.027}°
				
Ca_2_Ge	Ge (*V* = 49.8 Å^3^)	Ca1 (*V* = 17.9 Å^3^)	Ca2 (*V* = 16.9 Å^3^)	
(*a*)	*tCN*_7_(Ge) 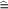 [3Ca1, 4Ca2; 0]	*tCN*_3_(Ca1) 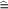 [0, 3Ge; 0]	*tCN*_4_(Ca2) 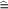 [0, 4Ge; 0]	{Ca1^[0, 3; 0]^ Ca2^[0, 4; 0]^ Ge^[3, 4; 0]^}
	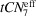 = 6.35	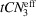 = 2.48]	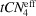 = 3.86	{0.71, 0.62, 0.52}°
(*b*)	*tCN*_9_(Ge) 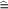 [5Ca1, 4Ca2; 0]	*tCN*_5_(Ca1) 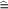 [0, 5Ge; 0]	*tCN*_4_(Ca2) 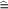 [0, 4Ge; 0]	{Ca1^[0, 5; 0]^ Ca2^[0, 4; 0]^ Ge^[5, 4; 0]^}
	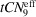 = 7.52	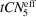 = 3.66	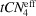 = 3.86	{**1, 1, 1**}°
(*c*)	*tCN*_23_(Ge) 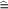 [6Ca1,5Ca2;12Ge]	*tCN*_8_(Ca1) 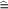 [2Ca2, 6Ge; 0]	*tCN*_7_(Ca2) 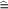 [2Ca1, 5Ge; 0]	{Ca1^[2, 6;0]^ Ca2^[2, 5; 0]^ Ge^[6, 5; 12^}
	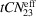 = 8.71	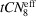 = 3.86	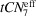 = 3.90	{0.003, 0.020, 0.022}°
				
BaH_2_	Ba (*V* = 28.1 Å^3^)	H1 (*V* = 15.0 Å^3^)	H2 (*V* = 12.1 Å^3^)	
(*a*)	*tCN*_5_(Ba) 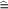 [1H1, 4H2; 0]	*tCN*_1_(H1) 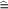 [1Ba, 0; 0]	*tCN*_4_(H2) 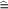 [4Ba, 0; 0]	{Ba^[1, 4; 0]^ H1^[1, 0; 0]^ H2^[4, 0; 0]^}
	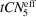 = 4.85	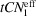 = 0.92	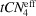 = 3.93	{0.42, 0.36, 0.22}°
(*b*)	*tCN*_7_(Ba) 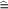 [3H1, 4H2; 0]	*tCN*_3_(H1) 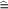 [3Ba, 0; 0]	*tCN*_4_(H2) 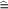 [4Ba, 0; 0]	{Ba^[3, 4; 0]^ H1^[3, 0; 0]^ H2^[4, 0; 0]^}
	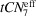 = 6.46	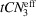 = 2.52	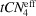 = 3.93	{0.43, 0.38, 0.30}°
(*c*)	*tCN*_9_(Ba) 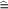 [5H1, 4H2; 0]	*tCN*_5_(H1) 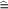 [5Ba, 0; 0]	*tCN*_4_(H2) 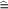 [4Ba, 0; 0]	{Ba^[5, 4; 0]^ H1^[5, 0; 0]^ H2^[4, 0; 0]^}
	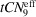 = 7.81	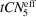 = 3.88	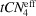 = 3.93	{**1, 1, 1**}°
(*d*)	*tCN*_15_(Ba) 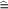 [6H1, 5H2; 4Ba]	*tCN*_16_(H1) 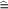 [6Ba, 6H2; 4H1]	*tCN*_13_(H2) 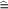 [5Ba, 6H1; 2H2]	{Ba^[6, 5; 4]^ H1^[6, 6; 4]^ H2^[5, 6; 2]^}
	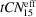 = 7.97	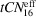 = 4.98	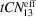 = 4.93	{0.003, 0.022, 0.026}°

## Appendix A Geometrical weighting schemes

In the present study three different weighting schemes denoted as ‘sc1’ [equation (11*a*[Disp-formula fd11a])], ‘sc2’ [equation (11*b*[Disp-formula fd11b])] and ‘lin’ [equation (11*c*[Disp-formula fd11c])] have been employed to quantify the coordination gaps in a solid angle sequence of the coordination string **S**^ω^°(*A*) [equation (9)].

Semicircle weighting schemes ‘sc1’ and ‘sc2’ are derived from area relations between different semicircle segments (Fig. 11 ▸ top, middle). Note that only half of the semicircles (quadrants) is shown due to lack of space, but the areas are computed for the whole semicircles. The height of the semicircle segment represents the ω° ordinate axis ranging from 0 to 1 according to the normalized solid angle values ω_*j*_°(*A*) [equation (9)].

In the present general case ω_*j*_°(*A*) represents a continuous variable (*j* → ∞) with values 0 ≤ ω_*j*_°(*A*) ≤ 1, and it is not related to a specific atomic coordination string. Therefore, the notation has been simplified hereafter to indicate the generalization from discrete values ω_*j*_°(*A*) in a coordination string to a continuous variable ω° according to ω_*j*_°(*A*) → ω° and ^scheme^*p*_*j*_(*A*) → ^scheme^*p*(ω°). The area 0.5*r*^2^π of a semicircle of the unit circle (*r* = 1) amounts to 0.5π [equation (15)]. This represents the reference area ^sc1,sc2^*s*(1) for the semicircle weighting schemes:

Scheme ‘sc2’ keeps the circular cap of the semicircle and cuts away varying amounts of its base (Fig. 11 ▸ middle, gray area), which yields areas of circular segments. In contrast, scheme ‘sc1’ cuts away varying amounts of the circular cap (Fig. 11 ▸ top, gray area), such that the remaining geometrical object is a different kind of ‘circular segment’ (not in the strict geometrical meaning). The areas ^*scheme*^*s*(ω°) of the ‘circular segments’ employed in schemes ‘sc1’ [equation (16)] and ‘sc2’ [equation (17)] are colored green and blue, respectively (Fig. 11 ▸ top, middle). Although only half of the semicircles is shown in Fig. 11 ▸, equations (15)–(17) calculate the full semicircle and segment areas:

From this geometric construction it becomes clear that , where the equality between both areas is only valid at the two endpoints ω° = 0 or 1.

This relation is passed on to the coordination probability values ^sc1^*p*(ω°) and ^sc2^*p*(ω°) [equations (11*a*), (11*b*)], which are obtained by dividing the segment areas by the reference area (Fig. 12 ▸),

In Fig. 11 ▸ (top, middle) [with Δω° = (1 − 0.9) = 0.1] it can be seen that at high values ω° > 0.5 a given difference Δω° has a much larger effect on the area difference (gray areas represent the part cut off from the full area) in the ‘sc2’ scheme than in the ‘sc1’ scheme. This specific feature leads to high sensitivity of gap weights ^*scheme*^*w*_j_(*A*) [equation (12)] on gap values Δω° for scheme ‘sc2’ at the beginning of the coordination sequence (ω° > 0.5), and a lower sensitivity closer to the end (ω° < 0.5). It is indicated by the derivative curve d^sc2^*p*/dω° (Fig. 12 ▸). The opposite behavior is shown by scheme ‘sc1’, such that the ‘sc1’ and ‘sc2’ derivative curves intersect at ω° = 0.5.

This dependence of coordination-gap weights on ω° does not occur for linear weighting, as exemplified in Fig. 11 ▸ (bottom), with a given difference of *e.g.* Δω° = 0.1. The reference area of a unit square (with side length of 1) amounts to

and the partial area for given ω° is

Thus, the coordination probability is obtained by

The sub-area ^lin^*s*(ω°) − ^lin^*s*(ω°+Δω°) = Δω° (orange) of the unit square (pale yellow area) is only dependent on Δω°, and not on the position ω° in which it is located, which is indicated by the constant gradient curve d^lin^*p*/dω° = 1 in Fig. 12 ▸ (broken orange line).

## Appendix B Notes on the quantities defined in the coordination scenario protocol

As can be seen from equations (11*a*)–(11*c*) [step (iv) in Section 3.2], the value ^*scheme*^*p*_*j*_(*A*) only depends on the value ω_*j*_°(*A*) and is not affected by the internal structure of the coordination string *S_i_*^ω^°(*A*). Given the decreasing values of ω_*j*_°(*A*) and associated ^*scheme*^*p*_*j*_(*A*) along the coordination string *j* = 1 … *k* (Appendix *A*[App appa]), ω_*j*=1_°(*A*) = 1 yields the highest coordination probability ^*scheme*^*p*_*j*=1_(*A*) = 1 and ω_*k*+1_°(*A*) = 0, the lowest probability ^*scheme*^*p*_*j*=*k*+1_(*A*) = 0. This low value is only obtained because of the inclusion of the final (hypothetical) neighbor with value ω_*k*+1_°(*A*) = 0, since the CN *k* + 1 obtained by including a neighbor with zero solid angle is ultimately improbable. In contrast, inclusion of a final neighbor with nonzero ω_*j*_°(*A*) value (*j* ≤ *k*) will give a nonzero probability ^*scheme*^*p_j_*(*A*) related to this neighbor’s solid angle ω_*j*_°(*A*).

The coordination probability values obtained using the semicircle type of weighting function *f*(ω°) [step (iv) in Section 3.2] can be geometrically considered as the first step away from linear weighting described by a (semi-)rectangle weighting function for comparison. These purely geometry-based weighting functions are completely determined by a specific geometrical property of the circle and rectangle, namely, the development of the filled area size obtained by increasingly filling the region encompassed by the bounding lines of the circle and the rectangle, respectively (Appendix *A*[App appa]). As result, they do not have free parameters, which is considered as an advantage in the present study.

Using linear and semicircle weighting [equations (11*a*)–(11*c*)], the sub-coordination likelihood values are given as a vector with three components ‘sc2’ [equation (11*b*[Disp-formula fd11b])], ‘lin’ [equation (11*c*[Disp-formula fd11c])] and ‘sc1’ [equation (11*a*[Disp-formula fd11a])], according to the methods’ emphasis of favoring coordination gaps at the beginning (‘sc2’) or the end (‘sc1’) of the coordination sequence, where method ‘lin’ does not exhibit any preferential behavior (Appendix *A*[App appa]). Like the separate coordination likelihoods [step (v) in Section 3.2], the scenario weights [step (vii) in Section 3.2] are restricted to values 0 ≤ ^*scheme*^*W*[*j*(1), *j*(2), …, *j*(*N*)] ≤ 1. However, their sum for a given compound in general does not have a well defined upper bound, which impedes their direct interpretation as coordination scenario likelihoods. For this purpose, normalization of each average sub-coordination scenario weight by the sum over all possible sub-coordination scenario weights yields the required property for a sub-coordination likelihood interpretation. This effort has not been undertaken in the present study, because this normalization procedure does not change the ratios of scenario weights between different scenarios, while the values themselves are dependent on the number of actually possible scenarios for each compound. As an aid for easier comparison of different scenarios, in the present study scenario weights were additionally normalized with respect to the most dominant scenario for each weighting scheme. This is indicated by a superscript for the scenario weights {^sc2^*W*, ^lin^*W*, ^sc1^*W*}° (*cf*. Section 4).
