# Supplementary material for: Topological coordination numbers and coordination reciprocity from electron-density distributions
Source: Acta Crystallogr A Found Adv. 2025 Apr 28;81(Pt 3):221–44. doi: 10.1107/S2053273325002347 (PMC12053493; doi:10.1107/S2053273325002347)
Supplement: Supplementary file 1 [file a-81-00221-sup1.pdf]

FOUNDATIONS ADVANCES

Volume 81 (2025)

Supporting information for article:

Topological coordination numbers and coordination reciprocity from electron-density distributions

Frank R. Wagner, Riccardo Freccero and Yuri Grin

S1. Crystallographic Information on structural parameters employed in the computations

Table S1. Structure parameters (optimized) for the systems studied. Pearson symbol, space group, and Wyckoff sequence for structure types *fcc*: cF4 (225) *Fm*-3*m* - *a*; *bcc*: cI2 (229) *Im*-3*m* - *a*; *hcp*: hP2 (194) $P6_3/mmc$ - *c*; diamond (*dia*) type: cF8 (227) *Fd*-3*m* - *a*; rock salt (*NaCl*) type: cF8 (225) *Fm*-3*m* - *ba*; *CsCl* type: cP2 (221) *Pm*-3*m* - *ba*; Zinc blende (*ZnS*) type: cF8 (216) *F*-43*m* – *ca*.

Compound (*structure*)	Lattice parameters (Å)	Compound (*structure*)	Lattice parameters (Å)
Ca (*fcc*)	5.5313	K (*bcc*)	5.2780
Rh (*fcc*)	3.8277	Mo (*bcc*)	3.1582
Pd (*fcc*)	3.9401	Mg (*hcp)*	3.1954, 5.1872
C (*dia*)	3.5718	Ti (*hcp*)	2.9320, 4.6550
Si (*dia*)	5.4728	Zn (*hcp*)	2.6618, 4.9546
Ge (*dia*)	5.7661		
NaCl (*NaCl*)	5.6960	BN (*ZnS*)	3.6249
LiI (*NaCl*)	6.0072	BP (*ZnS*)	4.5490
KI (*NaCl*)	7.1597	GaN (*ZnS*)	4.5493
RbI (*NaCl*)	7.4555	GaP (*ZnS*)	5.5093
RbF (*NaCl*)	5.7399	GaAs (*ZnS*)	5.7509
CsCl (*CsCl*)	4.1959	GaSb (*ZnS*)	6.2139
CsI (*CsCl*)	4.6443		

Table S2. Structure parameters (standardized data) for TiNiSi representatives. Pearson symbol, space group, and Wyckoff sequence: oP12 (62) *Pnma* - c^3. Species occupying the Si type of site of the prototype TiNiSi are indicated according to *species* ≙ “*Si*”.

Compound	*a*, *b*, *c* (Å)	“*Ti*”: *x*, ¼, *z*	“*Ni*”: *x*, ¼, *z*	“*Si*”: *x*, ¼, *z*
TiNiSi<br>Si ≙ “*Si*“	6.139<br>3.661<br>7.006	0.0211<br>¼<br>0.6816	0.6417<br>¼<br>0.4405	0.2654<br>¼<br>0.3764
Co_2Si<br>Si ≙ “*Si*“	4.906<br>3.729<br>7.096	0.0392<br>¼<br>0.7198	0.6711<br>¼<br>0.4360	0.2077<br>¼<br>0.3909
SrLiAs<br>As ≙ “*Si*“	7.6473<br>4.5194<br>8.1519	0.00882<br>¼<br>0.70167	0.65176<br>¼<br>0.43103	0.27678<br>¼<br>0.40343
$PbCl_2$<br>Pb ≙ “*Si*“	7.6204<br>4.5342<br>9.0452	0.02020<br>¼<br>0.65290	0.64250<br>¼<br>0.42170	0.23770<br>¼<br>0.40410

$BaCl_2$<br>Ba ≙ "*Si*"	7.9516<br>4.8054<br>9.5131	0.02238<br>¼<br>0.66577	0.64172<br>¼<br>0.42637	0.24675<br>¼<br>0.38810
Ba_2Ge<br>Ge ≙ "*Si*"	8.4874<br>5.4335<br>10.1695	0.01999<br>¼<br>0.67154	0.64622<br>¼<br>0.41680	0.24892<br>¼<br>0.39913
Ca_2Ge<br>Ge ≙ "*Si*"	7.6749<br>4.8571<br>9.0896	0.01963<br>¼<br>0.67742	0.65365<br>¼<br>0.42555	0.25257<br>¼<br>0.39499
BaH_2<br>Ba ≙ "*Si*"	6.8033<br>4.1523<br>7.8259	0.02810<br>¼<br>0.68148	0.64534<br>¼<br>0.42831	0.26093<br>¼<br>0.38794

S2. An example for CR violation in the BS type of coordination numbers

Table S3. Coordination numbers for the CaB_6 case given by the Brunner-Schwarzenbach/Villars-Daams (BS/VD) type of coordination scheme violating coordination reciprocity (CR). The B atom ligands with distances below the local distance gap are marked grey, the ones for Ca in yellow; the largest distance gap for B and Ca species yields a 5-coordination of B and a 24-coordination of Ca; omission of 4 Ca ligands (yellow) of B violates CR, because 24 B atoms are considered being coordinated to Ca (yellow).
Structure data for cP7–1a.6f type of structure from (Chen et al., 2004), see footnote.

CN(B)	Dist(B–neighbor) / Å	CN(Ca)	Dist(Ca–neighbor) / Å
1	167.6, B	./.	./.
2	175.0, B	./.	./.
3	175.0, B	./.	./.
4	175.0, B	./.	./.
5	175.0, B	./.	./.
?	305.3, Ca	1	305.3, B
?	305.3, Ca	2	305.3, B
?	305.3, Ca	3	305.3, B
?	305.3, Ca	4	305.3, B
?	316.6, B	5	305.3, B
?	316.6, B	6	305.3, B
?	316.6, B	7	305.3, B
?	316.6, B	8	305.3, B
?	316.6, B	9	305.3, B
?	316.6, B	10	305.3, B
?	316.6, B	11	305.3, B
?	316.6, B	12	305.3, B
?	412.1, B	13	305.3, B
	…	14	305.3, B
		15	305.3, B
		16	305.3, B
		17	305.3, B

		18	305.3, B
		19	305.3, B
		20	305.3, B
		21	305.3, B
		22	305.3, B
		23	305.3, B
		24	305.3, B
		?	415.1, Ca
			…

Ref.: Chen, C. H., Aizawa, T., Iyi, Sato, A. & Otani S. *J. Alloys Compd*. (2004), 366, L6–L8.

S3. Coordination polyhedra and atomic domains

The atomic domain of a species obtained from a Voronoi-Dirichlet (VD) construction scheme is always convex, and the VD polyhedra for a given structure yield an exhaustive space partitioning. The QTAIM atomic domains also yield an exhaustive space partitioning, but they often display non-convex shapes. In certain cases, e.g. where the non-convexity concerns only the presence of concave faces, an approximated convex polyhedral domain topologically similar (same numbers of vertices, edges, and faces with vertex configurations identical to the QTAIM ones can be constructed as shown for the *bcc* (body centered cubic) type of structure (section 4.2). However, this is not mathematically guaranteed in general. In any case, from the atoms identified to be coordinated to a central atom (‘ligands’) by a VDP or tCN type of methodology, a coordination polyhedron around the central species can be constructed such that every ligand is at least 2-fold connected by other ligands, and the resulting faces are at least 3-gonal. Noteworthy, the resulting coordination polyhedron does not necessarily need to be convex, even if it is derived from VD partitioning (see the diamond structure section 4.2). This means that coordination numbers obtained from VD and tCN types of space partitioning can go beyond the restriction on convex coordination environments imposed in other schemes, like e.g. the Villars-Daams type of coordination environment types (Daams & Villars, 1997).
From the non-restriction of coordination polyhedra to be always convex, it can be already concluded that a VDP domain and its related coordination polyhedron cannot be dual to each other in general. However, there are special cases where this duality is valid. The VDP domain may be related to the dual of the coordination polyhedron if the selected coordination polyhedron is convex, and if it is sufficiently strictly defined, e.g. displaying vertex transitivity (Table S2). In these special cases, the Voronoi-Dirichlet construction yields a coordination polyhedron, which is related to the dual of the VD polyhedron.

Table S4. Coordination polyhedra, Voronoi polyhedra, and their duals for 1-species structures. The Pearson code has been extended by the Wyckoff sequence including site multiplicity. The symbol vdX_n (Ref. see footnote) associated with the polyhedral vertices enumerates the number of vertices *n* with vertex degree (vd) *X* (the same vertex degree appearing twice means that the corresponding vertices are non-equivalent); the face symbol Z^m associated with the polyhedral faces enumerates the number *m* of polyhedral *Z*-gonal faces.

	Coordination *polyhedron*	Dual of coordination *polyhedron*	Voronoi *polyhedron*	Dual of Voronoi polyhedron
Scp, *cP*1-1*a*	*Octahedron*<br>6v: vd4_6<br>8f: 3^8<br>Platonic	*Cube*<br>8v: vd3_8<br>6f: 4^6<br>Platonic	*Cube*<br>8v: vd3_8<br>6f: 4^6<br>Platonic	*Octahedron*<br>6v: vd4_6<br>8f: 3^8<br>Platonic
Fcc, *cF*4-4*a*	*Cuboctahedron*<br>12v: vd4_{12}<br>14f: $3^8.4^6$<br>Archimedean	*Rhombic dodecahedron*<br>14v: vd$3_8.4_6$<br>12f: 4^{12}<br>Catalan	*Rhombic dodecahedron*<br>14v: vd4_{12}<br>12f: 4^{12}<br>Catalan	*Cuboctahedron*<br>12v: vd4_{12}<br>14f: $3^8.4^6$<br>Archimedean
Bcc, *cI*2-2*a*	*Rhombic dodecahedron*<br>14v: vd$3_8.4_6$<br>12f: 4^{12}<br>Catalan	*Cuboctahedron*<br>12v: vd4_{12}<br>14f: $3^8.4^6$<br>Archimedean	*Truncated octahedron*<br>12v: vd3_{12}<br>14f: $4^6.6^8$<br>Archimedean	*Tetrakis hexahedron*<br>14v: vd$4_6.6_8$<br>24f: 3^{24}<br>Catalan
Hcp, *hP*2-2*c*	*Anticuboctahedron*<br>12v: vd$4_6.4_6$<br>14f: $3^2.3^6.4^6$<br>Johnson	*Trapezo-rhombic dodecahedron*<br>14v: vd$3_2.3_6.4_6$<br>12f: $4^6.4^6$<br>Johnson	*Trapezo-rhombic dodecahedron*<br>14v: vd$3_2.3_6.4_6$<br>12f: $4^6.4^6$<br>Johnson	*Anticuboctahedron*<br>12v: vd$4_6.4_6$<br>14f: $3^2.3^6.4^6$<br>Johnson
Diamond, *cF*8-8*a*	Non-convex, 16v<br>Nested<br>Tetrahedron -><br>&<br>Cuboctahedron ->	Non-convex, 16f<br>Nested<br>-> Tetrahedron<br>&<br>-> Rhombic dodecahedron	*Triakis truncated tetrahedron*<br>16v: vd$3_4.4_{12}$<br>16f: $3^{12}.6^4$<br>convex	*Order-3 truncated triakis tetrahedron*<br>“trigonally-truncated triakis tetrahedron”<br>convex

Ref.: Wagner, F. R. & Grin, Yu. (2024) *Inorg. Chem*. **63**, 20205–20216.

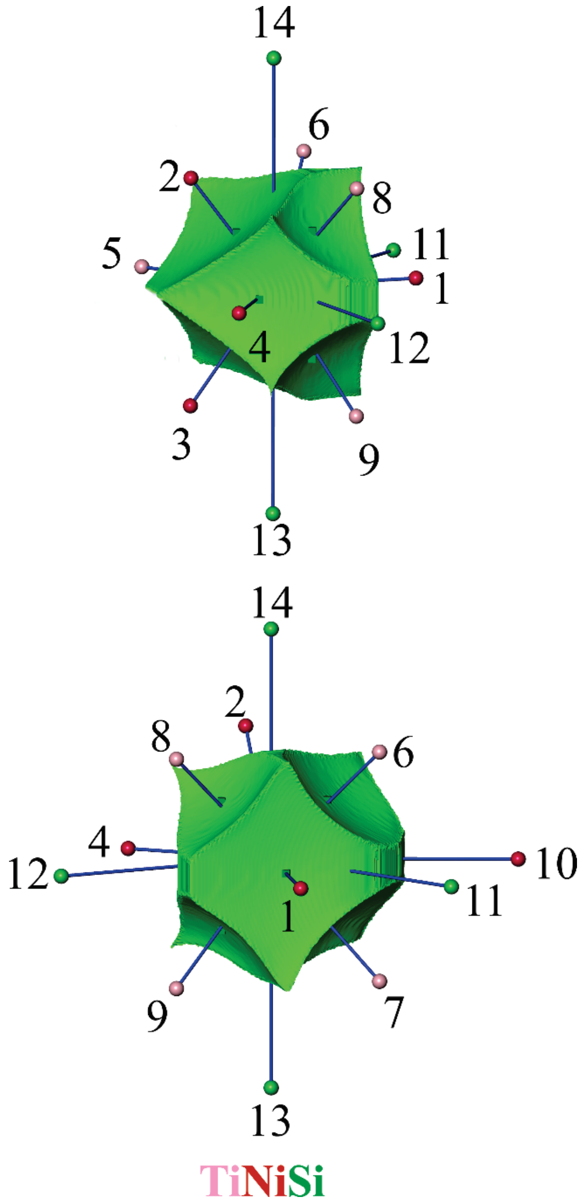

S4. Example: Topological characteristics of the QTAIM Si atom in TiNiSi

Figure S1. Shape of the QTAIM Si atom with tCN_{tot}(Si) = 14 in the crystal structure of TiNiSi in two projections. The numbers represent the neighbor index in Tables S3 and S6.

Neighbors 2(Ni), 6(Ti), 8(Ti) and 3(Ni), 7(Ti), 9(Ti) build a trigonal prism; neighbors 1(Ni), 4(Ni), 5(Ti) on the mirror plane represent the capping atoms in the traditional description of the Si 9-coordination environment.

A rough idea about the values of the new description parameters (steps 1 – 5 of workflow protocol in main text section 3.2) can be obtained from the analysis of topology of electron density in TiNiSi revealing QTAIM silicon atom of complex shape. Fourteen atoms significantly (see cutoff value mentioned above) share surfaces with the silicon species, i.e. tCN_{tot}(Si) = 14 (Fig. S1). The coordination sequence for silicon species $\mathbf{S}^{\omega}$(Si) contains 14 values of the solid angles $\omega_j(A, B)$ and is terminated by zero (Table S3). It is worth mentioning that the decay of the solid angles in $\mathbf{S}^{\omega}$(Si) is not in line with the change of interatomic distances. The individual contribution of each neighbor to the effective topological coordination number tCN_k^{eff}(Si) is obtained by global normalization with the largest $\omega_1(A_i)$ (A_i = Ti, Ni, Si) value of all species. In this compound, like in all TiNiSi type of compounds investigated, for the "*Si*" type site the local ω_1("*Si*") and the global normalization $\omega_{\text{ref}}^{\text{all}}$ is identical, such that $\omega_1°$("*Si*") = $tCN_{1,1}^{\text{eff}}$("*Si*") = 1. Further information about the effective coordination sphere of Si can be obtained from the analysis of the coordination probabilities $^{scheme}p_j$(Si) (last three columns in Table S3). The difference between two subsequent coordination probabilities defines coordination likelihoods $^{scheme}w_j$(Si) (Eq. 12, main text), which represent coordination gap values. Note that on this level CR has not yet been invoked to find (sub-)coordination scenarios (steps 6 – 8 of workflow protocol in section 3.2). This is done in the complete treatment in section 4.3 of the main text.

Table S5. Topological characteristics for the QTAIM Si species in the crystal structure of TiNiSi. The coordinating species are ordered according to $\mathbf{S}^{\omega}$(Si).

Neighbor atom *B*	Distance d(Si–*B*), Å	*j*	ω_j(Si,*B*) (Eq. 5)	$\omega_j°$(Si) = $tCN_{j,j}^{\text{eff}}$(Si) (Eqs. 6a,b, 9)	$^{\text{sc2}}p_j$(Si) (Eq. 11b)	$^{\text{lin}}p_j$(Si) (Eq. 11c)	$^{\text{sc1}}p_j$(Si) (Eq. 11a)
Ni	2.346	1	4.4485	1.0000	1.0000	1.0000	1.0000
Ni	2.307	2	4.3323	0.9739	0.9668	0.9739	0.9950
Ni	2.307	3	4.3323	0.9739	0.9668	0.9739	0.9950
Ni	2.353	4	3.7268	0.8378	0.7944	0.8378	0.9235
Ti	2.612	5	3.2711	0.7353	0.6670	0.7353	0.8432
Ti	2.571	6	3.1763	0.7140	0.6409	0.7140	0.8245
Ti	2.571	7	3.1763	0.7140	0.6409	0.7140	0.8245
Ti	2.633	8	2.5035	0.5628	0.4616	0.5628	0.6766
Ti	2.633	9	2.5035	0.5628	0.4616	0.5628	0.6766

Ni	3.855	10	0.1810	0.0407	0.0098	0.0407	0.0517
Si	3.544	11	0.1412	0.0317	0.0068	0.0317	0.0403
Si	3.544	12	0.1412	0.0317	0.0068	0.0317	0.0403
Si	3.661	13	0.0412	0.0093	0.0011	0.0093	0.0117
Si	3.661	14	0.0412	0.0093	0.0011	0.0093	0.0117
		15	0	0	0	0	0

S5. Extended tables of selected (sub-)coordination scenarios for 1-, 2- and 3-species (TiNiSi) type of structures and compounds.

Table S6. 1-species element structures. Different scenarios '*scene*' are denoted (a), (b), …; for each species listed coordination likelihoods [^{sc2}w, ^{lin}w, ^{sc1}w] represent also (sub-)coordination scenario weights {^{sc2}W, ^{lin}W, ^{sc1}W}; {$^{scheme}W$}° denotes normalized scenario weights, where a value of **1** corresponds to the scenario with highest scenario weight $^{scheme}W$ (denoted 'max' in the text) for the respective weighting *scheme*. The last scenario given always corresponds to inclusion of all domain surfaces (denoted 'tot' in the text).

Compound,<br>*Scene*	*Species1*<br>Likelihoods [$^{scheme}w$]	Scenario<br>{$^{scheme}W$}, {$^{scheme}W$}°
Fcc-M = Ca, Rh, Pd	$tCN_{12}(M)$ ≙ [12*M*]<br>[1.0, 1.0, 1.0]<br>tCN_{12}^{eff} = 12	{$M^{[12]}$}<br>{1.0, 1.0, 1.0}<br>**{1, 1, 1}**°
Bcc-K	K	
(a)	tCN_8(K) ≙ [8K]<br>[0.89, 0.79, 0.74]<br>tCN_8^{eff} = 8	{$K^{[8]}$}<br>{0.89, 0.79, 0.74}<br>**{1, 1, 1}**°
(b)	tCN_{14}(K) ≙ [14K]<br>[0.11, 0.21, 0.26]<br>tCN_{14}^{eff} = 9.22	{$K^{[14]}$}<br>{0.11, 0.21, 0.26}<br>{0.12, 0.26, 0.35}°
Bcc-Mo	Mo	
(a)	tCN_8(Mo) ≙ [8Mo]<br>[0.89, 0.80, 0.74]<br>tCN_8^{eff} = 8	{$Mo^{[8]}$}<br>{0.89, 0.80, 0.74}<br>**{1, 1, 1}**°
(b)	tCN_{14}(Mo) ≙ [14Mo]<br>[0.11, 0.20, 0.26]<br>tCN_{14}^{eff} = 9.18	{$Mo^{[14]}$}<br>{0.11, 0.20, 0.26}<br>{0.12, 0.25, 0.34}°
Hcp-Ti	Ti (*c*/*a* = 1.588)	
(a)	tCN_6(Ti) ≙ [6Ti]<br>[0.14, 0.11, 0.05]<br>tCN_6^{eff} = 6	{$Ti^{[6]}$}<br>{0.14, 0.11, 0.05}<br>{0.16, 0.12, 0.05}°
(b)	tCN_{12}(Ti) ≙ [12Ti]<br>[0.86, 0.89, 0.95]<br>tCN_{12}^{eff} = 11.27	{$Ti^{[12]}$}<br>{0.86, 0.89, 0.95}<br>**{1, 1, 1}**°
Hcp-Mg	Mg (*c*/*a* = 1.623)	
(a)	tCN_6(Mg) ≙ [6Mg]<br>[0.23, 0.18, 0.09]<br>tCN_6^{eff} = 6	{tCN_6(Mg)}, {$Mg^{[6]}$}<br>{0.23, 0.18, 0.09}<br>{0.30, 0.22, 0.10}°
(b)	tCN_{12}(Mg) ≙ [12Mg]<br>[0.77, 0.82, 0.91]<br>tCN_{12}^{eff} = 10.85	{$Mg^{[12]}$}<br>{0.77, 0.82, 0.91}<br>**{1, 1, 1}**°
Hcp-Zn	Zn (*c*/*a* = 1.861)	

(a)	*tCN*6(Zn) ≙ [6Zn]<br>[0.45, 0.36 0.24]<br>tCN_6^{eff} = 6	{Zn[6]}<br>{0.45, 0.36 0.24}<br>{0.82, 0.56, 0.32}°
(b)	*tCN*12(Zn) ≙ [12Zn]<br>[0.55, 0.64, 0.76]<br>tCN_{12}^{eff}= 9.79	{Zn[12]}<br>{0.55, 0.64, 0.76}<br>**{1, 1, 1}**°
Diamond		
C	C	
(a)	*tCN*4(C) ≙ [4C]<br>[1.00, 0.99, 0.99]<br>tCN_4^{eff} = 4	{C[4]}<br>{1.00, 0.99, 0.99}<br>**{1, 1, 1}**°
(b)	*tCN*16(C) ≙ [16C]<br>[0.00, 0.01, 0.01]<br>tCN_{16}^{eff} = 4.07	{C[16]}<br>{0.00, 0.01, 0.01}<br>{0.00, 0.01, 0,01}°
Si	Si	
(a)	*tCN*4(Si) ≙ [4Si]<br>[1.00, 1.00, 0.99]<br>tCN_4^{eff} = 4	{Si[4]}<br>{1.00, 1.00, 0.99}<br>**{1, 1, 1}**°
(b)	*tCN*16(Si) ≙ [16Si]<br>[0.00, 0.00, 0.01]<br>tCN_{16}^{eff} = 4.05	{Si[16]}<br>{0.00, 0.00, 0.01}<br>{0.00, 0.00 0.01}°
Ge	Ge	
(a)	*tCN*4(Ge) ≙ [4Ge]<br>[1.00, 0.99, 0.99]<br>tCN_4^{eff} = 4	{Ge[4]}<br>{1.00, 0.99, 0.99}<br>**{1, 1, 1}**°
(b)	*tCN*16(Ge) ≙ [16Ge]<br>[0.00, 0.01, 0.01]<br>tCN_{16}^{eff} = 4.07	{Ge[16]}<br>{0.00, 0.01, 0.01}<br>{0.00, 0.01 0.01}°

Table S7. Binary structures. For each *species* are listed: QTAIM atomic volume *V*, and, for each scenario '*scene*' (a, b, …), coordination sequence, coordination likelihoods [^{sc2}w, ^{lin}w, ^{sc1}w] and effective coordination number; for each scenario are listed: CR consistent coordinations, scenario weights {^{sc2}W, ^{lin}W, ^{sc1}W} and normalized scenario weights {^{sc2}W, ^{lin}W, ^{sc1}W}°, where a value of **1** corresponds to the dominant scenario with highest scenario weight $^{scheme}W$ (denoted 'max' in the text) for the respective weighting *scheme*. The last scenario given always corresponds to inclusion of all domain surfaces (denoted 'tot' in the text).

Compound, *Scene*	*Species1*	*Species2*	Scenario weights
Zn-Blende type			
BN	B (V = 1.66 Å^3)	N (V = 10.3 Å^3)	
(a)	tCN_4(B) ≙ [4N; 0]<br>[1.00, 1.00, 1.00]<br>tCN_4^{eff} = 4	tCN_4(N) ≙ [4B; 0]<br>[0.95, 0.87, 0.84]<br>tCN_4^{eff} = 4	{$B^{[4;\,0]}$ $N^{[4;\,0]}$}<br>{0.97, 0.93, 0.91}<br>{**1, 1, 1**}°
(b)	tCN_4(B) ≙ [4N; 0]<br>[1.00, 1.00, 1.00]<br>tCN_4^{eff} = 4	tCN_{16}(N) ≙ [4B; 12N]<br>[0.05, 0.13, 0.16]<br>tCN_{16}^{eff} = 5.52	{$B^{[4;\,0]}$ $N^{[4;\,12]}$}<br>{0.23, 0.36, 0.41}<br>{0.24, 0.39, 0.45}°
BP	B (V = 6.97 Å^3)	P (V = 16.6 Å^3)	
(a)	tCN_4(B) ≙ [4P; 0]<br>[1.00, 1.00, 1.00]<br>tCN_4^{eff} = 4	tCN_4(P) ≙ [4B; 0]<br>[0.99, 0.96, 0.95]<br>tCN_4^{eff} = 4	{$B^{[4;\,0]}$ $P^{[4;\,0]}$}<br>{1.00, 0.98, 0.98}<br>{**1, 1, 1**}°
(b)	tCN_4(B) ≙ [4P; 0]<br>[1.00, 1.00, 1.00]<br>tCN_4^{eff} = 4	tCN_{16}(P) ≙ [4B; 12P]<br>[0.01, 0.04, 0.05]<br>tCN_{16}^{eff}= 4.40	{$B^{[4;\,0]}$ $P^{[4;\,12]}$}<br>{0.09, 0.19, 0.22}<br>{0.09, 0.19, 0.22}°
GaN	Ga (V = 10.1 Å^3)	N (V = 13.4 Å^3)	
(a)	tCN_4(Ga) ≙ [4N; 0]<br>[1.00, 0.996, 0.995]<br>tCN_4^{eff} = 4	tCN_4(N) ≙ [4Ga; 0]<br>[0.996, 0.979, 0.974]<br>tCN_4^{eff} = 4	{$Ga^{[4;\,0]}$ $N^{[4;\,0]}$}<br>{1.00, 0.99, 0.98}<br>{**1, 1, 1**}°
(b)	tCN_4(Ga) ≙ [4N; 0]<br>[1.00, 0.996, 0.995]<br>tCN_4^{eff} = 4	tCN_{16}(N) ≙ [4Ga; 12N]<br>[0.004, 0.02, 0.026]<br>tCN_{16}^{eff} = 4.21	{$Ga^{[4;\,0]}$ $N^{[4;\,12]}$}<br>{0.06, 0.14, 0.16}<br>{0.06, 0.14, 0.16}°
GaP	Ga (V = 15.9 Å^3)	P (V = 25.9 Å^3)	
(a)	tCN_4(Ga) ≙ [4P; 0]<br>[1.000, 0.999, 0.999]<br>tCN_4^{eff} = 4	tCN_4(P) ≙ [4Ga; 0]<br>[0.995, 0.973, 0.966]<br>tCN_4^{eff} = 4	{$Ga^{[4;\,0]}$ $P^{[4;\,0]}$}<br>{1.00, 0.986, 0.982}<br>{**1, 1, 1**}°
(b)	tCN_4(Ga) ≙ [4P; 0]<br>[1.000, 0.999, 0.999]<br>tCN_4^{eff} = 4	tCN_{16}(P) ≙ [4Ga; 12P]<br>[0.005, 0.027, 0.034]<br>tCN_{16}^{eff} = 4.28	{$Ga^{[4;\,0]}$ $P^{[4;\,12]}$}<br>{0.072, 0.163, 0.184}<br>{0.07, 0.17, 0.19}°
GaAs	Ga (V = 18.2 Å^3)	As (V = 29.3 Å^3)	
(a)	tCN_4(Ga) ≙ [4As; 0]<br>[1.000, 0.999, 0.999]<br>tCN_4^{eff} = 4	tCN_4(As) ≙ [4Ga; 0]<br>[0.995, 0.974, 0.966]<br>tCN_4^{eff} = 4	{$Ga^{[4;\,0]}$ $As^{[4;\,0]}$}<br>{0.997, 0.986, 0.983}<br>{**1, 1,1**}°
(b)	tCN_4(Ga) ≙ [4As; 0]<br>[1.000, 0.999, 0.999]<br>tCN_4^{eff} = 4	tCN_{16}(As) ≙ [4Ga; 12As]<br>[0.005, 0.026, 0.034]<br>tCN_{16}^{eff} = 4.27	{$Ga^{[4;\,0]}$ $As^{[4;\,12]}$}<br>{0.07, 0.16, 0.18}<br>{0.07, 0.16, 0.18}°

GaSb	Ga (V = 22.5 Å^3)	Sb (V = 37.5 Å^3)	
(a)	tCN_4(Ga) ≙ [4Sb; 0]<br>[1.000, 0.999, 0.999]<br>tCN_4^{eff} = 4	tCN_4(Sb) ≙ [4Ga; 0]<br>[0.994, 0.970, 0.962]<br>tCN_4^{eff} = 4	{$Ga^{[4;\,0]}$ $Sb^{[4;\,0]}$}<br>{0.997, 0.985, 0.980}<br>**{1, 1, 1}**°
(b)	tCN_4(Ga) ≙ [4Sb; 0]<br>[1.000, 0.999, 0.999]<br>tCN_4^{eff} = 4	tCN_{16}(Sb) ≙ [4Ga; 12Sb]<br>[0.006, 0.030, 0.038]<br>tCN_{16}^{eff} = 4.32	{$Ga^{[4;\,0]}$ $Sb^{[4;\,12]}$}<br>{0.079, 0.173, 0.196}<br>{0.079, 0.18, 0.20}°
Rock-salt type			
NaCl	Na (V = 9.72 Å^3)	Cl (V = 36.5 Å^3)	
(a)	tCN_6(Na) ≙ [6Cl; 0]<br>[1.00, 1.00, 1.00]<br>tCN_6^{eff} = 6	tCN_6(Cl) ≙ [6Na; 0]<br>[0.74, 0.62, 0.53]<br>tCN_6^{eff} = 6	{$Na^{[6;\,0]}$ $Cl^{[6;\,0]}$}<br>{0.86, 0.79, 0.73}<br>**{1, 1, 1}**°
(b)	tCN_6(Na) ≙ [6Cl; 0]<br>[1.00, 1.00, 1.00]<br>tCN_6^{eff} = 6	tCN_{18}(Cl) ≙ [6Na; 12Cl]<br>[0.26, 0.38, 0.47]<br>tCN_{18}^{eff} = 10.48	{$Na^{[6;\,0]}$ $Cl^{[6;\,12]}$}<br>{0.51, 0.62, 0.69}<br>{0.59, 0.78, 0.95}°
LiI	Li (V = 4.50 Å^3)	I (V = 49.7 Å^3)	
(a)	tCN_6(Li) ≙ [6I; 0]<br>[1.00, 1.00, 1.00]<br>tCN_6^{eff} = 6	tCN_6(I) ≙ [6Li; 0]<br>[0.40, 0.32, 0.21]<br>tCN_6^{eff} = 6	{$Li^{[6;\,0]}$, $I^{[6;\,0]}$}<br>{0.63, 0.57, 0.46}<br>{0.82, 0.70, 0.52}°
(b)	tCN_6(Li) ≙ [6I; 0]<br>[1.00, 1.00, 1.00]<br>tCN_6^{eff} = 6	tCN_{18}(I) ≙ [6Li; 12I]<br>[0.60, 0.68, 0.79]<br>tCN_{18}^{eff} = 14.06	{$Li^{[6;\,0]}$, $I^{[6;\,12]}$}<br>{0.77, 0.82, 0.89}<br>**{1, 1, 1}**°
KI	K (V = 24.4 Å^3)	I (V = 67.4 Å^3)	
(a)	tCN_6(K) ≙ [6I; 0]<br>[1.00, 1.00, 1.00]<br>tCN_6^{eff} = 6	tCN_6(I) ≙ [6K; 0]<br>[0.83, 0.72, 0.65]<br>tCN_6^{eff} = 6	{$K^{[6;\,0]}$, $I^{[6;\,0]}$}<br>{0.91, 0.85, 0.81}<br>**{1, 1, 1}**°
(b)	tCN_6(K) ≙ [6I; 0]<br>[1.00, 1.00, 1.00]<br>tCN_6^{eff} = 6	tCN_{18}(I) ≙ [6K; 12I]<br>[0.17, 0.28, 0.35]<br>tCN_{18}^{eff} = 9.34	{$Li^{[6;\,0]}$, $I^{[6;\,12]}$}<br>{0.41, 0.53, 0.59}<br>{0.45, 0.62, 0.73}°
RbI	Rb (V = 32.9 Å^3)	I (V = 70.7 Å^3)	
(a)	tCN_6(Rb) ≙ [6Cl; 0]<br>[1.00, 1.00, 1.00]<br>tCN_6^{eff} = 6	tCN_6(I) ≙ [6Rb; 0]<br>[0.90, 0.81, 0.76]<br>tCN_6^{eff} = 6	{$Rb^{[6;\,0]}$, $I^{[6;\,0]}$}<br>{0.95, 0.90, 0.87}<br>**{1, 1, 1}**°
(b)	tCN_6(Rb) ≙ [6Cl; 0]<br>[1.00, 1.00, 1.00]<br>tCN_6^{eff} = 6	tCN_{18}(I) ≙ [6Rb; 12I]<br>[0.10, 0.19, 0.24]<br>tCN_{18}^{eff} = 8.28	{$Li^{[6;\,0]}$, $I^{[6;\,12]}$}<br>{0.32, 0.44, 0.49}<br>{0.34, 0.49, 0.56}°
RbF	Rb (V = 27.7 Å^3)	F (V = 19.5 Å^3)	
(a)	tCN_6(Rb) ≙ [6F; 0]<br>[0.99, 0.96, 0.95]<br>tCN_6^{eff} = 6	tCN_6(F) ≙ [6Rb; 0]<br>[1.00, 1.00, 1.00]<br>tCN_6^{eff} = 6	{$Rb^{[6;\,0]}$, $F^{[6;\,0]}$}<br>{0.99, 0.98, 0.97}<br>**{1, 1, 1}**°
(b)	tCN_{18}(Rb) ≙ [6F; 12Rb]<br>[0.01, 0.04, 0.05]<br>tCN_{18}^{eff} = 6.38	tCN_6(F) ≙ [6Rb; 0]<br>[1.00, 1.00, 1.00]<br>tCN_6^{eff} = 6	{$Rb^{[6;\,12]}$, $F^{[6;\,0]}$}<br>{0.10, 0.20, 0.22}<br>{0.10, 0.20, 0.23}°

CsCl type			
CsCl	Cs (V = 37.5 Å^3)	Cl (V = 36.4 Å^3)	
(a)	tCN_8(Cs) ≙ [8Cl; 0]<br>[0.81, 0.69, 0.62]<br>tCN_8^{eff} = 8	tCN_8(Cl) ≙ [8Cs; 0]<br>[0.80, 0.69, 0.61]<br>tCN_8^{eff} = 8	{$Cs^{[8;\,0]}$ $Cl^{[8;\,0]}$}<br>{0.80, 0.69, 0.61}<br>{**1, 1, 1**}°
(b)	tCN_{14}(Cs) ≙ [8Cl; 6Cs]<br>[0.19, 0.31, 0.38]<br>tCN_{14}^{eff} = 9.80	tCN_8(Cl) ≙ [8Cs; 0]<br>[0.80, 0.69, 0.61]<br>tCN_8^{eff} = 8	{$Cs^{[8;\,6]}$ $Cl^{[8;\,0]}$}<br>{0.39, 0.46, 0.48}<br>{0.49, 0.67, 0.79}°
(c)	tCN_8(Cs) ≙ [8Cl; 0]<br>[0.81, 0.69, 0.62]<br>tCN_8^{eff} = 8	tCN_{14}(Cl) ≙ [8Cs; 6Cl]<br>[0.20, 0.31, 0.39]<br>tCN_{14}^{eff} = 9.84	{$Cs^{[8;\,0]}$ $Cl^{[8;\,6]}$}<br>{0.40, 0.47, 0.49}<br>{0.50, 0.68, 0.80}°
(d)	tCN_{14}(Cs) ≙ [8Cl; 6Cs]<br>[0.19, 0.31, 0.38]<br>tCN_{14}^{eff} = 9.80	tCN_{14}(Cl) ≙ [8Cs; 6Cl]<br>[0.20, 0.31, 0.39]<br>tCN_{14}^{eff} = 9.84	{$Cs^{[8;\,6]}$ $Cl^{[8;\,6]}$}<br>{0.20, 0.31, 0.39}<br>{0.25, 0.45, 0.64}°
CsI	Cs (V = 42.1 Å^3)	I (V = 58.1 Å^3)	
(a)	tCN_8(Cs) ≙ [8I; 0]<br>[0.95, 0.87, 0.84]<br>tCN_8^{eff} = 8	tCN_8(I) ≙ [8Cs; 0]<br>[0.60, 0.49, 0.38]<br>tCN_8^{eff} = 8	{$Cs^{[8;\,0]}$, $I^{[8;\,0]}$}<br>{0.75, 0.65, 0.56}<br>{**1**, 0.97, 0.78}°
(b)	tCN_{14}(Cs) ≙ [8I; 6Cs]<br>[0.05, 0.13, 0.16]<br>tCN_{14}^{eff} = 8.76	tCN_8(I) ≙ [8Cs; 0]<br>[0.60, 0.49, 0.38]<br>tCN_8^{eff} = 8	{$Cs^{[8;\,6]}$, $I^{[8;\,0]}$}<br>{0.17, 0.25, 0.25}<br>{0.23, 0.37, 0.35}°
(c)	tCN_8(Cs) ≙ [8I; 0]<br>[0.95, 0.87, 0.84]<br>tCN_8^{eff} = 8	tCN_{14}(I) ≙ [8Cs; 6I]<br>[0.40, 0.51, 0.62]<br>tCN_{14}^{eff} = 11.08	{$Cs^{[8;\,0]}$, $I^{[8;\,6]}$}<br>{0.62, 0.67, 0.72}<br>{0.83, **1**, **1**}°
(d)	tCN_{14}(Cs) ≙ [8I; 6Cs]<br>[0.05, 0.13, 0.16]<br>tCN_{14}^{eff} = 8.76	tCN_{14}(I) ≙ [8Cs; 6I]<br>[0.40, 0.51, 0.62]<br>tCN_{14}^{eff} = 11.08	{$Cs^{[8;\,6]}$, $I^{[8;\,6]}$}<br>{0.14, 0.26, 0.31}<br>{0.19, 0.39, 0.43}°

Table S8. TiNiSi-type 3-species structures. For each species *A* the QTAIM volume *V*(*A*) is given. (Sub)-coordination scenarios are listed with increasing overall coordination. For each scenario ‘*scene*’ (a, b, …) and each species are listed: coordination notation, $tCN_{scene}(A)$, coordination likelihoods [^{sc2}w, ^{lin}w, ^{sc1}w], and $tCN_{scene}^{\mathrm{eff}}(A)$; the overall scenario is additionally characterized by scenario weights {^{sc2}W, ^{lin}W, ^{sc1}W} and normalized scenario weights {^{sc2}W, ^{lin}W, ^{sc1}W}°, where a value of **1** corresponds to the dominant scenario with highest scenario weight $^{scheme}W$ (denoted ‘max’ in the main text) for the respective weighting *scheme*. The last scenario given always corresponds to inclusion of all domain surfaces (denoted ‘tot’ in the main text).

Compound<br>*scene*	Species “*Si*”	Species “*Ti*”	Species “*Ni*”	Scenario<br>weights
TiNiSi	Si (*V* = 15.6 Å^3)	Ti (*V* = 10.4 Å^3)	Ni (*V* = 13.4 Å^3)	
(a)	tCN_7(Si) ≙ [3Ti, 4Ni; 0]<br>[0.1783, 0.1504, 0.1469]<br>tCN_7^{eff}= 5.95	tCN_3(Ti) ≙ [0, 3Si; 0]<br>[0.2584, 0.2051, 0.1253]<br>tCN_3^{eff}= 2.16	tCN_4(Ni) ≙ [0, 4Si; 0]<br>[0.6539, 0.5926, 0.6147]<br>tCN_4^{eff}= 3.78	{$\mathrm{Ti}^{[0,\,3;\,0]}$ $\mathrm{Ni}^{[0,\,4;\,0]}$ $\mathrm{Si}^{[3,\,4;\,0]}$}<br>{0.31, 0.26, 0.22}<br>{0.59, 0.51, 0.40}°
(b)	tCN_9(Si) ≙ [5Ti, 4Ni; 0]<br>[0.4510, 0.5214, 0.6242]<br>tCN_9^{eff}= 7.07	tCN_5(Ti) ≙ [0, 5Si; 0]<br>[0.4855, 0.4332, 0.4542]<br>tCN_5^{eff}= 3.29	tCN_4(Ni) ≙ [0, 4Si; 0]<br>[0.6540, 0.5926, 0.6147]<br>tCN_4^{eff}= 3.78	{$\mathrm{Ti}^{[0,\,5;\,0]}$ $\mathrm{Ni}^{[0,\,4;\,0]}$ $\mathrm{Si}^{[5,\,4;\,0]}$}<br>{0.52, 0.51, 0.56}<br>{**1, 1, 1**}°
(c)	tCN_{14}(Si) ≙ [5Ti, 5Ni; 4Si]<br>[0.0007, 0.0051, 0.0065]<br>tCN_{14}^{eff}= 7.19	tCN_{11}(Ti) ≙ [6Ni, 5Si; 0]<br>[0.0677, 0.1476, 0.1872]<br>tCN_{11}^{eff}= 4.39	tCN_{13}(Ni) ≙ [6Ti, 5Si; 2Ni]<br>[0.0097, 0.0395, 0.0502]<br>tCN_{13}^{eff}= 5.38	{$\mathrm{Ti}^{[6,\,5;\,0]}$ $\mathrm{Ni}^{[6,\,5;\,2]}$ $\mathrm{Si}^{[5,\,5;\,4]}$}<br>{0.01, 0.03, 0.04}<br>{0.02, 0.06, 0.07}°
Co_2Si	Si (*V* = 10.6 Å^3)	Co1 (*V* = 10.8 Å^3)	Co2 (*V* = 11.1 Å^3)	
(a)	tCN_6(Si) ≙ [2Co1, 4Co2; 0]<br>[0.1743, 0.1395, 0.1028]<br>tCN_6^{eff}= 5.60	tCN_2(Co1) ≙ [0, 2Si; 0]<br>[0.2011, 0.1586, 0.0744]<br>tCN_2^{eff}= 1.77	tCN_4(Co2) ≙ [0, 4Si; 0]<br>[0.5497, 0.4571, 0.3983]<br>tCN_4^{eff} = 3.83	{$\mathrm{Co1}^{[0,\,2;\,0]}$ $\mathrm{Co2}^{[0,\,4;\,0]}$ $\mathrm{Si}^{[2,\,4;0]}$}<br>{0.27, 0.22, 0.15}<br>{0.82, 0.80, 0.44}°
(b)	tCN_7(Si) ≙ [3Co1, 4Co2; 0]<br>[0.2317, 0.1950, 0.1888]<br>tCN_7^{eff}= 6.34	tCN_3(Co1) ≙ [0, 3Si; 0]<br>[0.2704, 0.2211, 0.1905]<br>tCN_3^{eff}= 2.51	tCN_4(Co2) ≙ [0, 4Si; 0]<br>[0.5497, 0.4571, 0.3983]<br>tCN_4^{eff}= 3.83	{$\mathrm{Co1}^{[0,\,3;\,0]}$ $\mathrm{Co2}^{[0,\,4;\,0]}$ $\mathrm{Si}^{[3,\,4;0]}$}<br>{0.33, 0.27, 0.24}<br>{**1, 1**, 0.73}°
(c)	tCN_9(Si) ≙ [5Co1, 4Co2; 0]<br>[0.1009, 0.0914, 0.1007]<br>tCN_9^{eff}= 7.44	tCN_7(Co1) ≙ [0, 5Si; 2Co1]<br>[0.0899, 0.0780, 0.0811]<br>tCN_7^{eff}= 4.69	tCN_4(Co2) ≙ [0, 4Si; 0]<br>[0.5497, 0.4571, 0.3983]<br>tCN_4^{eff}= 3.83	{$\mathrm{Co1}^{[0,\,5;\,2]}$ $\mathrm{Co2}^{[0,\,4'\,0]}$ $\mathrm{Si}^{[5,\,4;0]}$}<br>{0.17, 0.15, 0.15}<br>{0.52, 0.55, 0.45}°
(d)	tCN_{10}(Si) ≙ [5Co1, 5Co2; 0]	tCN_{10}(Co1) ≙ [3Co2, **5Si**; 2Co1]	tCN_8(Co2) ≙ [3Co1, 5Si; 0]	{$\mathrm{Co1}^{[3,\,5;\,2]}$ $\mathrm{Co2}^{[3,\,5;\,0]}$ $\mathrm{Si}^{[5,\,5;0]}$}

	[0.3357, 0.4226, 0.5173]<br>tCN_{10}^{eff}= 7.89	[0.1180, 0.1131, 0.1300]<br>tCN_{10}^{eff}= 6.02	[0.0984, 0.0987, 0.1162]<br>tCN_{8}^{eff}= 5.62	{0.16, 0.17, 0.20}<br>{0.48, 0.62, 0.60}°
(e)	tCN_{10}(Si) ≙ [5Co1, 5Co2; 0]<br>[0.3357, 0.4226, 0.5173]<br>tCN_{10}^{eff}= 7.89	tCN_{13}(Co1) ≙ [6Co2, 5Si; 2Co1]<br>[0.1297, 0.2140, 0.2697]<br>tCN_{13}^{eff}= 6.89	tCN_{13}(Co2) ≙ [6Co1, 5Si; 2Co2]<br>[0.1080, 0.2051, 0.2593]<br>tCN_{13}^{eff}= 6.90	{Co1$^{[6, 5; 2]}$ Co2$^{[6, 5; 2]}$ Si$^{[5, 5;0]}$}<br>{0.17, 0.26, 0.33}<br>{0.51, 0.98, **1**}°
(f)	tCN_{15}(Si) ≙ [6Co1, 5Co2; 4Si]<br>[0.0028, 0.0177, 0.0225]<br>tCN_{15}^{eff}= 8.02	tCN_{14}(Co1) ≙ [6Co2, 6Si; 2Co1]<br>[0.0044, 0.0237, 0.0302]<br>tCN_{14}^{eff}= 6.91	tCN_{13}(Co2] ≙ [6Co1, 5Si; 2Co2]<br>[0.1080, 0.2051, 0.2593]<br>tCN_{13}^{eff}= 6.90	{Co1$^{[6, 6; 2]}$ Co2$^{[6, 5; 2]}$ Si$^{[6, 5;4]}$}<br>{0.01, 0.04, 0.06}<br>{0.03, 0.16, 0.17}°
SrLiAs	As (*V* = 45.0 Å^3)	Sr (*V* = 21.2 Å^3)	Li (*V* = 4.3 Å^3)	
(a)	tCN_9(As) ≙ [5Sr, 4Li; 0]<br>[0.3061, 0.2641, 0.2683]<br>tCN_{9}^{eff}= 7.75	tCN_5(Sr) ≙ [0, 5As; 0]<br>[0.4199, 0.3521, 0.3281]<br>tCN_{5}^{eff}= 4.21	tCN_4(Li) ≙ [0, 4As; 0]<br>[0.6569, 0.7265, 0.8354]<br>tCN_{4}^{eff}= 3.54	{Sr$^{[0, 5; 0]}$ Li$^{[0, 4; 0]}$ As$^{[5,4; 0]}$}<br>{0.44, 0.41, 0.42}<br>{**1**, 0.94, 0.82}°
(b)	tCN_{10}(As) ≙ [6Sr, 4Li; 0]<br>[0.2194, 0.2286, 0.2720]<br>tCN_{10}^{eff}= 8.22	tCN_6(Sr) ≙ [0, 6As; 0]<br>[0.3810, 0.4886, 0.5960]<br>tCN_{6}^{eff}= 4.68	tCN_4(Li) ≙ [0, 4As; 0]<br>[0.6569, 0.7265, 0.8354]<br>tCN_{4}^{eff}= 3.54	{Sr$^{[0, 6; 0]}$ Li$^{[0, 4; 0]}$ As$^{[6,4; 0]}$}<br>{0.38, 0.43, 0.51}<br>{0.87, **1, 1**}°
(c)	tCN_{20}(As) ≙ [6Sr, 4Li; 10As]<br>[0.0494, 0.1089, 0.1383]<br>tCN_{20}^{eff}= 9.77	tCN_6(Sr) ≙ [0, 6As; 0]<br>[0.3810, 0.4886, 0.5960]<br>tCN_{6}^{eff}= 4.68	tCN_4(Li) ≙ [0, 4As; 0]<br>[0.6569, 0.7265, 0.8354]<br>tCN_{4}^{eff}= 3.54	{Sr$^{[0, 6; 0]}$ Li$^{[0, 4; 0]}$ As$^{[6,4; 10]}$}<br>{0.23, 0.34, 0.41}<br>{0.53, 0.78, 0.80}°
(d)	tCN_{22}(As) ≙ [6Sr, 4Li; 12As]<br>[0.0021, 0.0147, 0.0188]<br>tCN_{22}^{eff}= 9.80	tCN_6(Sr) ≙ [0, 6As; 0]<br>[0.3810, 0.4886, 0.5960]<br>tCN_{6}^{eff}= 4.68	tCN_4(Li) ≙ [0, 4As; 0]<br>[0.6569, 0.7265, 0.8354]<br>tCN_{4}^{eff}= 3.54	{Sr$^{[0, 6; 0]}$ Li$^{[0, 4; 0]}$ As$^{[6,4; 12]}$}<br>{0.08, 0.17, 0.21}<br>{0.19, 0.40, 0.41}°
$PbCl_2$	Pb (*V* = 23.7 Å^3)	Cl1 (*V* = 28.0 Å^3)	Cl2 (*V* = 26.4 Å^3)	
(a)	tCN_6(Pb) ≙ [3Cl1, 3Cl2; 0]<br>[0.0760, 0.0605, 0.0417]<br>tCN_{6}^{eff}= 5.55	tCN_3(Cl1) ≙ [3Pb, 0; 0]<br>[0.4924, 0.41462, 0.3857]<br>tCN_{3}^{eff}= 2.74	tCN_3(Cl2) ≙ [3Pb, 0; 0]<br>[0.1157, 0.0914, 0.0516]<br>tCN_{3}^{eff}= 2.80	{Pb$^{[3, 3; 0]}$ Cl1$^{[3, 0; 0]}$} Cl2$^{[3, 0; 0]}$}<br>{0.16, 0.13, 0.09}<br>{0.35, 0.33, 0.25}°
(b)	tCN_7(Pb) ≙ [3Cl1, 4Cl2; 0]<br>[0.4752, 0.4105, 0.4088]	tCN_3(Cl1) ≙ [3Pb, 0; 0]<br>[0.4924, 0.41462, 0.3857]	tCN_4(Cl2) ≙ [4Pb, 0; 0]<br>[0.4430, 0.3724, 0.3484]	{Pb$^{[3, 4; 0]}$ Cl1$^{[3, 0; 0]}$} Cl2$^{[4, 0; 0]}$}<br>{0.47, 0.40, 0.38}

	tCN_7^{eff}= 6.35	tCN_3^{eff}= 2.74	tCN_4^{eff}= 3.61	{**1, 1, 1**}°
(c)	*tCN*$_9$(Pb) ≙ [5Cl1, 4Cl2; 0]<br>[0.2835, 0.3967, 0.4913]<br>tCN_9^{eff}= 7.15	*tCN*$_9$(Cl1) ≙ [5Pb, 4Cl2; 0]<br>[0.1054, 0.1100, 0.1315]<br>tCN_9^{eff}= 5.30	*tCN*$_8$(Cl2) ≙ [4Pb, 4Cl1; 0]<br>[0.0328, 0.0313, 0.0360]<br>tCN_8^{eff}= 5.38	{Pb$^{[5, 4; 0]}$ Cl1$^{[5, 4; 0]}$} Cl2$^{[4, 4; 0]}$}<br>{0.10, 0.11, 0.13}<br>{0.21, 0.28, 0.35}°
(d)	*tCN*$_9$(Pb) ≙ [5Cl1, 4Cl2; 0]<br>[0.2835, 0.3967, 0.4913]<br>tCN_9^{eff}= 7.15	*tCN*$_9$(Cl1) ≙ [5Pb, 4Cl2; 0]<br>[0.1054, 0.1100, 0.1315]<br>tCN_9^{eff}= 5.30	*tCN*$_{10}$(Cl2) ≙ [4Pb, 4Cl1; 2Cl2]<br>[0.1099, 0.1128, 0.1340]<br>tCN_{10}^{eff}= 6.17	{Pb$^{[5, 4; 0]}$ Cl1$^{[5, 4; 0]}$} Cl2$^{[4, 4; 2]}$}<br>{0.15, 0.17, 0.21}<br>{0.32, 0.43, 0.54}°
(e)	*tCN*$_9$(Pb) ≙ [5Cl1, 4Cl2; 0]<br>[0.2835, 0.3967, 0.4913]<br>tCN_9^{eff}= 7.15	*tCN*$_{13}$(Cl1) ≙ [5Pb, 6Cl2; 2Cl1]<br>[0.0691, 0.1006, 0.1263]<br>tCN_{13}^{eff}= 6.27	*tCN*$_{12}$(Cl2) ≙ [4Pb, 6Cl1; 2Cl2]<br>[0.1207, 0.2205, 0.2784]<br>tCN_{12}^{eff}= 6.67	{Pb$^{[5, 4; 0]}$ Cl1$^{[5, 6; 2]}$} Cl2$^{[4, 6; 2]}$}<br>{0.13, 0.21, 0.26}<br>{0.28, 0.52, 0.68}°
(f)	*tCN*$_9$(Pb) ≙ [5Cl1, 4Cl2; 0]<br>[0.2835, 0.3967, 0.4913]<br>tCN_9^{eff}= 7.15	*tCN*$_{15}$(Cl1) ≙ [5Pb, 6Cl2; 4Cl1]<br>[0.0421, 0.1052, 0.1337]<br>tCN_{15}^{eff}= 6.49	*tCN*$_{12}$(Cl2) ≙ [4Pb, 6Cl1; 2Cl2]<br>[0.1207, 0.2205, 0.2784]<br>tCN_{12}^{eff}= 6.67	{Pb$^{[5, 4; 0]}$ Cl1$^{[5, 6; 4]}$} Cl2$^{[4, 6; 2]}$}<br>{0.11, 0.21, 0.26}<br>{0.24, 0.53, 0.69}°
$BaCl_2$	Ba (*V* = 27.0 Å^3)	Cl1 (*V* = 33.6 Å^3)	Cl2 (*V* = 30.2 Å^3)	
(a)	*tCN*$_7$(Ba) ≙ [3Cl1, 4Cl2; 0]<br>[0.4733, 0.3916, 0.3418]<br>tCN_7^{eff}= 6.59	*tCN*$_3$(Cl1) ≙ [3Ba, 0Cl2; 0]<br>[0.5006, 0.4059, 0.3036]<br>tCN_3^{eff}= 2.74	*tCN*$_4$(Cl2) ≙ [4Ba, 0Cl1; 0]<br>[0.4496, 0.3704, 0.3186]<br>tCN_4^{eff}= 3.85	{Ba$^{[3, 4; 0]}$ Cl1$^{[3, 0; 0]}$} Cl2$^{[4, 0; 0]}$}<br>{0.47, 0.39, 0.32}<br>{**1, 1**, 0.83}°
(b)	*tCN*$_9$(Ba) ≙ [5Cl1, 4Cl2; 0]<br>[0.4053, 0.5125, 0.6227]<br>tCN_9^{eff}= 7.62	*tCN*$_9$(Cl1) ≙ [5Ba, 4Cl2; 0]<br>[0.1588, 0.1459, 0.1624]<br>tCN_9^{eff}= 5.89	*tCN*$_{10}$(Cl2) ≙ [4Ba, 4Cl1; 2Cl2]<br>[0.1182, 0.1128, 0.1293]<br>tCN_{10}^{eff}= 6.95	{Ba$^{[5, 4; 0]}$ Cl1$^{[5, 4; 0]}$} Cl2$^{[4, 4; 2]}$}<br>{0.20, 0.20, 0.24}<br>{0.41, 0.52, 0.61}°
(c)	*tCN*$_9$(Ba) ≙ [5Cl1, 4Cl2; 0]<br>0.4053, 0.5125, 0.6227]<br>tCN_9^{eff}= 7.62	*tCN*$_{15}$(Cl1) ≙ [5Ba, 6Cl2; 4Cl1]<br>[0.1020, 0.1890, 0.2390]<br>tCN_{15}^{eff}= 7.48	*tCN*$_{12}$(Cl2) ≙ [4Ba, 6Cl1; 2Cl2]<br>[0.2029, 0.3152, 0.3945]<br>tCN_{12}^{eff}= 7.65	{Ba$^{[5, 4; 0]}$ Cl1$^{[5, 6; 4]}$} Cl2$^{[4, 6; 2]}$}<br>{0.20, 0.31, 0.39}<br>{0.43, 0.80, **1**}°
(d)	*tCN*$_9$(Ba) ≙ [5Cl1, 4Cl2; 0]<br>[0.4053, 0.5125, 0.6227]<br>tCN_9^{eff}= 7.62	*tCN*$_{17}$(Cl1) ≙ [5Ba, 6Cl2; 6Cl1]<br>[0.0012, 0.0096, 0.0123]<br>tCN_{17}^{eff}= 7.50	*tCN*$_{12}$(Cl2) ≙ [4Ba, 6Cl1; 2Cl2]<br>[0.2029, 0.3152, 0.3945]<br>tCN_{12}^{eff}= 7.65	{Ba$^{[5, 4; 0]}$ Cl1$^{[5, 6; 6]}$} Cl2$^{[4, 6; 2]}$}<br>{0.05, 0.12, 0.14}<br>{0.09, 0.30, 0.37}°
Ba_2Ge	Ge (*V* = 49.8 Å^3)	Ba1 (*V* = 35.3 Å^3)	Ba2 (*V* = 33.4 Å^3)	

(a)	*tCN*$_7$(Ge) ≙ [3Ba1, 4Ba2; 0]<br>[0.3378, 0.2804, 0.2559]<br>tCN_7^{eff}= 6.49	*tCN*$_3$(Ba1) ≙ [0, 3Ge; 0]<br>[0.3366, 0.2794, 0.2550]<br>tCN_3^{eff}= 2.66	*tCN*$_4$(Ba2) ≙ [0, 4Ge; 0]<br>[0.8541, 0.7643, 0.7323]<br>tCN_4^{eff}= 3.84	{Ba1$^{[0, 3; 0]}$ Ba2$^{[0, 4; 0]}$ Ge$^{[3, 4; 0]}$}<br>{0.46, 0.39, 0.36}<br>{0.98, 0.85, 0.71}°
(b)	*tCN*$_9$(Ge) ≙ [5Ba1, 4Ba2; 0]<br>[0.3611, 0.3759, 0.4431]<br>tCN_9^{eff}= 7.59	*tCN*$_5$(Ba1) ≙ [0, 5Ge; 0]<br>[0.3382, 0.3452, 0.4047]<br>tCN_5^{eff}= 3.75	*tCN*$_4$(Ba2) ≙ [0, 4Ge; 0]<br>[0.8541, 0.7643, 0.7323]<br>tCN_4^{eff}= 3.84	{Ba1$^{[0, 5; 0]}$ Ba2$^{[0, 4; 0]}$ Ge$^{[5, 4; 0]}$}<br>{0.47, 0.46, 0.51}<br>{**1, 1, 1**}°
(c)	*tCN*$_{15}$(Ge) ≙ [6Ba1, 5Ba2; 4Ge]<br>[0.0011, 0.0075, 0.0095]<br>tCN_{15}^{eff}= 7.96	*tCN*$_{14}$(Ba1) ≙ [6Ba2, 6Ge; 2Ba1]<br>[0.0051, 0.0246, 0.0313]<br>tCN_{14}^{eff}= 4.82	*tCN*$_{13}$(Ba2) ≙ [6Ba1, 5Ge; 2Ba2]<br>[0.0148, 0.0536, 0.0682]<br>tCN_{13}^{eff}= 4.85	{Ba1$^{[6,6;2]}$ Ba2$^{[6,5;2]}$ Ge$^{[6,5;4]}$}<br>{0.009, 0.046, 0.054}<br>{0.004, 0.021, 0.027}°
Ca_2Ge	Ge (*V* = 49.8 Å^3)	Ca1 (*V* = 17.9 Å^3)	Ca2 (*V* = 16.9 Å^3)	
(a)	*tCN*$_7$(Ge) ≙ [3Ca1, 4Ca2; 0]<br>[0.2359, 0.1956, 0.1803]<br>tCN_7^{eff}= 6.35	*tCN*$_3$(Ca1) ≙ [0, 3Ge; 0]<br>[0.2625, 0.2135, 0.1784<br>tCN_3^{eff}= 2.48]	*tCN*$_4$(Ca2) ≙ [0, 4Ge; 0]<br>[0.8505, 0.8607, 0.9223]<br>tCN_4^{eff}= 3.86	{Ca1$^{[0, 3; 0]}$ Ca2$^{[0, 4; 0]}$ Ge$^{[3, 4; 0]}$}<br>{0.37, 0.33, 0.31}<br>{0.71, 0.62, 0.52}°
(b)	*tCN*$_9$(Ge) ≙ [Ca1, 4Ca2; 0]<br>[0.3921, 0.3968, 0.4615]<br>tCN_9^{eff}= 7.52	*tCN*$_5$(Ca1) ≙ [0, 5Ge; 0]<br>[0.4427, 0.4338, 0.4946]<br>tCN_5^{eff}= 3.66	*tCN*$_4$(Ca2) ≙ [0, 4Ge; 0]<br>[0.8505, 0.8607, 0.9223]<br>tCN_4^{eff}= 3.86	{Ca1$^{[0, 5; 0]}$ Ca2$^{[0, 4; 0]}$ Ge$^{[5, 4; 0]}$}<br>{0.53, 0.53, 0.59}<br>{**1, 1, 1**}°
(c)	*tCN*$_{23}$(Ge) ≙ [6Ca1,5Ca2;12Ge]<br>[0.0048, 0.0253, 0.0322]<br>tCN_{23}^{eff}= 8.71	*tCN*$_8$(Ca1) ≙ [2Ca2, 6Ge; 0]<br>[0.0008, 0.0068, 0.0086]<br>tCN_8^{eff}= 3.86	*tCN*$_7$(Ca2) ≙ [2Ca1, 5Ge; 0]<br>[0.0007, 0.0067, 0.0085]<br>tCN_7^{eff}= 3.90	{Ca1$^{[2, 6;0]}$ Ca2$^{[2, 5; 0]}$ Ge$^{[6, 5; 12}$}<br>{0.001, 0.010, 0.013}<br>{0.003, 0.020, 0.022}°
BaH_2	Ba (*V* = 28.1 Å^3)	H1 (*V* = 15.0 Å^3)	H2 (*V* = 12.1 Å^3)	
(a)	*tCN*$_5$(Ba) ≙ [1H1, 4H2; 0]<br>[0.1492, 0.1184, 0.0758]<br>tCN_5^{eff}= 4.85	*tCN*$_1$(H1) ≙ [1Ba, 0; 0]<br>[0.1626, 0.1281, 0.0540]<br>tCN_1^{eff}= 0.92	*tCN*$_4$(H2) ≙ [4Ba, 0; 0)<br>[0.8131, 0.7357, 0.7293]<br>tCN_4^{eff}= 3.93	{Ba$^{[1, 4; 0]}$ H1$^{[1, 0; 0]}$ H2$^{[4, 0; 0]}$}<br>{0.27, 0.22, 0.14}<br>{0.42, 0.36, 0.22}°
(b)	*tCN*$_7$(Ba) ≙ [3H1, 4H2; 0]<br>[0.1531, 0.1246, 0.1067]<br>tCN_7^{eff}= 6.46	*tCN*$_3$(H1) ≙ [3Ba, 0; 0]<br>[0.1703, 0.1366, 0.1028]<br>tCN_3^{eff}= 2.52	*tCN*$_4$(H2) ≙ [4Ba, 0; 0]<br>[0.8131, 0.7357, 0.7293]<br>tCN_4^{eff}= 3.93	{Ba$^{[3, 4; 0]}$ H1$^{[3, 0; 0]}$ H2$^{[4, 0; 0]}$}<br>{0.28, 0.23, 0.20}<br>{0.43, 0.38, 0.30}°
(c)	*tCN*$_9$(Ba) ≙ [5H1, 4H2; 0]	*tCN*$_5$(H1) ≙ [5Ba, 0; 0]	*tCN*$_4$(H2) ≙ [4Ba, 0; 0)	{Ba$^{[5, 4; 0]}$ H1$^{[5, 0; 0]}$ H2$^{[4, 0; 0]}$}

	[0.5765, 0.6144, 0.7114]<br>tCN_9^{eff}= 7.81	[0.5504, 0.5195, 0.5708]<br>tCN_5^{eff}= 3.88	[0.8131, 0.7357, 0.7293]<br>tCN_4^{eff}= 3.93	{0.64, 0.62, 0.67}<br>{**1, 1, 1**}°
(d)	tCN_{15}(Ba) ≙ [6H1, 5H2; 4Ba]<br>[0.0007, 0.0074, 0.0095]<br>tCN_{15}^{eff}= 7.97	tCN_{16}(H1) ≙ [6Ba, 6H2; 4H1]<br>[0.0054, 0.0273, 0.0348]<br>tCN_{16}^{eff}= 4.98	tCN_{13}(H2) ≙ [5Ba, 6H1; 2H2]<br>[0.0016, 0.0121, 0.0154]<br>tCN_{13}^{eff}= 4.93	{$Ba^{[6, 5; 4]}$ $H1^{[6, 6; 4]}$ $H2^{[5, 6; 2]}$}<br>{0.002, 0.013, 0.017}<br>{0.003, 0.022, 0.026}°
